# Supplementary material for: Discovery and preclinical development of a therapeutically active nanobody-based chimeric antigen receptor targeting human CD22
Source: Mol Ther Oncol. 2024 Feb 13;32(1):200775. doi: 10.1016/j.omton.2024.200775 (PMC10914482; doi:10.1016/j.omton.2024.200775)
Supplement: Document S1. Figures S1–S12 and Tables S1, S2, and S4 [file mmc1.pdf]

OMTON, Volume *32*

Supplemental information

Discovery and preclinical development of a therapeutically active nanobody-based chimeric antigen receptor targeting human CD22

Scott McComb, Mehdi Arbabi-Ghahroudi, Kevin A. Hay, Brian A. Keller, Sharlene Faulkes, Michael Rutherford, Tina Nguyen, Alex Shepherd, Cunle Wu, Anne Marcil, Annie Aubry, Greg Hussack, Devanand M. Pinto, Shannon Ryan, Shalini Raphael, Henk van Faassen, Ahmed Zafer, Qin Zhu, Susanne Maclean, Anindita Chattopadhyay, Komal Gurnani, Rénald Gilbert, Christine Gadoury, Umar Iqbal, Dorothy Fatehi, Anna Jezierski, Jez Huang, Robert A. Pon, Mhairi Sigrist, Robert A. Holt, Brad H. Nelson, Harold Atkins, Natasha Kekre, Eric Yung, John Webb, Julie S. Nielsen, and Risini D. Weeratna

Supplemental Figures:

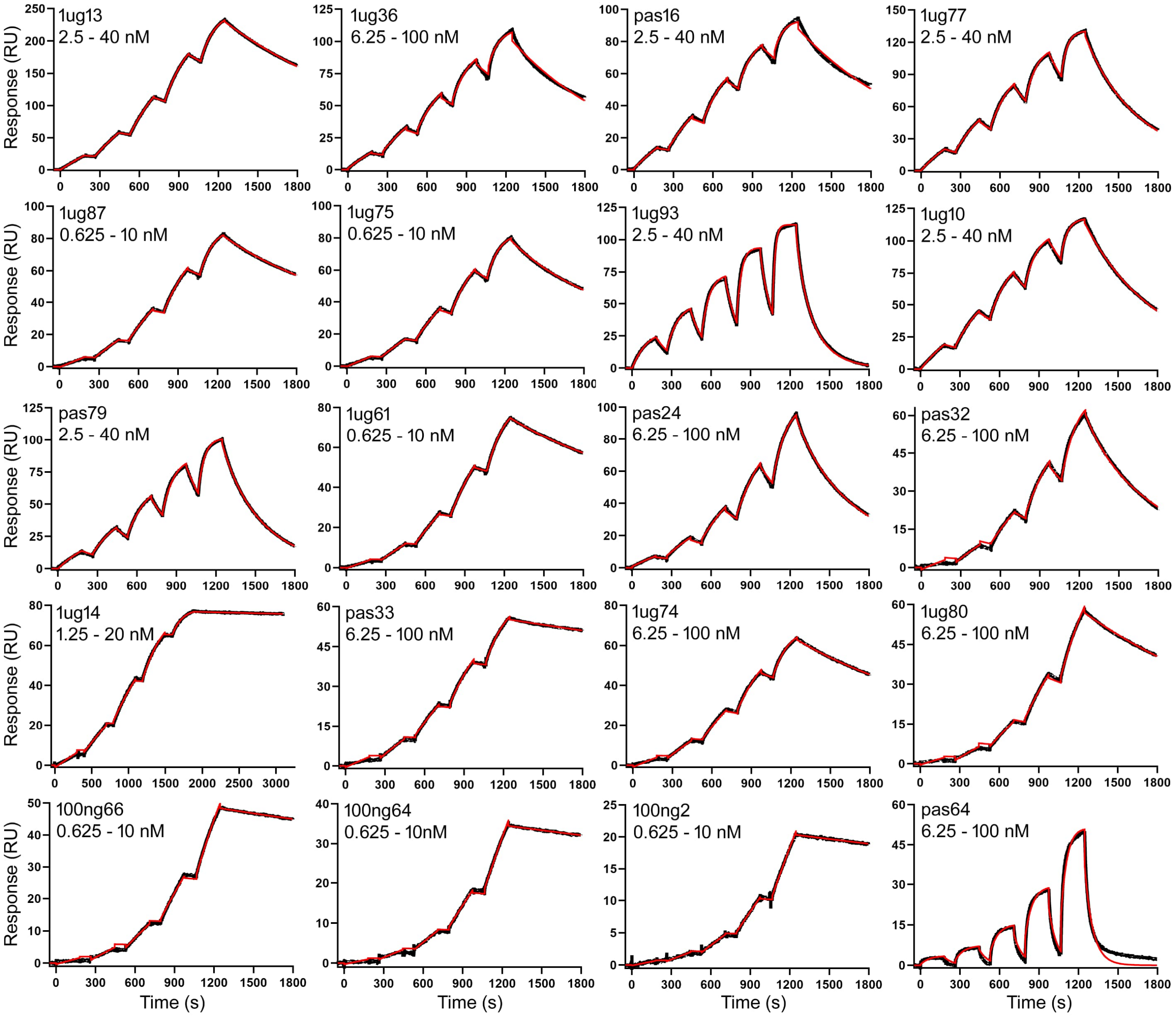

Figure S1: *Surface plasmon resonance sensorgrams for 20 CD22-specific single domain antibodies.* Human CD22 was amine coupled on a Series S CM5 sensor chip and SEC-purified sdAb monomers were injected at the concentration ranges indicated. Reference flow cell subtracted single-cycle kinetics sensorgrams were fit to a 1:1 interaction mode to determine kinetics and affinities (*see* Table S1). Black lines represent raw data, red lines represent 1:1 binding model fitting.

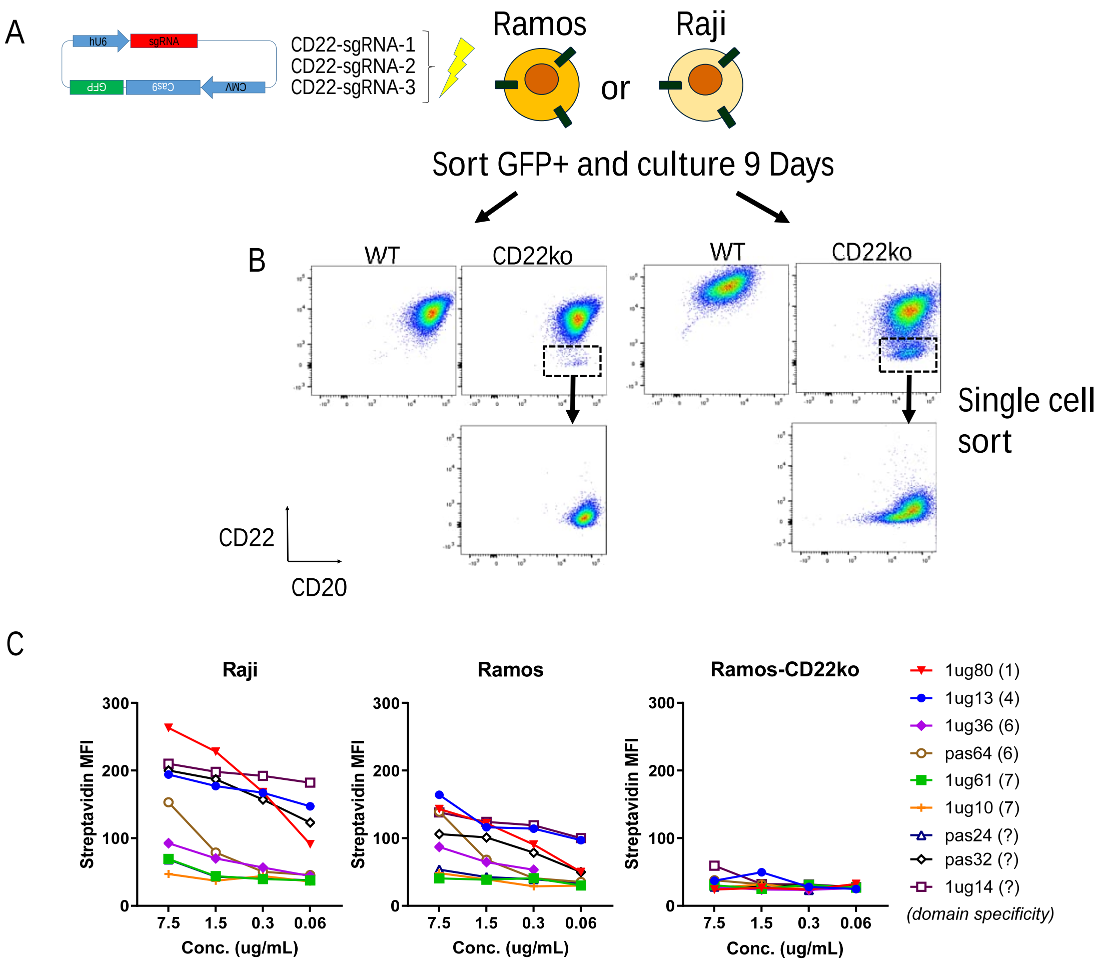

<u>Figure S2:</u> *Generating CD22-Knockout Raji and Ramos cell lines with CRISPR plasmids.* (a) Plasmids encoding 3 sgRNA sequences targeting the human CD22 gene were generated and electroporated into Ramos and Raji cells. After several days in culture, flow cytometry with anti-human CD22 and CD20 was performed, revealing a stable population of CD22 knockout cells. Cells were then single cell sorted to generate clonal knockout lines which were also (b) confirmed by flow cytometry to have no detectable CD22 expression, but maintained CD20 expression, which is characteristic of these lymphoma lines. (c) Cell binding with various biotinylated anti-CD22 sdAbs was performed to confirm staining against Raji and Ramos cells, but no staining against Ramos-CD22ko cells.

A

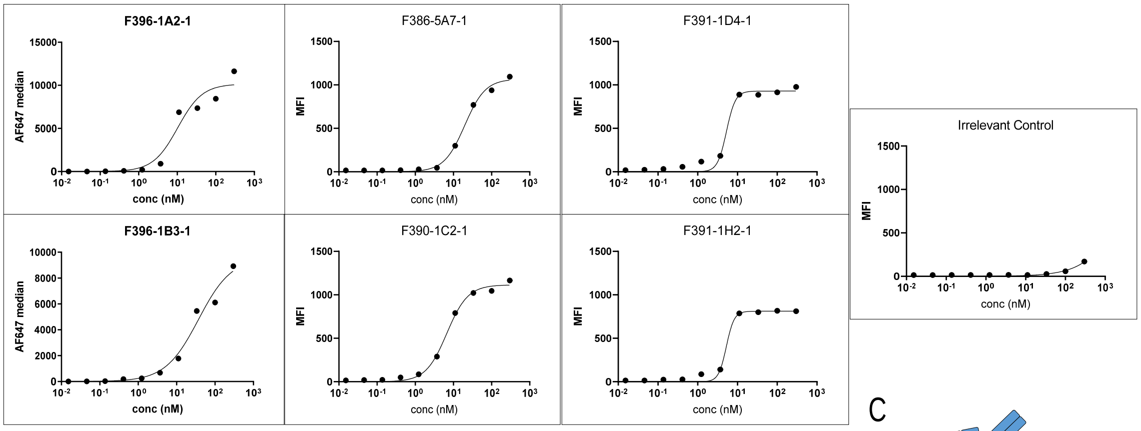

Yeast-surface Display Screening

B

Clone ID	Apparent Kd in Ramos Cell Binding	Bin	Membrane Distal → Membrane Proximal							Full Length CD22
			Domain 1	Domain 2	Domain 3	Domain 4	Domain 5	Domain 6	Domain 7	
F386-5A7-1	20.1	1	1.167							0.498
F391-1D4-1	5.3	2		0.503						0.840
F396-1B3-1	37.0	3			1.086					0.278
F390-1C2-1	6.8	3			1.415					0.706
F391-1H2-1	5.3	4				1.559				1.256
F396-1A2-1	10.3	4				1.922				1.271

C

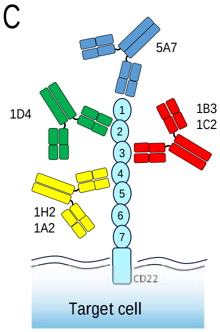

Figure S3: *Confirmation of cell binding and domain specificity for mouse anti-human CD22 monoclonal antibodies.* Mouse monoclonal antibodies targeting human CD22 were generated via immunization and hybridoma screening. (a) 6 anti-CD22 antibodies were purified and screened for cell binding against CD22-expressing Ramos cells, using a fluorescently labelled anti-mouse secondary antibody for detection. Cells were then examined via flow cytometry. (b) Purified antibodies were examined for binding to the CD22-domain yeast-surface display library similarly as described in the methods section. The binding to specific CD22-domains was assessed via yeast ELISA. The table shows the relative signal for the highest binding domain for each antibody tested. (c) Provides an overview of the antibody specificity for those mouse anti-CD22 antibodies tested here.

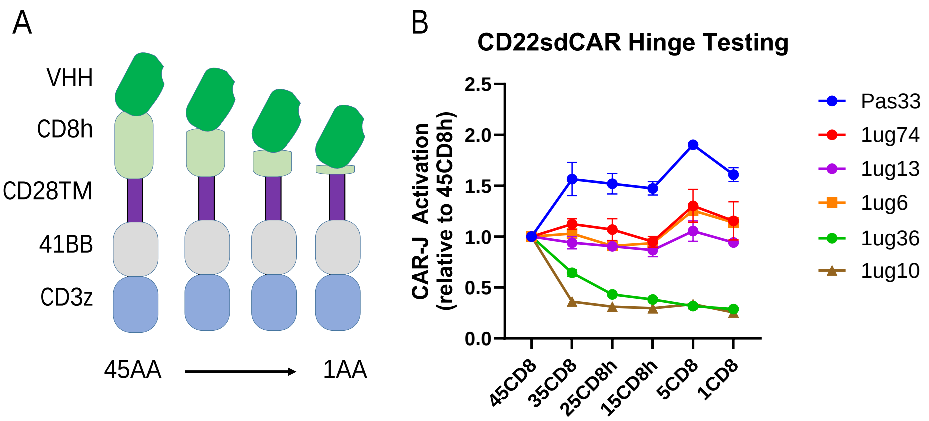

Figure S4: *CAR-T Activity Screening in Primary Human T cells.* T cells were isolated from healthy donor PBMC, polyclonally activated, and transduced with various CD22sdCAR lentivirus. At 9 to 10 days after polyclonal activation, green-fluorescent CAR-T cells or un-transduced T cells (*mock*) were placed in co-culture with red-fluorescent CD22+ target cells, Raji or Ramos, or CD22-deficient Ramos-CD22ko cells, at (a-b) a total T-cell to target ratio of 5:1 with IL-2 cytokine or (c) a 25:1 ratio with IL7/15 cytokines, with a constant 2000 target cells per well in a 96-well plate. Co-cultures were tracked via live microscopy at regular intervals to assess target cell growth (*top graphs*) and CAR-T growth (*bottom graphs*). Each graph presents the mean of two duplicate wells from a single experiment. (d) CD22sdCAR-T cells were also tested for short-term cytolytic response via chromium release assay as described in the methods section. Results show the mean of a single experiment performed in duplicate for two donors as shown, (e) graph shows the mean cytolysis at 40:1 E:T ratio from both donors +/- SEM, P-values shows the result of a student T test comparison of specific Raji killing with unmodified (mock) T cells.

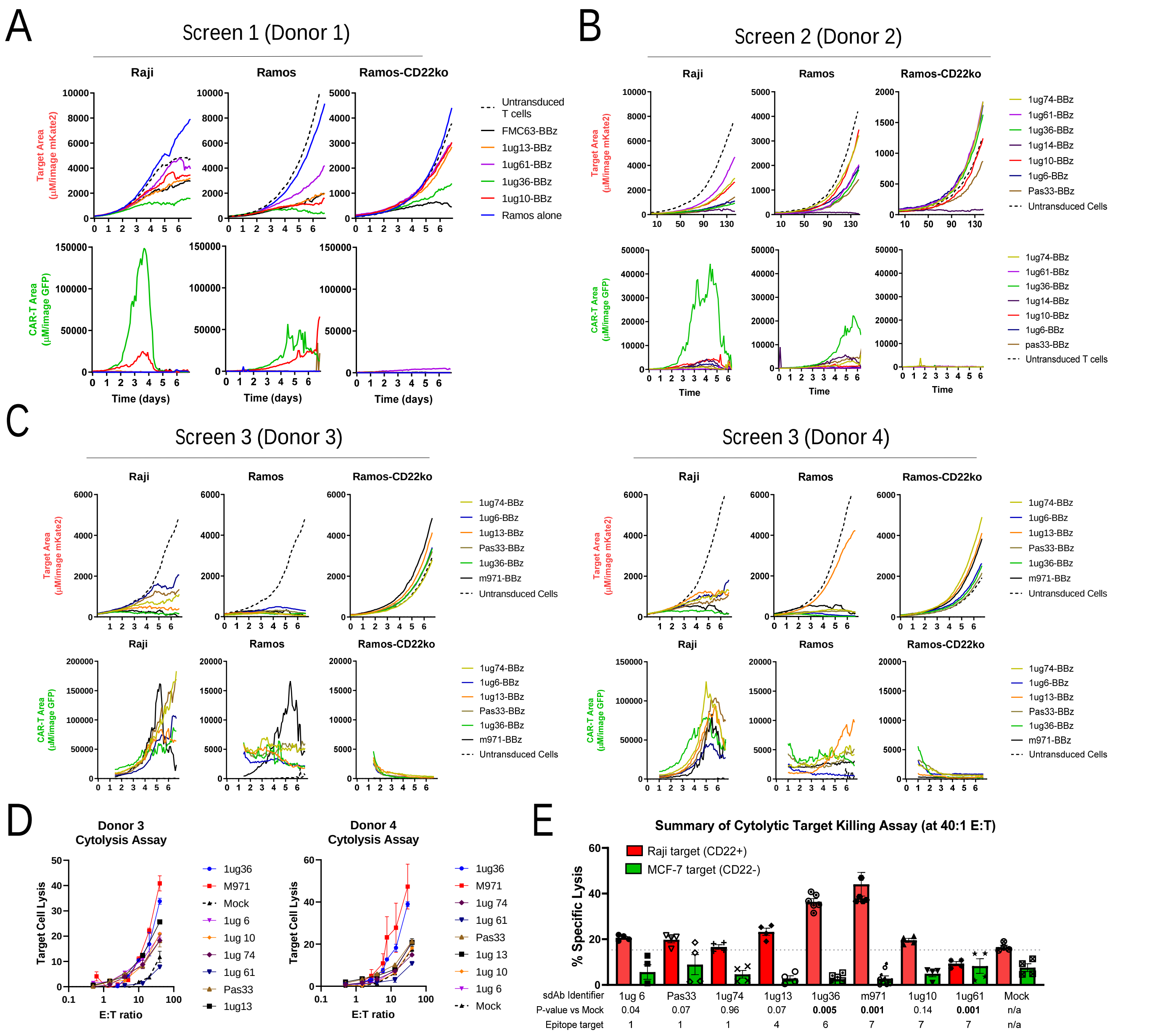

sdAb Identifier	1ug 6	Pas33	1ug74	1ug13	1ug36	m971	1ug10	1ug61	Mock
P-value vs Mock	0.04	0.07	0.96	0.07	**0.005**	**0.001**	0.14	**0.001**	n/a
Epitope target	1	1	1	4	6	7	7	7	n/a

<u>Figure S5</u>: *CAR-T Activity Screening in Primary Human T cells.* T cells were isolated from healthy donor PBMC, polyclonally activated, and transduced with various CD22sdCAR lentivirus. At 9 to 10 days after polyclonal activation, green-fluorescent CAR-T cells or un-transduced T cells (*mock*) were placed in co-culture with red-fluorescent CD22+ target cells, Raji or Ramos, or CD22-deficient Ramos-CD22ko cells, at (a-b) a total T-cell to target ratio of 5:1 with IL-2 cytokine or (c) a 25:1 ratio with IL7/15 cytokines, with a constant 2000 target cells per well in a 96-well plate. Co-cultures were tracked via live microscopy at regular intervals to assess target cell growth (*top graphs*) and CAR-T growth (*bottom graphs*). Each graph presents the mean of two duplicate wells from a single experiment. (d) CD22sdCAR-T cells were also tested for short-term cytolytic response via chromium release assay as described in the methods section. Results show the mean of a single experiment performed in duplicate for two donors as shown, (e) graph shows the mean cytolysis at 40:1 E:T ratio from both donors +/- SEM, P-values shows the result of a student T test comparison of specific Raji killing with unmodified (mock) T cells.

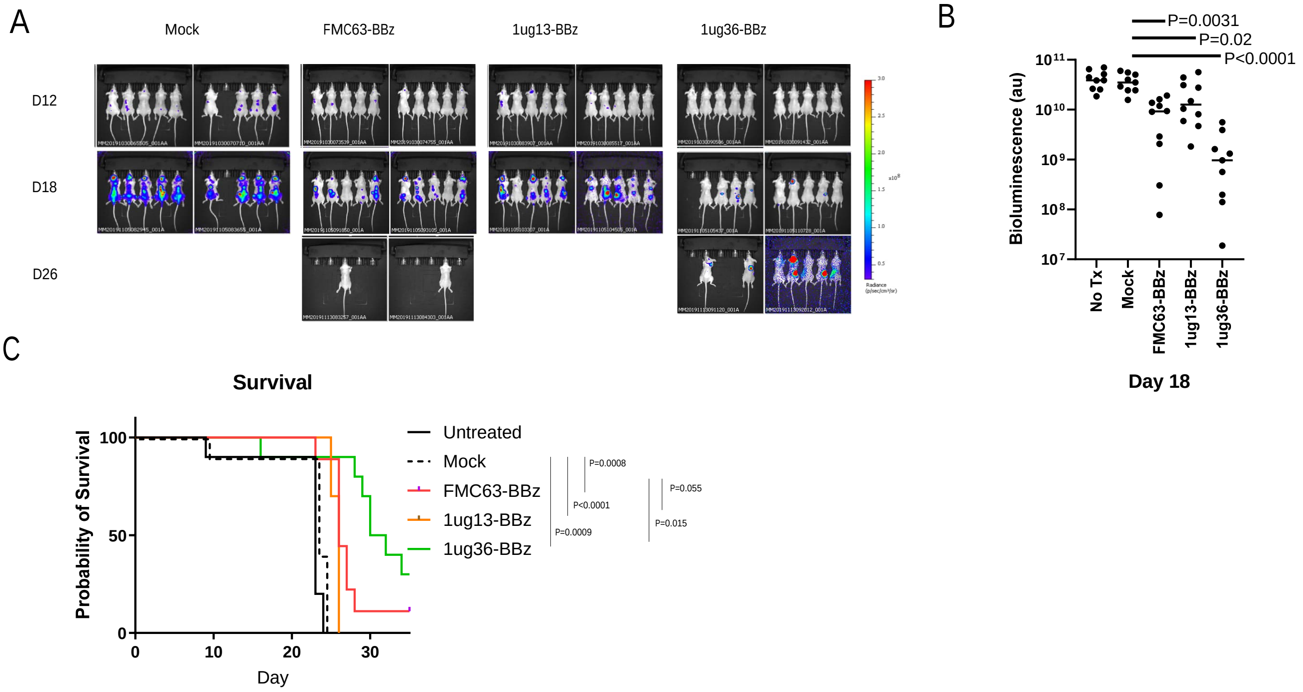

<u>Figure S6</u>: *Preliminary in vivo testing with CD22sdCAR-T cells.* Nod-SCID-IL2Rγ-null (NSG) mice were injected with 5x10^4 Ramos-FLUC cells and randomly assigned to cages, at day 3 post-tumor mice were injected with 5 x 10^6 total T cells for various CAR-T products or unmodified (mock) T cells (n=10 mice per group). Mice were monitored for by distress and euthanized at pre-determined humane endpoints. (a) Mice were assessed via IVIS imaging for bioluminescent signal from Ramos-FLUC tumours at various timepoints as shown. (b) Shows the relative bioluminescent signal at the last timepoint where control mice were surviving (day 18 post tumor injection). P values shows a student T test comparison of mock and CAR-T treatment groups. (c) The number of surviving mice at each timepoint after tumour challenge is shown in survival graphs. P values show the comparison of survival for treatment groups via Log-Rank test.

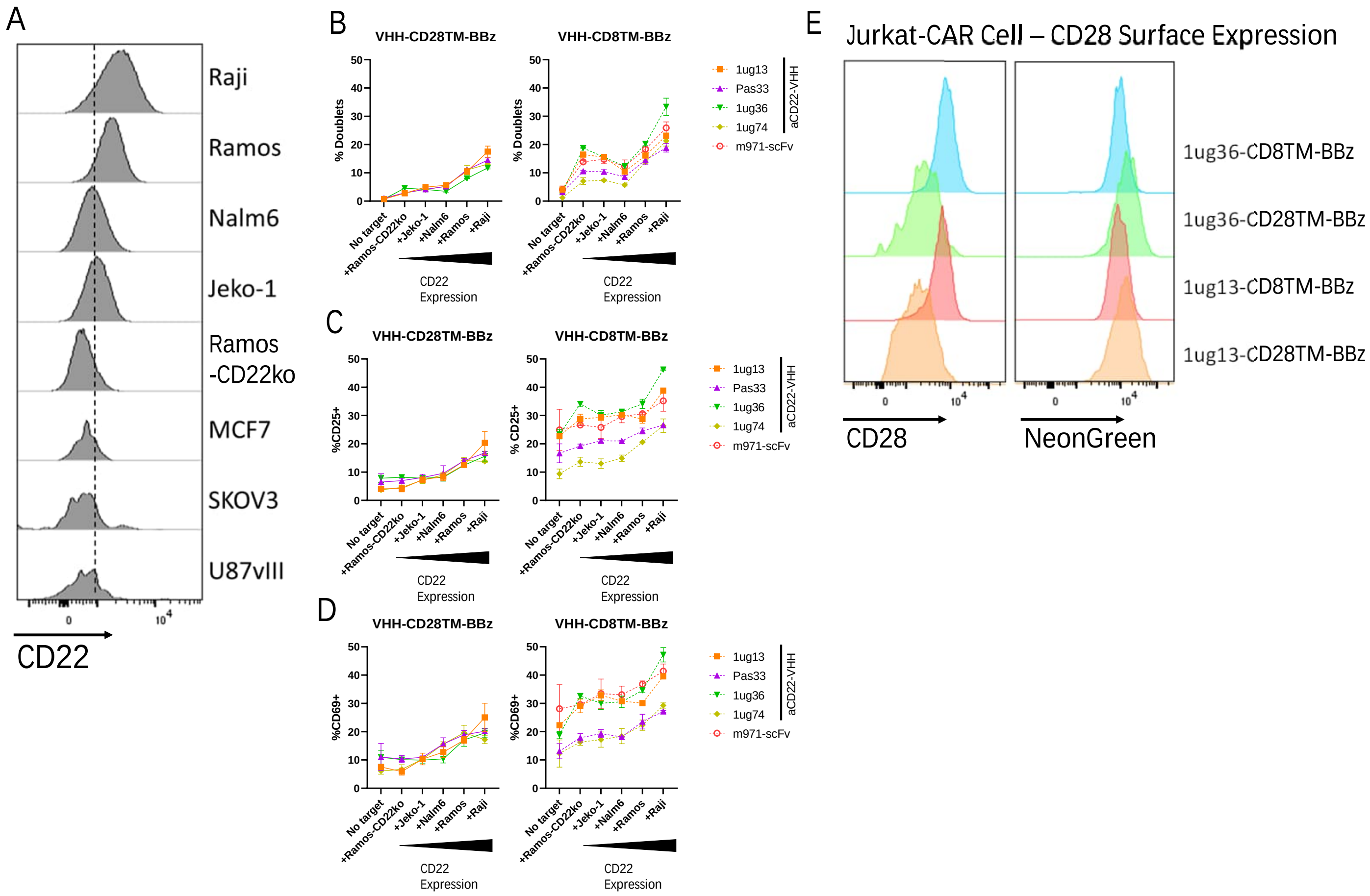

<u>Figure S7</u>: *Response of CD28TM or CD8TM CAR-T cells to various CD22 expressing cells and CD28 surface expression.* (a) Various target cell lines were examined for their relative CD22 expression using flow cytometry. (b-d) CAR-T cells were placed in overnight co-culture at high density with various target cells at an E:T ratio of 1:1. Graphs show the mean result from duplicate wells in a single experiment. (e) Jurkat cells stably expressing CD28TM or CD8TM versions of CD22sdCARs were stained with anti-human CD28 and examined via flow cytometry for CD28 expression (left) and NeonGreen expression as a marker of CAR expression (right).

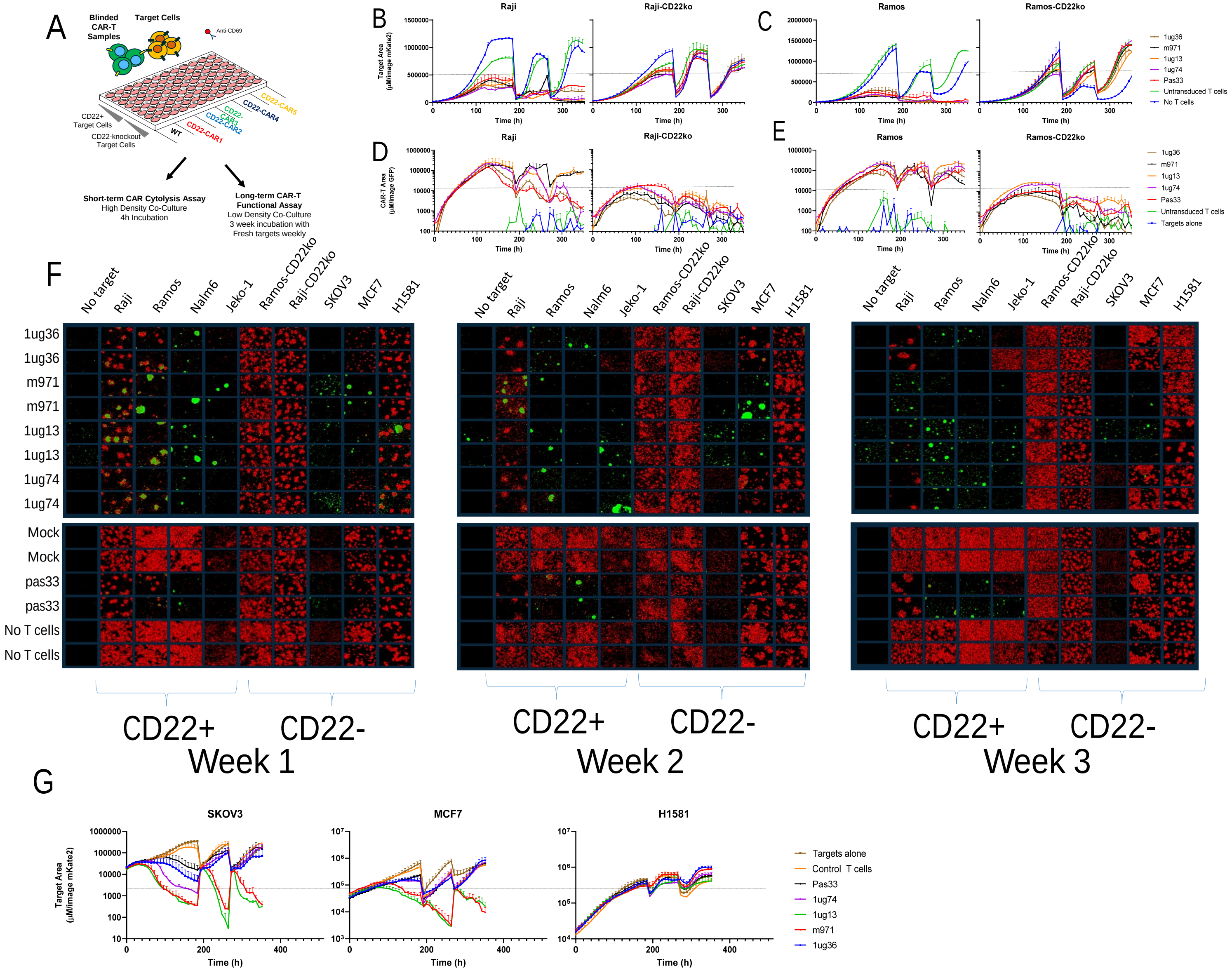

Figure S8: *Supplemental in vitro functional data for CD22-VHH CAR lead selection.* High quality CD22 CAR-T cells or untransduced control T cells were generated as described in the text. (a) CAR-T or control T cells were then assessed in blinded format via co-culture with IL7/IL15 supplementation and various target cells for functional responses using long term co-culture and live microscopy wherein cultures were split weekly with addition of fresh target cells. Co-cultures with Raji, Raji-CD22ko, Ramos, and Ramos-CD22ko cells were examined for (b-c) target cell growth or (d-e) CAR-T expansion. (f) Endpoint images of co-cultures before media exchange and addition of fresh target cells are shown at day 7, 14, and 21 [green = T cells, red = target cells] (g) Target cell growth for irrelevant CD22-negative targets is shown. (h) NSG mice implanted with Ramos-FLUC cells and treated with various CAR-T cells as per description in the text, were examined for bioluminescence at various timepoints after tumour injection as shown. P values show inter-group comparisons using student T test with log transformed bioluminescence values.

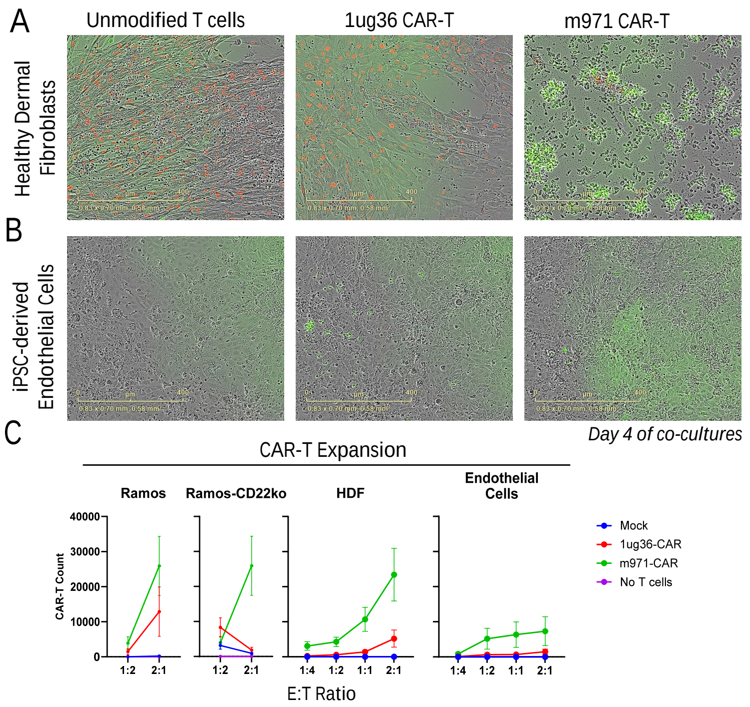

Figure S9: *Assessment of non-specific CAR-T responses against healthy donor human fibroblast and iPSC-derived endothelial cells.* 1ug36-sdAb or m971-scFv CD22 CAR-T cells or untransduced control T cells were generated as described in the text. CAR-T or control T cells were then combined at varying effector to target ratios in co-culture with human lymphoma cells (Ramos), CD22-knockout Ramos cells (Ramos-CD22ko), healthy human dermal fibroblasts (HDF), or human iPSC-derived endothelial cells. After 5 days of co-culture images were acquired to assess number of green fluorescent CAR-T or (a) red fluorescent HDF target cells. (b) Similarly, co-cultures with unmarked iPSC-derived endothelial cells were also examined. (c) After image acquisition, co-cultures were also examined for total CAR-T cell numbers per well via flow cytometry. Results show the total count of cells at endpoint averaged from 3 experiments performed in duplicate +/- SEM.

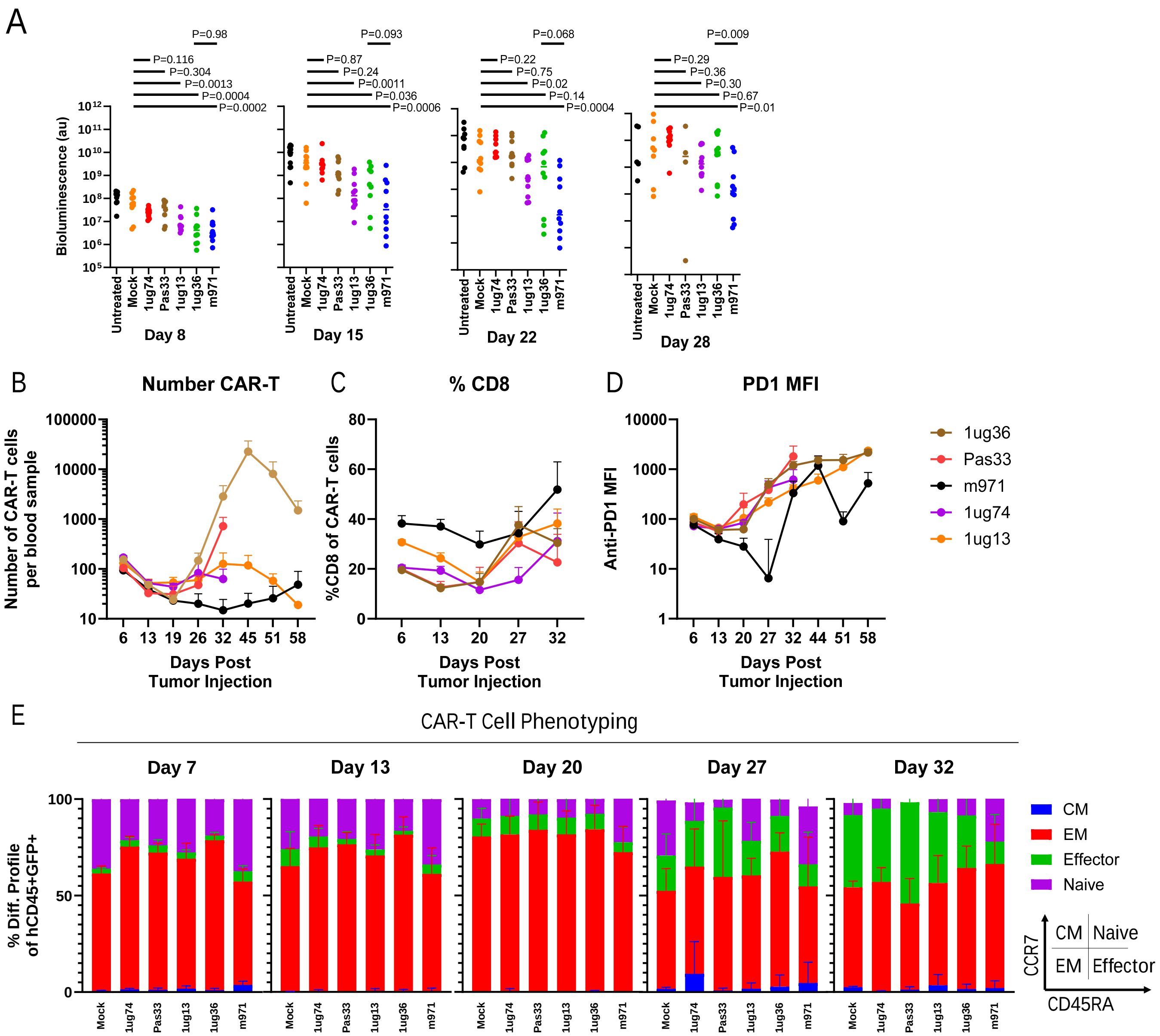

<u>Figure S10</u>: *Additional analysis of T cell phenotype for in vivo lead selection study.* The *in vivo* therapeutic activity of various CD22sdCAR-T cells were tested using Ramos xenograft model as described in the text (n=10 mice per group). Animals were bled at weekly intervals to examine circulating T cell phenotype via flow cytometry. (a) The mean number of circulating CAR-T cells (hCD45+/hCD19-/GFP+) is shown for CAR-T treatment groups. (b) The mean proportion of CD8+ cells within gated CAR-T cells is shown for each treatment group. (c) The average expression of PD1 within gated CAR-T population (median fluorescence intensity) is shown. (d) The differentiation phenotype of CAR-T cells, or total T cells for mic treated with unmodified T cells, was examined. Gating scheme is shown in inset (Central memory, CM = CCR7+/CD45RA+; Effector Memory, EM = CCR7/CD45Ra-, Effector = CCR7-/CD45RA+; Naïve = CCR7+/CD45RA+). Graphs show the mean values for all surviving mice at the timepoints as shown +/- SEM.

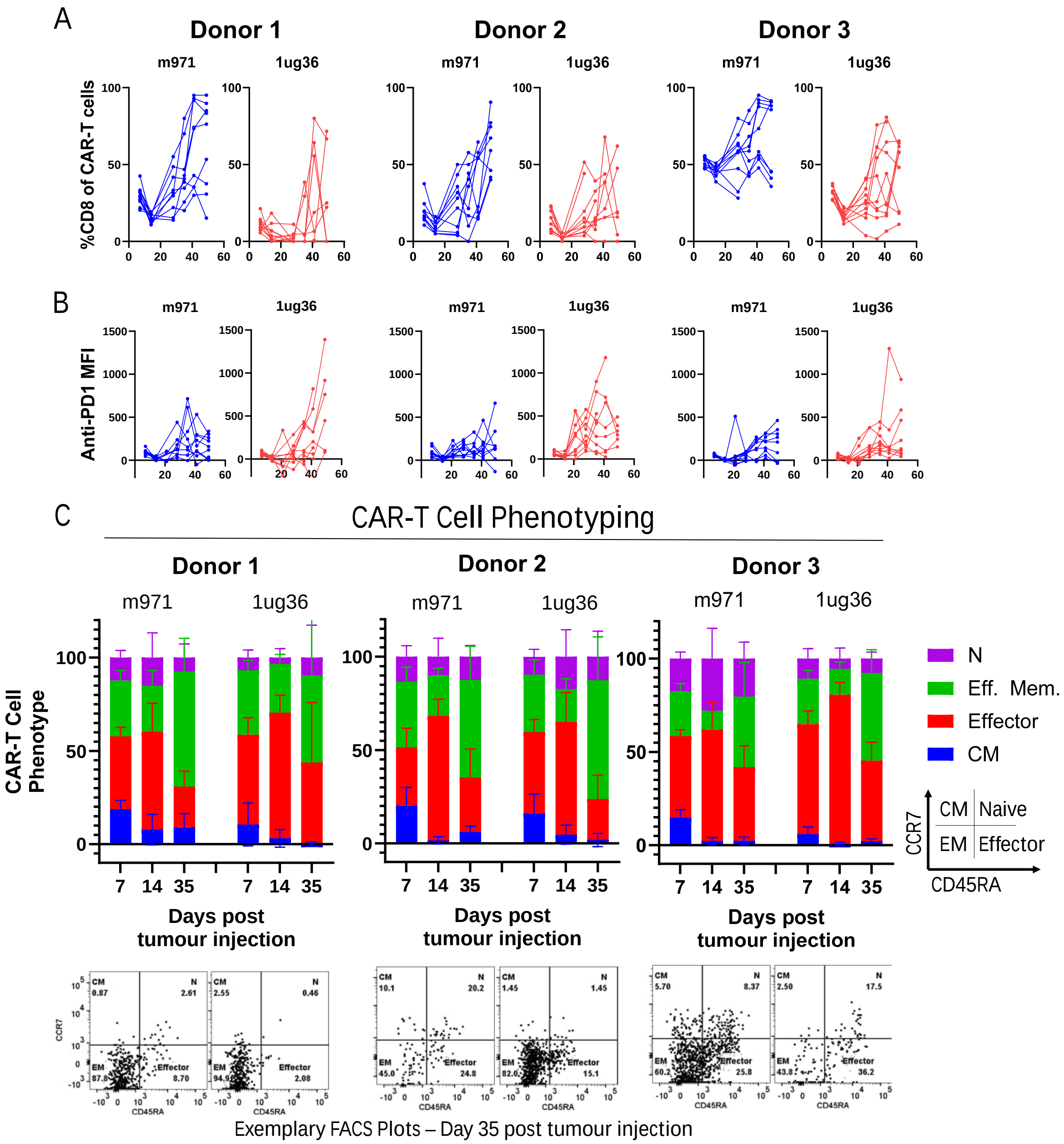

Figure S11: *Additional analysis of T cell phenotype for multi-donor in vivo lead confirmation study.* The *in vivo* therapeutic activity of 1ug36-CD22sdCAR-T or benchmark m971 CD22-scFv-CAR-T cells were tested using Ramos xenograft model as described in the text (n=10 mice per group). Animals were bled at various timepoints as shown to examine circulating T cell phenotype via flow cytometry. (a) The mean proportion of CD8+ cells within gated CAR-T cells (hCD45+/hCD19-/CD22-binding+) is shown for each treatment group. (b) The average expression of PD1 within gated CAR-T population (median fluorescence intensity) is shown. (c) The differentiation phenotype of CAR-T cells was examined. Gating scheme is shown in inset (Central memory, CM = CCR7+/CD45RA+; Effector Memory, EM = CCR7/CD45Ra-, Effector = CCR7-/CD45RA+; Naïve = CCR7+/CD45RA+). Exemplary scatterplots from day 35 post-tumour injections is shown below the bar graphs. Graphs show the mean values for all surviving mice at the timepoints as shown +/- SEM.

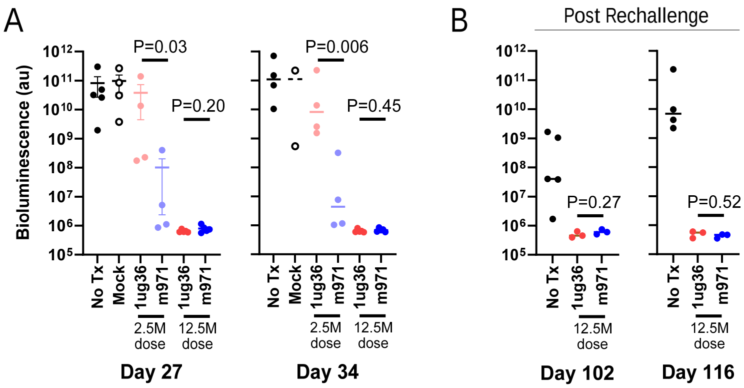

Figure S12: *Comparison of tumour load measurement via bioluminescent in vivo imaging for CD22 CAR-T dose response study*. NSG mice engrafted with 5x10^4 Ramos-FLUC cells and treated with marginal or high dose of 1ug36 or m971 CD22 CAR-T cells as per description in the main text (N=5 mice per group). (a) Mice were examined for tumour load at day 27 and 34 post tumour injection via bioluminescent imaging and the signal was quantitated per mouse. (b) Surviving high dose CAR-T mice were rechallenged with 5x10^4 Ramos-FLUC cells at day 85 post tumour injection and examined for tumour load at day 102 and 116 after initial tumour injection. P values show inter-group comparisons using student T test with log transformed bioluminescence values.

<u>Table S1</u>: *Summary of immunochemical and competitive binding characteristics for CD22-sdAbs* is provided based on the observations in surface plasmon resonance experiments performed as described in the text.

Seq. Family	sdAb Name	k_a (1/Ms)	k_d (1/s)	K_D (nM)	sdAb Used for Binning	Epitope Bin
A	1ug13	2.32E+05	6.75E-04	2.91	1ug13	Bin I
B	1ug36	1.19E+05	1.14E-03	9.59	1ug36	Bin II
C	Pas16	3.65E+05	1.00E-03	2.75	Pas16	Bin II
D	1ug77	5.50E+05	2.62E-03	4.77		Bin III
D	1ug87	1.23E+06	7.97E-04	0.65		Bin III
D	1ug75	1.40E+06	1.21E-03	0.87	1ug75	Bin III
D	1ug93	1.36E+06	1.05E-02	7.7		Bin III
E	1ug10	4.77E+05	1.76E-03	3.68	1ug10	Bin III
F	Pas79	5.14E+05	3.97E-03	7.72	Pas79	Bin III
G	1ug61	1.25E+06	6.81E-04	0.55	1ug61	Bin III
H	Pas24	1.48E+05	3.86E-03	26	Pas24	Bin IV
I	Pas32	1.58E+05	3.69E-03	23.4		Bin V
I	1ug14	3.99E+05	1.17E-05	0.03	1ug14	Bin V
J	Pas33	5.40E+04	1.40E-04	2.6		Bin VI
J	1ug74	9.25E+04	6.55E-04	7.08		Bin VI
J	1ug80	2.09E+04	6.33E-04	30.3		Bin VI
J	100ng-64	5.36E+04	1.29E-04	2.41		Bin VI
J	100ng-66	1.45E+05	1.34E-04	0.92	100ng-66	Bin VI
J	100ng-2	4.68E+04	1.30E-04	2.78		Bin VI
K	Pas64	1.08E+05	2.71E-02	250	Pas64	Bin VII

Table S2: *Summary of in vitro killing observations for CD22-CAR-T screening studies.* Target killing ranking is based on the number of red fluorescent cells observed at the end of the assay (target cells; lower is better). CAR-T expansion ranking is based on the number of green-fluorescent cells (CAR-T cells; higher is better) observed at the peak of the assay.

				Product Characteristics					*In vitro* observations									*In vivo* observations				
Screen #	CAR Design	Cell Donor	CAR-T Production Conditions	CAR ABDs Tested	Fold expansion	% CAR+	% CD4 (of CAR+)	% CD8 (of CAR+)	Figure Reference	In vitro Assay	Response Ranking - Raji		Response Ranking -		Response Ranking –		Additional	Figure References	Groups Tested	Number of mice per	Median Survival	Additional Notes
											Target Killing	CAR-T	Target Killing	CAR-T	Target Killing	CAR-T						
Screen 1	VHH-(G4S)3-CD8h-CD28TM-41BB-CD3z	Blood Donor 1<br>Female - 36	LV MOI: 10<br>Medium: StemCell ImmunoCult-XF<br>Cytokine: IL-2<br>Fresh or Frozen: Fresh<br>Harvest day: 10	Untransduced<br>FMC63-scFv<br>1ug13<br>1ug61<br>1ug36<br>1ug10	291.8<br>223<br>361.5<br>351<br>318.5<br>142.8	0<br>3.78<br>27.8<br>27.8<br>28.5<br>32.6	33.8<br>27.7<br>18.8<br>19.4<br>22.7<br>19.2	33.1<br>62<br>75.8<br>75.1<br>70.7<br>74.2	Figure 3A<br>Supp. Figure 4	5:1 E:T with IL2	FMC63*<br>1ug13*<br>1ug10*<br>1ug61*	1ug10<br>FMC63*<br>1ug13*<br>1ug61*	1ug10<br>1ug13<br>FMC63<br>1ug61	1ug10<br>1ug36<br>1ug13<br>FMC63	1ug36<br>1ug10*<br>1ug61*<br>1ug13	Minimal for all		Supp. Figure 5	No treatment<br>Untransduced<br>FMC63-scFv<br>1ug13<br>1ug36	10<br>10<br>10<br>10<br>10	23<br>23<br>26<br>26<br>31	
Screen 2	VHH-(G4S)3-CD8h-CD28TM-41BB-CD3z	Blood Donor 2<br>Male - 25	LV MOI: 10<br>Medium: StemCell ImmunoCult-XF<br>Cytokine: IL-2<br>Fresh or Frozen: Fresh<br>Harvest day: 9	Untransduced<br>1ug61<br>1ug36<br>1ug10<br>1ug74<br>1ug14<br>1ug6<br>Pas33	614<br>448<br>646<br>470<br>485<br>390<br>363<br>597	0<br>19.1<br>31.3<br>23.3<br>26.7<br>14.6<br>22.6<br>26.8	48.6<br>44.2<br>40.8<br>39.4<br>43.2<br>39.4<br>37.7<br>37.7	45.2<br>45.6<br>50.2<br>54.4<br>49.1<br>52<br>52.8<br>56.5	Figure 3B	5:1 E:T with IL2	1ug36<br>1ug14<br>1ug74<br>1ug10<br>1ug6<br>1ug61<br>Pas33	1ug36<br>1ug10<br>1ug14<br>1ug6<br>1ug74<br>Pas33*<br>1ug61*	Pas33<br>1ug36*<br>1ug74*<br>1ug14<br>1ug10*<br>1ug6*<br>1ug61	1ug36<br>Pas33<br>1ug6<br>1ug74<br>1ug6<br>1ug10<br>1ug61	1ug36<br>Pas33<br>1ug14<br>1ug61<br>1ug10<br>1ug6<br>1ug74	Minimal for		Not done				
Screen 3	VHH-(G4S)3-CD8h-CD28TM-41BB-CD3z	Blood Donor 3<br>Female- 41	LV MOI: 10<br>Medium: StemCell ImmunoCult-XF<br>Cytokine: IL-7/IL-15<br>Fresh or Frozen: Fresh<br>Harvest day: 9	Untransduced<br>m971-scFv<br>1ug13<br>1ug36<br>1ug74<br>1ug6<br>Pas33	13<br>86.7<br>86<br>66<br>92<br>74<br>100	0<br>60.3<br>87.6<br>90.6<br>90.8<br>89<br>89.9	30.4<br>58.5<br>28.4<br>35.6<br>43.2<br>39.9<br>41.3	61.4<br>36.8<br>57.7<br>59.2<br>52.5<br>52.6<br>54.6	Figure 3C-E	25:1 E:T with IL7/15	1ug36<br>m971<br>1ug74<br>1ug13*<br>Pas33*<br>1ug6*	1ug74<br>m971<br>Pas33<br>1ug36<br>1ug13*<br>1ug6*	1ug36<br>m971<br>Pas33*<br>1ug74*<br>1ug6*<br>1ug13*	m971<br>1ug74<br>1ug36<br>Pas33<br>1ug13<br>1ug6	Minimal for all	Minimal for all		Not done				
Screen 3	VHH-(G4S)3-CD8h-CD28TM-41BB-CD3z	Blood Donor 4<br>Male - 33	LV MOI: 10<br>Medium: StemCell ImmunoCult-XF<br>Cytokine: IL-7/IL-15<br>Fresh or Frozen: Fresh<br>Harvest day: 9	Untransduced<br>m971-scFv<br>1ug13<br>1ug36<br>1ug74<br>1ug6<br>Pas33	50<br>60<br>114<br>100<br>130<br>20<br>158	0<br>39<br>81.8<br>82.5<br>85.1<br>83.1<br>85.5	19.1<br>39.9<br>21.4<br>23.2<br>21.4<br>21.1<br>22.8	73<br>55.8<br>73.1<br>72.7<br>72.2<br>72.4<br>68.9	Figure 3C-E	25:1 E:T with IL7/15	1ug74*<br>Pas33*<br>1ug13*<br>1ug36*<br>m971*<br>1ug6	1ug74<br>m971<br>Pas33<br>1ug36<br>1ug13*<br>1ug6*	1ug36<br>m971<br>Pas33*<br>1ug74*<br>1ug6*<br>1ug13	1ug13<br>M971*<br>1ug74*<br>1ug36*<br>Pas33*<br>1ug6*	Minimal for all	Minimal for all		Not done				
Screen 4	VHH-(G4S)3-CD8h-CD28TM-41BB-CD3z	Blood Donor 5<br>Male - 29	LV MOI: 1<br>Medium: Miltenyi TexMACs<br>Cytokine: IL-7/IL-15<br>Fresh or Frozen: Fresh<br>Harvest day: 14	1ug13<br>1ug36<br>1ug74<br>Pas33	245<br>224<br>194<br>213	52.5<br>62.7<br>58.8<br>62.4	40.1<br>41.1<br>26.8<br>33.5	27.8<br>25<br>40.1<br>37.3	Figure 4	25:1 E:T with IL7/15	Pas33<br>1ug74<br>1ug13*<br>1ug36*	Pas33<br>1ug74*<br>1ug13*<br>1ug36	1ug13<br>1ug74<br>1ug36<br>Pas33	1ug13<br>1ug74<br>Pas33<br>1ug36	Minimal for all	Minimal for all		Not done				
	VHH-CD8h-CD8TM-41BB-CD3z			Untransduced<br>m971-scFv<br>1ug13<br>1ug36<br>1ug74<br>Pas33	168<br>73.8<br>156<br>125.6<br>184<br>176	0<br>32<br>40.4<br>28.4<br>39.3<br>49.7	29.2<br>28<br>25.6<br>28.4<br>23.5<br>27	51.2<br>34.2<br>49.4<br>49.9<br>50.2<br>52		25:1 E:T with IL7/15	Pas33<br>1ug74<br>1ug13<br>1ug36<br>m971	Pas33<br>1ug74<br>1ug13<br>m971<br>1ug36	m971<br>1ug36<br>1ug74*<br>1ug13*<br>Pas33	Pas33<br>1ug13<br>1ug74<br>m971<br>1ug36	1ug13<br>Pas33<br>Minimal	1ug13<br>Pas33<br>m971<br>1ug74<br>1ug36	Much higher tonic expansion than CD28TM constructs	Figure 5	No treatment<br>Untransduced<br>m971 - 2.5M<br>m971 - 12.5M<br>1ug36 - 2.5M<br>1ug36 - 12.5M	5<br>5<br>5<br>5<br>5<br>5	34<br>27<br>58<br>>116<br>47<br>>116	Variable Dose Testing<br>Mice rechallenegd at day 85
Screen 5<br>Lead Selection Study	VHH-CD8h-CD8TM-41BB-CD3z	Leukopak donor 0<br>Male-42 years old	LV MOI: 10<br>Medium: Miltenyi TexMACs<br>Cytokine: IL-7/IL-15<br>Fresh or Frozen: Frozen<br>Harvest day: 14	Untransduced<br>m971-scFv<br>1ug13<br>1ug36<br>1ug74<br>Pas33	217<br>144<br>121<br>193<br>161<br>166	0.2<br>49.14<br>55.7<br>32.1<br>56.3<br>61.7	75.5<br>18.1<br>50.3<br>53.7<br>23.2<br>32.9	16.3<br>73.5<br>41.1<br>38.5<br>61.4<br>39.1	Supp. Figure 6<br>Figure 5	25:1 E:T with IL7/15	m971<br>1ug13<br>1ug74<br>1ug36<br>Pas33	m971<br>1ug13<br>1ug74<br>1ug36<br>Pas33	All similar	1ug13<br>Pas33<br>1ug74<br>1ug36<br>m971	Minimal for all	1ug13<br>1ug74<br>Pas33<br>m971<br>1ug36		Figure 4	No treatment<br>Untransduced<br>m971-scFv<br>1ug74<br>Pas33<br>1ug13<br>1ug36	10<br>10<br>10<br>10<br>10<br>10<br>10	29<br>29<br>>60<br>33.5<br>28<br>42<br>>60	
Screen 6<br>Multi-donor activity confirmation study	VHH-CD8h-CD8TM-41BB-CD3z	Leukopak donor 1<br>Female-22 years old	LV MOI: 10<br>Medium: Miltenyi TexMACs<br>Cytokine: IL-7/IL-15<br>Fresh or Frozen: Frozen<br>Harvest day: 14	Untransduced<br>m971-scFv<br>1ug36	704<br>592<br>560	0<br>28<br>30	59.1<br>25<br>90.3	40.9<br>75<br>9.7	Not done									Supp. Figure 8	Untreated<br>Untransduced<br>m971-scFv<br>1ug36	10<br>10<br>10<br>10	28<br>29<br>114<br>46	Mice rechallenegd at day 85
																			Untreated<br>Untransduced<br>m971-scFv<br>1ug36	10<br>10<br>10<br>10	21<br>24<br>>90<br>>90	Donor 1 Repeat<br>Mice rechallenegd at day 85
		Leukopak donor 2<br>Male-24 years old		Untransduced<br>m971-scFv<br>1ug36	848<br>624<br>896	0<br>22<br>33	77.8<br>89.8<br>93.7	22.2<br>10.2<br>6.3											Untransduced<br>m971-scFv<br>1ug36	10<br>10<br>10	32<br>92<br>52	Mice rechallenegd at day 85
		Leukopak donor 3<br>Male-28 years old		Untransduced<br>m971-scFv<br>1ug36	664<br>440<br>944	0<br>25<br>38	37.5<br>61.9<br>75	62.5<br>38.1<br>25											Untransduced<br>m971-scFv<br>1ug36	10<br>10<br>10	32<br>88<br>105	Mice rechallenegd at day 85

nd: Not done; ABD: antigen binding domain;

Table S3: *Summary of observations in the human tissue array stained with CD22 1ug36-VHH or irrelevant specificity B131-VHH*. Staining was performed with anti-VHH secondary and anti-mouse tertiary as described in the methods section. Observers 1 and 2 were blinded to sample identity in stained slides and recorded their observations independently. Representative images are provided for anti-CD22 sdAb 1ug36 stained slide (slide 1) and irrelevant specificity sdAb B131 stained slide (slide 2).

See supplemental excel document available online.

Table S4: *Flow Cytometry Antibody Staining Panel used for mouse blood analysis.* A complete list of the staining panel used for analysis of circulating T cells in the in vivo lead selection and multi-donor lead confirmation studies is provide, including supplier information.

Lasers	Filters	Marker	Clone	Fluorochrome	Catalogue #	Lot #	Supplier	Storage conditions
Violet Laser (405 nm)	780/60	CD69	FN50	BV786	563834	276145	BD Biosciences	4°C
	710/50	mCD45	30F11	BV711	563709	1076257	BD Biosciences	4°C
	660/20	CD45RA	HI100	BV650	563963	1036332	BD Biosciences	4°C
	610/20	CD45RO	UCHL1	BV605	562791	1039402	BD Biosciences	4°C
	525/50	N/A	N/A	N/A	N/A	N/A	N/A	N/A
	450/40	CD279 (PD1)	EH12.1	BV421	329920	B314841	BioLegend	4°C
Blue laser (488nm)	710/50	CD8	SK1	PerCP-Cy5.5	565310	135209	BD Biosciences	4°C
	530/30	hSiglec-2 CD22-Protein-His-Tag	N/A	FITC	SI2-HF2H6-25ug	IAF2028b	Acro Biosystems (Distributor: Cedarlane, Canada)	4°C
Yellow-Green Laser (561nm)	780/60	CD25	M-A251	PE-Cy7	557741	1068706	BD Biosciences	4°C
	710/50	N/A	N/A	N/A	N/A	N/A	N/A	N/A
	610/20	N/A	N/A	N/A	N/A	N/A	N/A	N/A
	586/15	CD197 (CCR7)	3D12	PE	552176	184671	BD Biosciences	4°C
Red Laser (640nm)	780/60	hCD45	2D1	APC-H7	560178	274948	BD Biosciences	4°C
	730/45	N/A	N/A	N/A	N/A	N/A	N/A	N/A
	670/14	CD22	S-HCL-1	APC	363506	B290368	BioLegend	4°C
UV Laser (355nm)	740/35	CD27	L128	BUV737	564301	1112895	BD Biosciences	4°C
	515/30	CD19	SJ25C1	BUV496	612938	233664	BD Biosciences	4°C
	379/28	CD4	SK3	BUV395	563550	9273692	BD Biosciences	4°C
