# Supplementary material for: Discovery and preclinical development of a therapeutically active nanobody-based chimeric antigen receptor targeting human CD22
Source: Mol Ther Oncol. 2024 Feb 13;32(1):200775. doi: 10.1016/j.omton.2024.200775 (PMC10914482; doi:10.1016/j.omton.2024.200775)
Supplement: Document S2. Article plus supplemental information [file mmc3.pdf]

Molecular Therapy
Oncology
Original Article

Discovery and preclinical development of a therapeutically active nanobody-based chimeric antigen receptor targeting human CD22

Scott McComb,[1,2,3] Mehdi Arbabi-Ghahroudi,[1,2] Kevin A. Hay,[4,5] Brian A. Keller,[6,8] Sharlene Faulkes,[16] Michael Rutherford,[6,7] Tina Nguyen,[1] Alex Shepherd,[1,2] Cunle Wu,[1,12] Anne Marcil,[1] Annie Aubry,[1] Greg Hussack,[1] Devanand M. Pinto,[1] Shannon Ryan,[1] Shalini Raphael,[1] Henk van Faassen,[1] Ahmed Zafer,[1] Qin Zhu,[1] Susanne Maclean,[1] Anindita Chattopadhyay,[1] Komal Gurnani,[1] Rénald Gilbert,[1] Christine Gadoury,[1] Umar Iqbal,[1] Dorothy Fatehi,[1] Anna Jezierski,[1,2] Jez Huang,[1] Robert A. Pon,[1] Mhairi Sigrist,[4] Robert A. Holt,[13,14,15] Brad H. Nelson,[11,13] Harold Atkins,[9] Natasha Kekre,[9,10] Eric Yung,[14] John Webb,[11] Julie S. Nielsen,[11] and Risini D. Weeratna[1]

[1]Human Health Therapeutics Research Centre, National Research Council, Ottawa, ON, Canada; [2]Department of Biochemistry, Microbiology, and Immunology, Faculty of Medicine, University of Ottawa, Ottawa, ON, Canada; [3]Centre for Infection, Immunity, and Inflammation, University of Ottawa, Ottawa, ON, Canada; [4]Terry Fox Laboratory, British Columbia Cancer Research Institute, Vancouver, BC, Canada; [5]Division of Hematology, Faculty of Medicine, University of British Columbia, Vancouver, BC, Canada; [6]Division of Anatomical Pathology, The Ottawa Hospital/University of Ottawa, Ottawa, ON, Canada; [7]Division of Hematopathology and Transfusion Medicine, The Ottawa Hospital/University of Ottawa, Ottawa, ON, Canada; [8]University of Ottawa Faculty of Medicine, Ottawa, ON, Canada; [9]Division of Hematology, Department of Medicine, The Ottawa Hospital, Ottawa, ON, Canada; [10]Clinical Epidemiology Program, Ottawa Hospital Research Institute, Ottawa, ON, Canada; [11]Deeley Research Centre, British Columbia Cancer Research Institute, Victoria, BC, Canada; [12]Department of Biology, Concordia University, Montréal, QC, Canada; [13]Department of Medical Genetics, University of British Columbia, Vancouver, BC, Canada; [14]Canada's Michael Smith Genome Sciences Centre, Vancouver, BC, Canada; [15]Department of Molecular Biology & Biochemistry, Simon Fraser University, Burnaby, BC, Canada; [16]Department of Pathology and Laboratory Medicine, Faculty of Medicine, University of Ottawa, Ottawa, ON, Canada

Chimeric antigen receptor (CAR) T cell therapies targeting B cell-restricted antigens CD19, CD20, or CD22 can produce potent clinical responses for some B cell malignancies, but relapse remains common. Camelid single-domain antibodies (sdAbs or nanobodies) are smaller, simpler, and easier to recombine than single-chain variable fragments (scFvs) used in most CARs, but fewer sdAb-CARs have been reported. Thus, we sought to identify a therapeutically active sdAb-CAR targeting human CD22. Immunization of an adult *Llama glama* with CD22 protein, sdAb-cDNA library construction, and phage panning yielded >20 sdAbs with diverse epitope and binding properties. Expressing CD22-sdAb-CAR in Jurkat cells drove varying CD22-specific reactivity not correlated with antibody affinity. Changing CD28- to CD8-transmembrane design increased CAR persistence and expression *in vitro*. CD22-sdAb-CAR candidates showed similar CD22-dependent CAR-T expansion *in vitro*, although only membrane-proximal epitope targeting CD22-sdAb-CARs activated direct cytolytic killing and extended survival in a lymphoma xenograft model. Based on enhanced survival in blinded xenograft studies, a lead CD22sdCAR-T was selected, achieving comparable complete responses to a benchmark short linker m971-scFv CAR-T in high-dose experiments. Finally, immunohistochemistry and flow cytometry confirm tissue and cellular-level specificity of the lead CD22-sdAb. This presents a complete report on preclinical development of a novel CD22sdCAR therapeutic.

INTRODUCTION

Chimeric antigen receptor T cell (CAR-T) therapy is a complex cell therapy in which a patient's own immune cells are genetically modified to redirect their activity toward cancer-associated surface antigens. Currently, there are several CAR-T therapies targeting CD19 for the treatment of B cell leukemia and lymphoma, and B cell maturation antigen (BCMA) for the treatment of multiple myeloma, at least 5 of which have received regulatory approval for commercial treatment in numerous countries as of mid-2023. CD19 CAR-T therapy has led to durable complete responses for patients with few other treatment options.[1] Despite this success, many patients will relapse after CD19 CAR-T therapy, with loss of CD19 antigen expression accounting for 10%–30% of relapses, although this varies by disease

Received 29 September 2023; accepted 9 February 2024;
https://doi.org/10.1016/j.omton.2024.200775.

Correspondence: Scott McComb, Human Health Therapeutics Research Centre, National Research Council, Canada.
E-mail: scott.mccomb@nrc-cnrc.gc.ca

This is an open access article under the CC BY license (http://creativecommons.org/licenses/by/4.0/).

indication.[2–4] Similar to CD19, other B cell-restricted antigens such as CD22 and CD20 can be effective targets for CAR-T therapy and have generally shown efficacy and tolerability profiles similar to those of CD19-directed therapy in clinical trials reported to date.[5–9]

Although most CAR-T therapies use human or mouse antibodies reformatted into single-chain variable fragments (scFvs) for antigen targeting, CARs can also be constructed using single-domain antibody (sdAb) fragments, which are derived from natural heavy-chain-only antibodies found in various species of camelids (llamas and camels) and sharks.[10] Originally named the variable heavy domain of camelid heavy-chain only antibodies (VHH) or sdAbs, and often referred to as nanobodies; camelid-derived sdAbs are approximately half the size of an scFv and have higher homology to human VH3 family variable domains than mouse antibodies, potentially improving the immunogenic profile of sdAb-based CAR-T products.[11,12] Due to their monomeric nature, sdAbs can more easily be recombined in molecules such as CARs, or larger multiantigen targeted CAR receptors. The most clinically advanced sdAb-based CAR-T product is a BCMA-targeted therapy, ciltacabtagene autoleucel, which combines two BCMA-specific sdAb elements and received US Food and Drug Administration approval for the treatment of multiple myeloma in 2022. In indirect comparison, this sdAb-based CAR-T product appears to have better response rates than a similar scFv-based CAR-T targeting BCMA,[13] with a typical toxicity profile similar to other CAR-T therapies and no reported issues related to immunogenicity. Additional preclinical and clinical development of high-quality sdAb-CAR therapies will be needed to provide insight as to whether sdAb-based CARs can offer generalized benefit over scFv-based single- and multiantigen targeted CAR therapeutics.

Here, we report the isolation of novel llama sdAbs with a range of activity in redirecting T cell responses toward CD22-expressing target cells. CD22 is a B cell-restricted member of the Siglec family of surface receptors; it has a complex regulatory function in B cell receptor signaling, and is a proven immunotherapy target for B cell malignancies.[6,14,15] By immunizing an adult *Lama glama* with the extracellular domain (ECD) of human CD22, we were able to isolate a large number of unique sdAb binders with varying domain specificity and binding properties. Through a series of selective experiments, we identify a specific membrane-proximal epitope targeting the CD22sdCAR molecule, which showed therapeutic activity in an *in vivo* xenograft CAR-T treatment model of human lymphoma, with comparable activity to a benchmark short linker m971-scFv CAR when administered at higher doses.[7] We also present data demonstrating cell- and tissue-level specificity of our lead CD22sdAb molecule, with no unexpected tissue binding in nonlymphoid tissues. Overall, these data report the nonclinical pharmacology studies supporting the clinical development of a CD22-targeted sdAb-based CAR-T therapeutic.

RESULTS

Isolation of an anti-CD22-sdAbs

Human CD22 is a type I single-pass transmembrane protein with a large ECD, composed of 7 immunoglobulin-like subdomains (Figure 1A). To produce new sdAbs targeting human CD22, we first generated purified recombinant human CD22-ECD (amino acids [aa]1–668), as previously described.[16] Purified protein was characterized by chromatography (size exclusion chromatography-ultrahigh performance liquid chromatography [SEC-UPLC]) to confirm the expected product size and lack of aggregation. An adult male llama was then immunized with CD22-ECD protein, and the immune response was confirmed using a CD22 protein ELISA, comparing preimmune serum to serum drawn postimmunization (day 49), in which a strong CD22-specific heavy-chain antibody response was detected by ELISA (Figure 1B). The presence of such CD22-reactive heavy-chain antibodies is strongly indicative that clonal sdAbs specific for human CD22 should be found within the immunized llama.

A phagemid library was constructed using the amplified total heavy-chain variable domain repertoire extracted from llama peripheral blood mononuclear cells (PBMCs) isolated at day 49 after the first injection, as previously described.[17] Two panning strategies were used to isolate a total of 27 unique sdAbs with strong reactivity to human CD22 protein. Identified anti-CD22 sdAbs were then expressed in *Escherichia coli*, purified by an immobilized metal affinity column, and biotinylated at their C termini using an *in vivo* biotinylating system, as previously reported.[18] From the initial panel of 27 sdAbs, 20 showed acceptable production yield with nonaggregating and monomeric behaviors as determined by SEC, and thus were tested further.

Specific binding of sdAbs to human CD22-ECD was confirmed by ELISA and surface plasmon resonance (SPR) analysis using CD22-ECD immobilized onto a CM5 dextran chip. Analysis of the SPR kinetic data with 1:1 binding model fit revealed that monomeric sdAbs had equilibrium dissociation constants (K_D; binding affinity) ranging between 0.03 and 250 nM, with a median value of 3.6 nM (Figures 1C, 1D, and S1; Table S1). Based on sdAb sequence similarity, the 20 anti-CD22 sdAbs represented 11 sequence families. A representative VHH from each sequence family was then tested for competitive binding via co-injection SPR experiments (Figures 1E and S1; Table S1), revealing 7 noncompetitive epitope bins in the panel of anti-CD22 sdAbs. Overall, these results identify a diverse set of sdAbs that bind human CD22 with a range of affinities and epitope specificities.

To map noncompetitive sdAbs to CD22 domain specificity, we next examined binding using a yeast surface-display strategy, in which CD22-ECDs were individually expressed in yeast and probed with CD22-VHH proteins from each family of binders via whole-cell ELISA. We found that sdAbs recognized CD22 domains 1, 4, 6, and 7, numbered from N to C terminus (Figures 1F and 1G). We also confirmed cell-binding activity for a subset of sdAbs, which were probed against CD22-expressing cells (Raji or Ramos) or clonal CD22-knockout (KO) cells generated using CRISPR plasmid electroporation (Ramos-CD22KO; Figure S2). Overall, these results reveal a diverse set of CD22-specific sdAbs with varying affinity, domain targeting, and cell-binding properties.

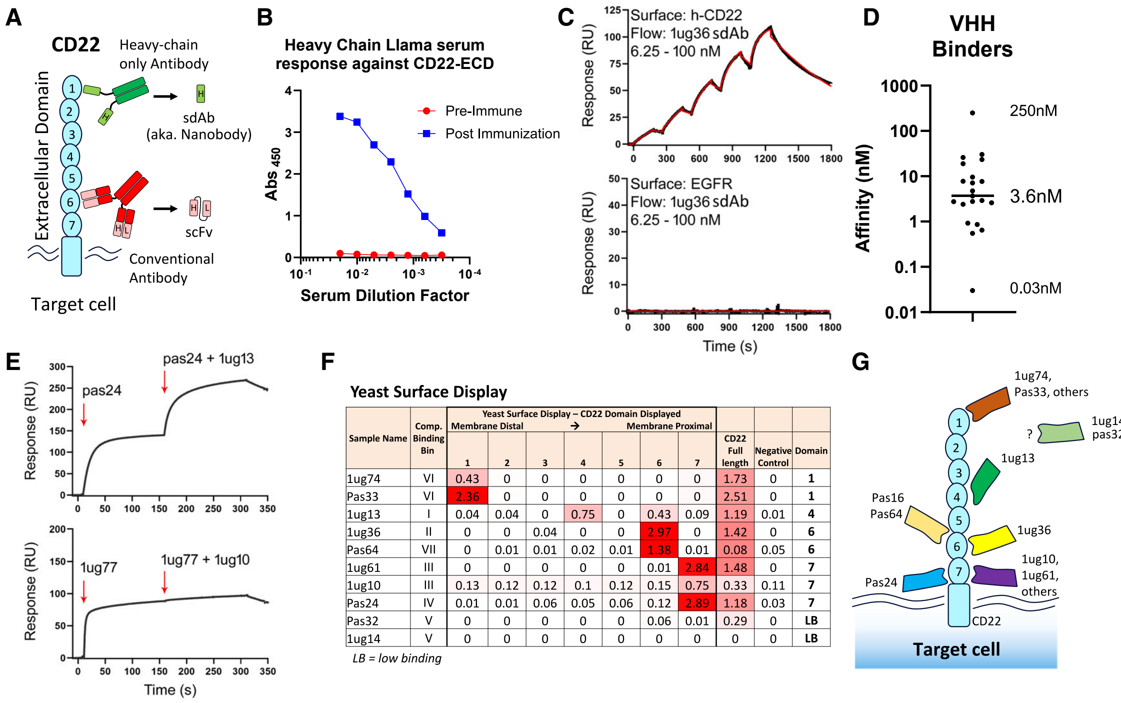

Figure 1. Identification of novel CD22-targeting nanobodies with varying affinity and epitope specificity

(A) Structure of CD22 protein, including 7 large Ig-like ECDs. Antibody structures are also shown for either a camelid heavy-chain-only antibody (top) or conventional heavy-/light-chain antibody (bottom). (B) CD22-specific serum response in a llama was measured via ELISA at preimmunization and after CD22-ECD immunization and boosting. (C) Representative SPR sensorgrams showing specific sdAb binding to immobilized CD22-ECD. (D) Distribution of 20 anti-CD22 sdAb equilibrium K_Ds. (E) Representative SPR co-injection sensorgrams from epitope binning experiments showing a pair of sdAbs binding distinct CD22 epitopes (top) and a pair of sdAbs binding an overlapping CD22 epitope (bottom). (F) YSD of specific CD22 domains was performed to map binding of sdAbs. (G) A summary of the CD22 domain-specific binding for sdAbs.

High-throughput CAR activity screening of CD22-sdAb in Jurkat T cells

Next, anti-CD22 sdAb sequences were transferred via PCR amplification from the phagemid vector into a modular CAR plasmid backbone via scarless cloning, as previously described.[19] The modular CAR design used for high-throughput cloning had a structure as follows: sdAb, flexible linker domain, CD8-hinge domain, CD28-transmembrane (TM) domain, 41BB co-stimulatory domain, and a CD3-ζsignaling domain (Figure 2A). After attempting to clone all 27 CD22 sdAbs into our modular CAR vector, a total of 22 constructs were confirmed to be correctly assembled via Sanger sequencing and moved to functional screening via a high-throughput CAR-Jurkat assay. The results revealed a range of CAR-Jurkat reactivity to CD22$^+$ Ramos cells, with approximately half of the constructs inducing a clear upregulation of CD69 expression on CAR-Jurkat cells, a marker of T cell activation (Figure 2B). None of the constructs showed responses to clonal CD22-KO Ramos target cells generated using CRISPR plasmids (Figure S2B).

For comparison, we also tested a number of novel CD22 scFv CARs derived from in-house generated anti-human CD22 mouse monoclonal antibodies, which we confirmed to bind with varying apparent affinity and domain specificity to CD22$^+$ Ramos cells (Figures 2B and S3). In addition, we tested a benchmark scFv CAR targeting CD22 (short linker m971),[7,20] or an scFv CAR targeting CD19 (FMC63) (Figure 2B, right). As expected, all of the scFv-based CARs tested produced antigen-specific responses. CD22 specificity was also confirmed using an alternate human lymphoma line, Raji or Raji-CD22-KO target cells (Figure 2C), and with CD22-overexpressing K562 or wild-type (WT) K562 cells, which do not express CD22 (Figure 2D). No expression of CD69 was observed following coculture with an irrelevant target, CD22$^-$ SKOV3 human ovarian cancer cells (Figure 2D).

Across all of the CAR-Jurkat assessments, we found that the magnitude of the CD22-specific activation response in CD22sdCAR-expressing Jurkat cells did not correlate with antibody on-rates, off-rates, or affinity (Figure 2E). Only 2 out of 9 CD22sdCAR constructs targeting the most highly membrane-adjacent domain 7 of CD22 showed CAR functionality, likely due to poor epitope accessibility, because domain 7 binding sdAb proteins also showed relatively weak cell binding in the purified sdAb format (Figure S2C) and induced poor CAR-target doublet formation (Figure 2F). In contrast to directly membrane-adjacent epitope targeting sdAb-CARs, the majority of CD22sdCARs targeting domains 1, 4, or 6 showed CD22 responsiveness (Figure 2D), correlating with higher cell binding (Figure S2C) and higher CAR-target cellular avidity,

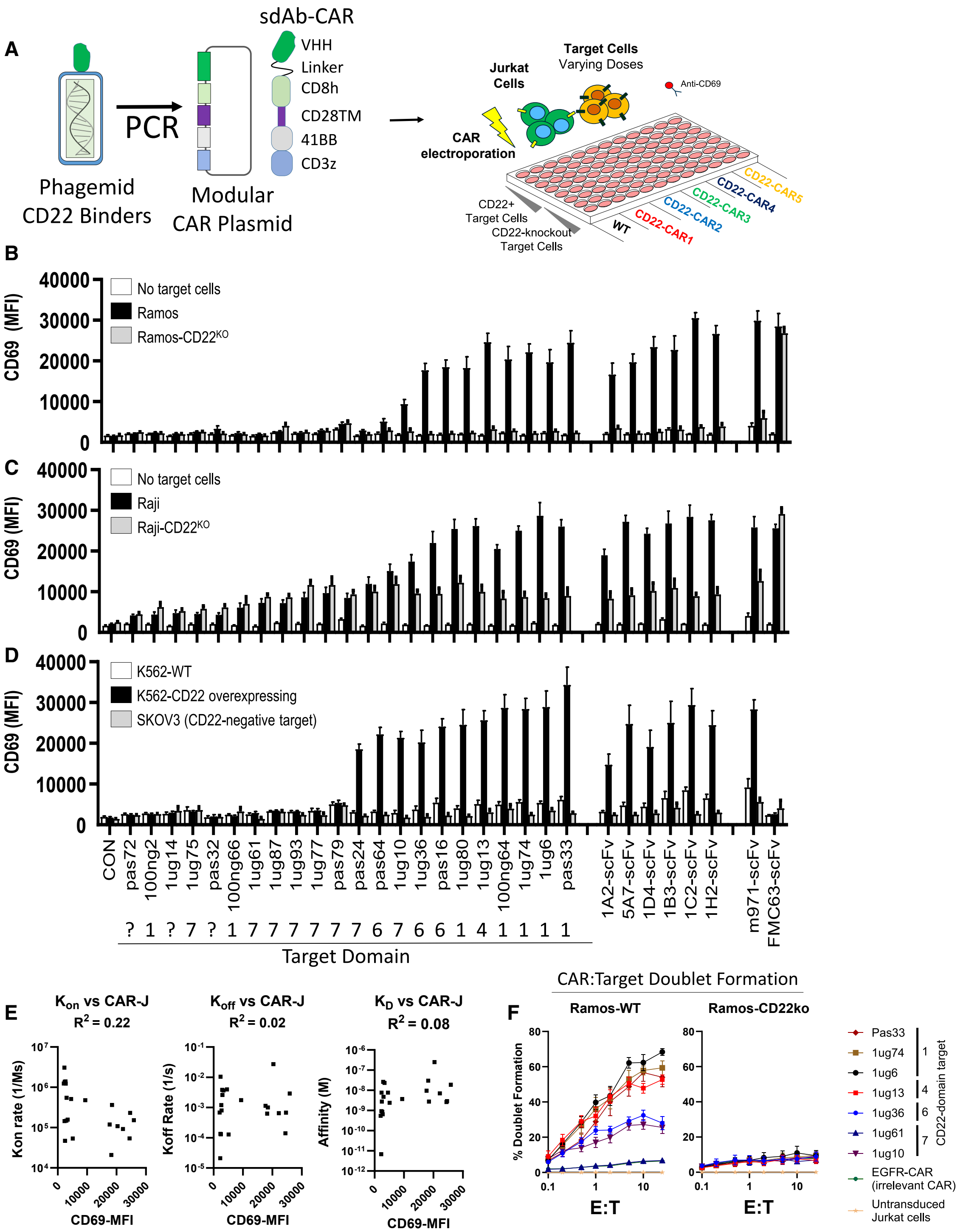

Figure 2. High-throughput CAR activity screening for sdAbs

(A) CD22-specific sdAbs were transferred directly from phagemid-VHH vectors into pSLCAR screening backbone via PCR and tested for CAR-Jurkat assay. Jurkat cells were electroporated with various CD22-sdCAR, CD22-scFv-CAR, or control scFv-CAR plasmids and immediately mixed at 1:1 E:T with various red fluorescent protein-marked target cells. (B) Ramos or Ramos-CD22KO cells. (C and D) Raji or Raji-CD22KO cells (C), or (D) K562-WT, K562-CD22-overexpressing, or irrelevant SKOV3 cells. After overnight coculture, cells were stained with anti-human CD69 antibody and assessed via flow cytometry. (E) The mean CD69 expression of all CD22sdCARs versus on-rate (k_{on}), off-rate (k_{off}), or affinity (K_D) derived from SPR measurements is shown. (F) CAR-Jurkat cell target-cell doublet formation was examined using coculture with CD22$^+$ Ramos cells or CD22KO cells for 30 min, followed by flow cytometry. Results show the mean of 3 experiments performed in duplicate ± SEM.

as measured by doublet formation (Figure 2F). We next examined the effect of truncated hinge domains in various CD22sdCAR constructs. Membrane-proximal targeting CARs (1ug36 and 1ug10) showed an immediate loss of CD22-response activity with hinge truncation, whereas other more membrane distal CARs (1ug13, 1ug6, 1ug74, and Pas33) maintained CD22 responsiveness regardless of the length of hinge domain used (Figure S4). Overall, these results identify a diverse set of CD22-responsive CD22sdCAR molecules with diverse epitope targeting, CAR cellular avidity, and CAR-activation properties.

Functional screening testing of CD22-sdAb CAR constructs in primary human T cells

Next, several CD22-sdAb CAR constructs with confirmed CD22 reactivity in the CAR-Jurkat assay were evaluated for activity in primary human T cells. Concentrated CD22sdCAR lentivirus was prepared, and healthy donor blood-derived human T cells were transduced to generate CD22sdCAR-T cells (see Table S2 for product characteristics). Green fluorescent CD22sdCAR-T cells were cocultured at low density in T cell-supportive conditions, with red fluorescent protein expressing $CD22^+$ target cells (Raji or Ramos cells) or CD22-KO target cells (Ramos-CD22KO). Across 3 initial screening experiments with CAR-T cells generated from 4 different healthy human blood donors, we observed variable target growth repression and expansion of CAR-T cells (Figures S5A–S5C; Table S2). Direct cytolytic killing assessment via chromium release assay showed weak cytolytic responses against $CD22^+$ target cells, with only 1ug36-BBz showing comparable specific cytotoxicity to m971 CD22-scFv CAR-T (Figures S5D and S5E).

We then performed a pilot *in vivo* test using immunodeficient Nod/SCID/interleukin-2 receptor (IL-2R)γ^{Null} (NSG) mice injected intravenously with human Ramos lymphoma cells stably expressing firefly luciferase (FLUC), followed by treatment on day 3 posttumor injection with a total T cell dose of 10^7 untransduced T cells (mock), CD19-targeted control CAR-T cells (FMC63), or 2 CD22sdCAR-T cells (1ug36 or 1ug13 constructs). Mice treated with 1ug36 CAR-T cells showed significantly longer survival and lower tumor engraftment than mice treated with similarly expanded human T cells without CAR lentivirus (mock) (Figures S6A–S6C). Importantly, we observed <50% survival at day 30 for all CAR-T treatments in this experiment, prompting us to explore alternative CAR designs to improve CAR-T performance.

CD8TM design improves CD22sdCAR-T expression and persistence *in vitro*

Based on the results of the preliminary assessments above, we selected 4 CD22sdCARs (1ug36, 1ug74, 1ug13, and Pas33) for testing in a CAR backbone that has previously been used for CAR delivery in an ongoing CD19 CAR-T clinical trial.[22,23] There were several differences between the initial CAR screening plasmid and the clinical trial such as plasmid used, the most significant being a change in the CAR structure from a CD28TM domain to a CD8TM domain (see Figure 3A). In the CAR-Jurkat assay, CD28TM and CD8TM CAR constructs showed similar responses, although baseline CD69 expression and nonspecific response to Ramos-CD22KO cells were consistently higher with CD8TM CAR-T constructs compared to CD28TM CAR-T constructs (Figure 3B).

In healthy donor PBMC-derived T cells, lentiviral transduction efficiency was lower with CD8TM CAR constructs. As with Jurkat T cells, the baseline expression of T cell activation markers CD25 and CD69 was also higher in CD8TM than CD28TM CAR-T cells (Figure 3C; Table S2). Antibody staining for surface sdCAR expression showed wide variation between constructs, but was consistently higher in CD8TM constructs (Figure 3D). CD28TM CAR cells showed higher overall expansion than CD8TM CAR constructs during the CAR-T production process (Figure 3E). Long-term assessment of CAR-T behavior in low-density culture revealed a progressive loss of green fluorescence in CD28TM-CAR cells, whereas higher CAR/GFP expression was maintained for several weeks in CD8TM CAR-T cells (Figure 3F). Importantly, more sustained GFP expression in CD8TM cultures did not correlate with antigen-independent expansion of T cells because overall cell confluence remained static (Figure 3G).

We then examined the behavior of the CAR-T cells in coculture with either $CD22^+$ or CD22KO target cells. Despite divergent baseline expression of CD69 and CD25, both CD28TM and CD8TM CAR constructs further upregulated activation markers, depending on target cell CD22 expression (Figures S7B–S7D). In long-term coculture, all of the CD22 CAR-T cells showed similar strong repression of target cell growth and minimal effect on CD22KO cells, regardless of CAR TM domain (Figure 3H). Looking at CAR-T cell expansion, CD8TM and CD28TM constructs appeared nearly identical in the presence of $CD22^+$ Raji or Ramos cells, but diverged strongly when cocultured with CD22KO cells (Figure 3I). Because others have shown that CD28TM CARs can cause dysregulation of endogenous CD28 signaling in T cells, we also examined CD28 expression in stable CAR-Jurkat with CD8TM or CD28TM CD22sdCARs. We find that CD8TM CARs showed higher CD28 expression than similar CARs with CD28TM domains (Figure S7E). Overall, these results indicated that a CD8TM CD22sdCAR design was favorable for further development due to higher CAR expression and persistence in the absence of antigen signaling.

CD22sdCAR lead selection

To identify a lead CD22sdCAR construct, we next performed a functional comparison of the 4 CD8TM-CD22sdCAR candidate molecules implementing full-sample randomization and blinding and using a CAR-T production process mimicking the cell manufacturing approach for an ongoing CD19 CAR-T clinical trial.[22] CAR-T products were generated and characterized (see Table S2) before being cryopreserved, anonymized, and transferred to a separate site for *in vitro* and *in vivo* testing. Consistent with the results above, *in vitro* functional testing showed highly similar CD22-specific repression of target cell growth across all constructs (Figures S7A–S7F). We note divergence in CAR-T responses to irrelevant $CD22^-$ target cells, with the 1ug13 CD22sdCAR and m971-scFv CAR showing the highest nonspecific responses (Figures S8F and S8G).

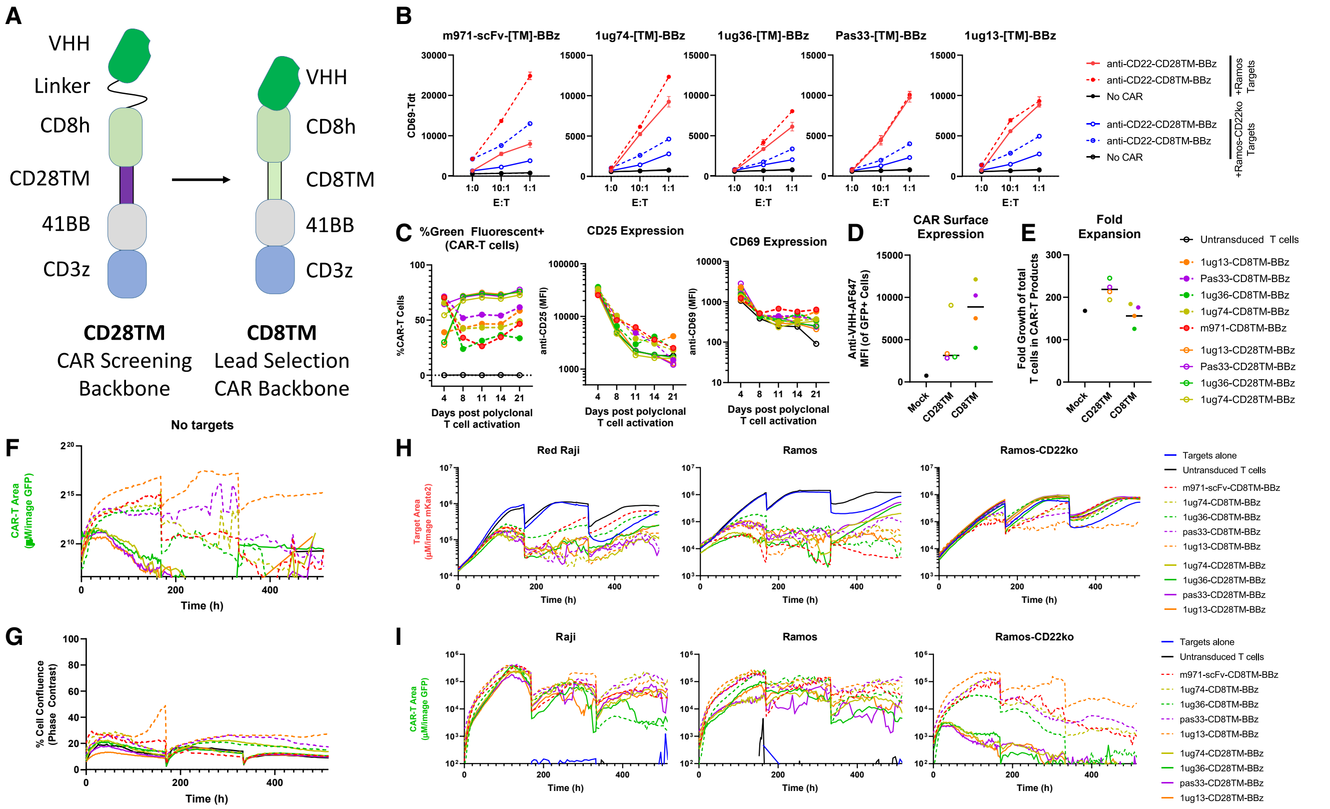

Figure 3. CD8TM CAR design increases CAR persistence

(A) Diagram of CAR design used for initial screening experiments (CD28TM) and for lead selection studies (CD8TM) in which the hinge and transmembrane domains were changed. (B) CD22-specific CD28TM and CD8TM CARs with various antigen-binding domains were examined via CAR-Jurkat assay with CD22$^+$ Ramos cells (red lines) or CD22KO cells (blue lines). (C) Primary CAR-T cells were generated from healthy donor PBMCs and transduced with CD22-CAR lentivirus before being examined for the expression of green fluorescence, CD25, and CD69 expression at various time points after transduction. (D) CAR-T cells were stained with an in-house broadly reactive anti-VHH reagent to assess CAR surface expression. (E–G) Total fold expansion of CD28TM or CD8TM CD22 CAR-T cell products at harvest is shown (E). CAR-T cells were then placed in low-density culture with IL-7/IL-15 supplementation and examined for (F) CAR-T cell signal via green fluorescence without target cells or (G) the total T cell number via confluence measurement. (H and I) Similarly, CAR-T cells were cocultured with CD22$^+$ Raji cells, Ramos cells, or Ramos-CD22KO cells and examined for (H) red fluorescent target cell growth and (I) green fluorescent CAR-T growth (bottom graphs). Each graph presents the mean of 2 duplicate wells from a single experiment.

Direct cytolytic killing assays showed results similar to those observed in our earlier screens, with m971 and 1ug36 showing the highest level of CD22$^+$ target killing and no cytolytic response against CD22$^-$ targets (Figure 4A). Prestimulation with CD22$^+$ target cells further increased target killing for 1ug13, 1ug36, and m971 CD22 CAR-T constructs, with low activity for 1ug74 and Pas33 CD22sdCARs (Figure 4B). We further assessed 1ug36 or m971 CAR-T expansion when cocultured with noncancerous CD22$^-$ human donor skin fibroblasts (HDFs) or induced-pluripotent stem cell-derived endothelial cells, generated as previously reported.[24] Similarly, as observed with nonspecific cancer lines, m971 also showed higher CAR-T expansion in response to both HDF cells and endothelial cells than 1ug36 CAR-T cells (Figures S9A–S9C). Overall, these results indicate that 1ug36 CAR shows favorable on-target CD22 response activity with lower off-target response than the benchmark m971 CAR-T.

Our primary lead selection criterion was chosen to be the relative *in vivo* therapeutic effect for the candidate CD22sdCAR molecules; therefore, we proceeded with a blinded assessment of all 4 candidate CD22sdCAR molecules. Female NSG mice were injected intravenously with 5 × 10^4 human Ramos lymphoma cells expressing FLUC (Ramos-FLUC) and randomly assigned to cages. At day 3 post-tumor challenge, cage cohorts were randomly selected for treatment with blinded cell products containing 2.5 × 10^6 CAR-T cells or an equivalent total number of nontransduced T cells. After experimental unblinding, the data clearly showed that only mice treated with 1ug36 or m971 CAR-T cells had significantly increased survival relative to those treated with unmodified T cells (Figure 4C). Weekly *in vivo* imaging was performed to quantitate Ramos-FLUC engraftment until most control animals had reached humane endpoints at day 40 (Figure 4D). Mice treated with 1ug36, 1ug13, and m971 CAR-T cells showed significantly lower tumor engraftment over mice treated

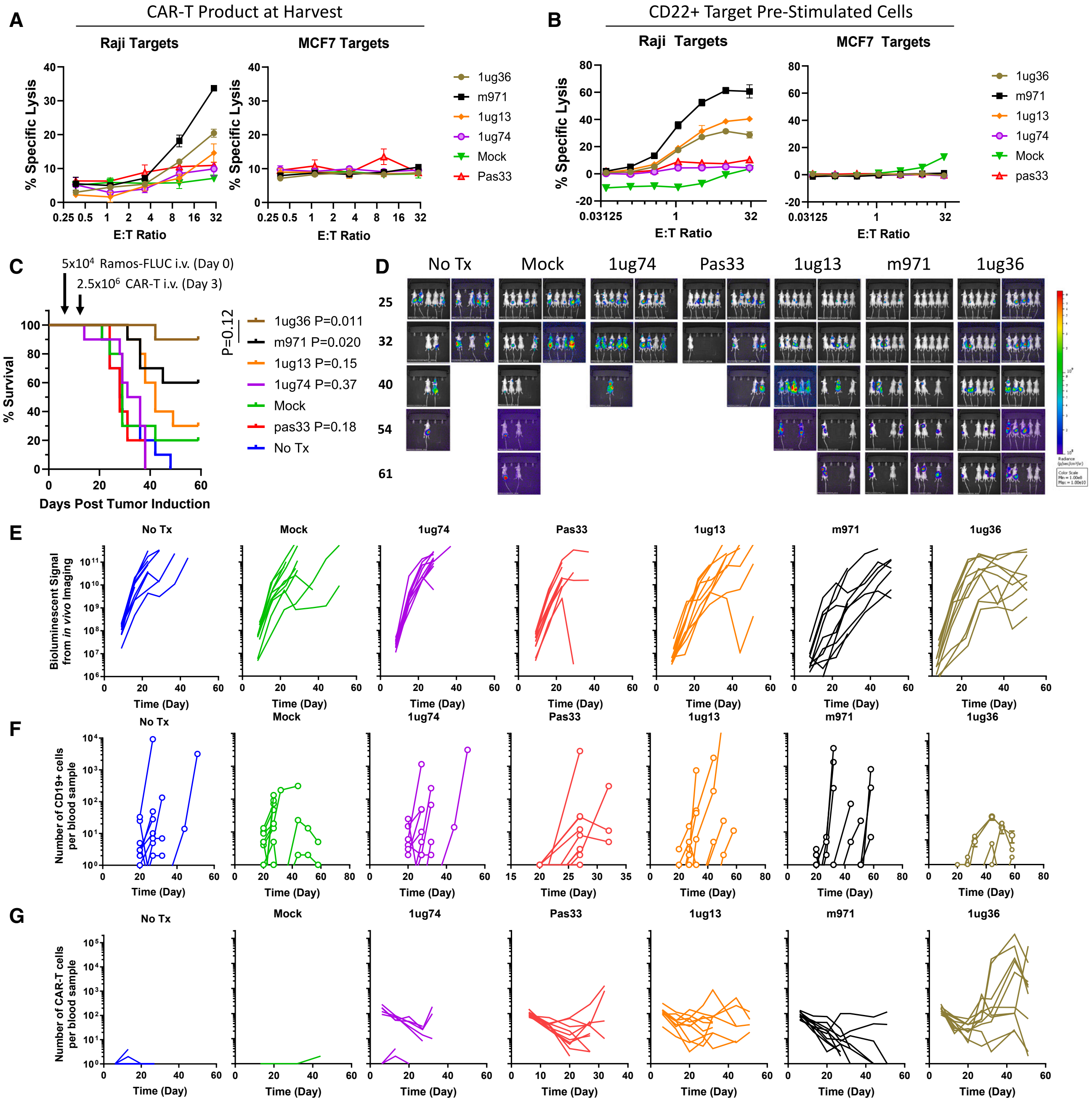

Figure 4. *In vivo* lead selection study for CD22sdCAR

CAR-T cells were generated from healthy donor apheresis product as described in the Materials and methods section and cryopreserved on day 14. (A) CAR-T functionality was assessed via chromium release cytolytic assay using CD22$^+$ Raji cells or CD22$^-$ MCF7 target cells. (B) CAR-T cells were stimulated with CD22$^+$ Ramos cells and allowed to return to rest after 18 days in culture, before repeated cytolytic cell killing assay. Results show the mean of 3 experiments performed in duplicate ± SEM. (C) Nod-SCID-IL-2Rγ-null (NSG) mice were then injected with 5 × 10^4 Ramos-FLUC cells and randomly assigned to cages. At day 3, cage groups were randomly assigned to treatment groups with 2.5 × 10^6 CD22 CAR-T cells or equivalent total dose of unmodified (mock) T cells (n = 10 mice per group). Mice were monitored for by distress and euthanized at predetermined humane endpoints. Graphs show the number of surviving mice at various time points. p values show the comparison of survival for treatment groups to untransduced mock T cells via the log-rank test. Mice were also assessed via IVIS imaging for bioluminescent signals from Ramos-FLUC tumors at various time points as shown in (D) images and (E) graphs displaying the biolumnescent signal detected per mouse over the timecourse. (F and G) Blood samples were also obtained at regular intervals for assessment of (F) circulating hCD45$^+$hCD19$^+$ Ramos cells or (G) hCD45$^+$hCD19$^-$NeonGreen$^+$ CAR-T cells via flow cytometry.

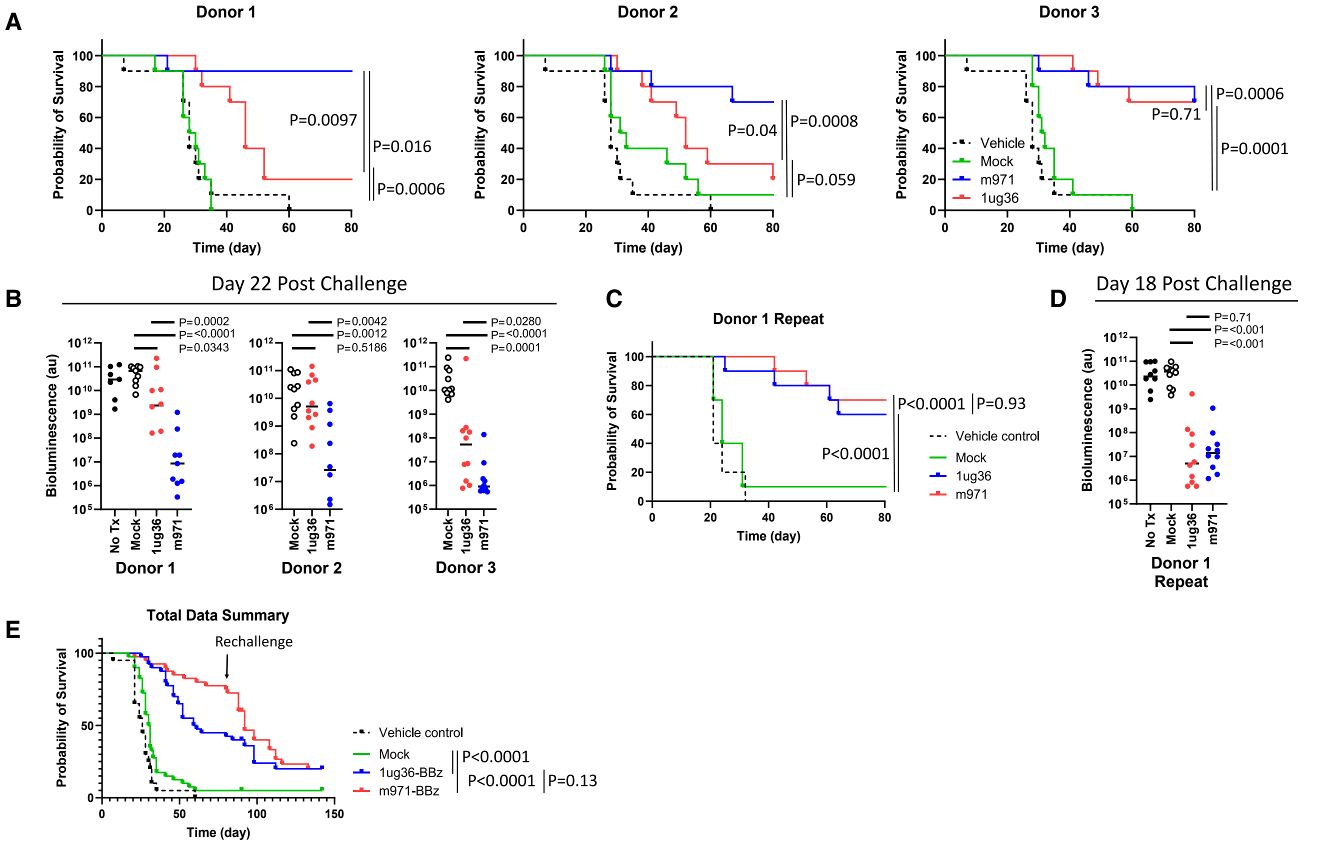

Figure 5. Confirmation of 1ug36-CD22sdCAR activity in multiple donor samples

NSG mice were injected with 5×10^4 Ramos-FLUC cells and randomly assigned to cages. At day 3, posttumor injection cage groups were randomly assigned to blinded treatment groups with nonfluorescently labeled 2.5×10^6 CD22 CAR-T cells or equivalent total dose of unmodified (mock) T cells generated from 3 different donor lymphocyte samples (n = 10 mice per group). Mice were monitored for by distress and euthanized at predetermined humane endpoints. (A) The number of surviving mice separated by donor sample is shown in survival graphs. (B) Mice were also assessed for tumor engraftment via IVIS imaging for bioluminescent signals from Ramos-FLUC tumors at day 22 after tumor challenge as shown. p values show comparisons using Student's t test with log-transformed bioluminescence values. (C and D) A similar experiment was repeated to confirm intradonor variability with this model wherein (C) mouse survival and (D) tumor load via bioluminescent imaging at day 18 after tumor challenge. (E) In the experiments above, after unblinding and analysis of survival, mice were rechallenged with an additional dose of 5×10^4 Ramos-FLUC cells at day 85 posttumor challenge. Pooled results for all of the experiments are shown (n = 40 mice per treatment group, 20 mice for untreated group). p values in survival graphs show intergroup comparisons via the log-rank test.

with unmodified T cells at early time points but not at later time points (Figures 4E and S10A). Weekly blood samples were also analyzed via flow cytometry to examine the number of circulating $CD19^+$ tumor cells (Figure 4F) and CD22-CAR-T cells (Figure 4G), with CAR-T cells expressing 1ug36-sdCAR showing the highest CAR-T expansion *in vivo*. Although we saw the highest number of circulating CAR-T cells for 1ug36 CAR-T treated mice (Figure S10B), we noted an overall lower proportion of CD8 expression on circulating CD22sdCAR-T cells relative to m971 CAR-T cells throughout the course of the experiment (Figure S10C). We further examined circulating CAR-T cells for expression of the programmed cell death protein 1 (PD-1) exhaustion marker (Figure S10D) and for the CAR-T cell differentiation phenotype (Figure S10E), but we did not see any consistent divergence between CD22sdCAR constructs or with benchmark CD22-scFv CAR. Based on these results, 1ug36-sdCAR was selected as the lead candidate for further *in vivo* validation.

Marginal dose 1ug36 CAR-T shows variable therapeutic effects

For a more complete comparison of m971-scFv and 1ug36-sdAb-CAR-T cells across multiple leukapheresis donors and using a preclinical CAR construct without the GFP marker, we generated CAR-T cells from 3 healthy donors and examined them in a blinded *in vivo* xenograft similar to that described above. Though both treatments improved survival, m971 yielded better survival and lower tumor load than 1ug36-sdCAR for 2 of 3 donors at this dose (Figures 5A and 5B). We also examined the proportion of $CD8^+$ T cells, the expression of PD-1, and the phenotypic differentiation markers among the circulating CAR-T cells in this study (Figure S11). Only the proportion of CD8 expression was consistently divergent between m971 and 1ug36

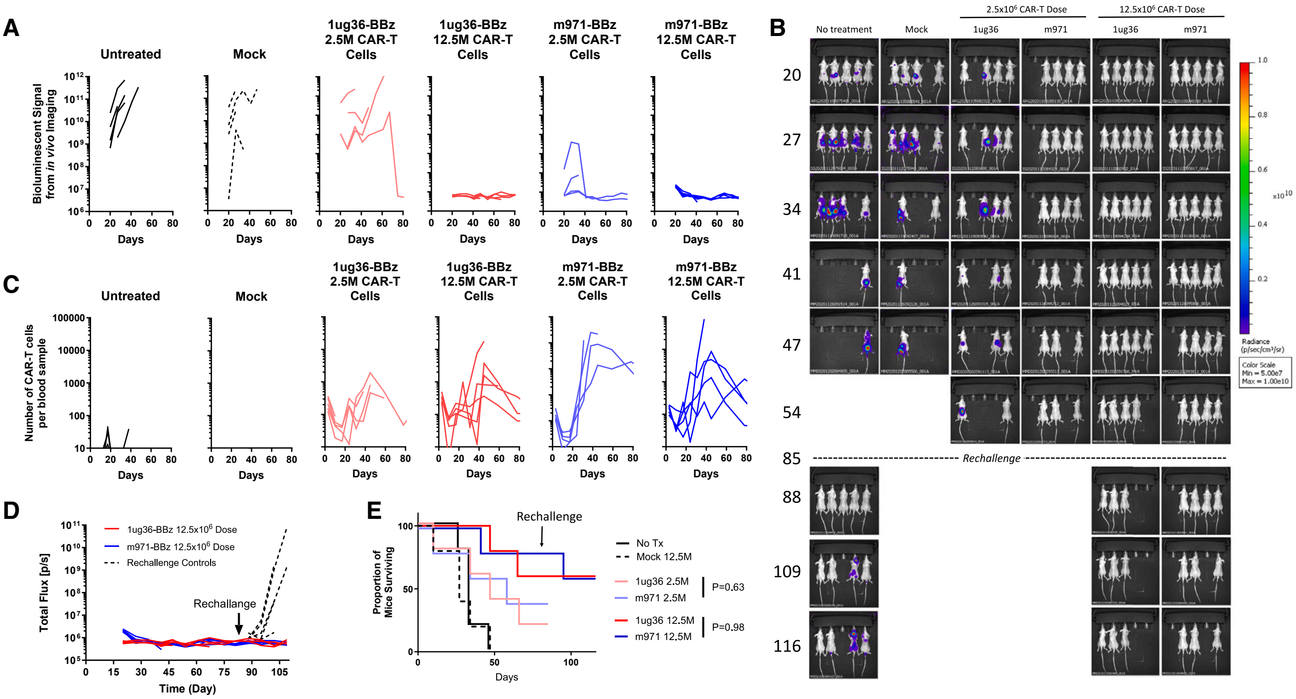

Figure 6. High-dose CD22sdCAR treatment leads to complete tumor responses

CAR-T cells were generated from healthy donor PBMCs as described in the Materials and methods section and prepared for injection on day 14 after transduction. NSG mice were injected with 5×10^4 Ramos-FLUC cells and randomly assigned to cages. At day 3, cage groups were treated with 2.5×10^6 (marginal dose) or 12.5×10^6 (high dose) CD22 CAR-T cells or equivalent to the highest total dose of unmodified (mock) T cells (n = 5 mice per group). Mice were assessed for tumor engraftment via IVIS imaging for bioluminescent signal from Ramos-FLUC tumors at various time points as shown in (A) bioluminescent signal data per mouse over the timecourse and in (B) mouse images. (C) Blood samples were also obtained at regular intervals for the assessment of $hCD45^+hCD19^-NeonGreen^+$ CAR-T cells via flow cytometry. (D) Surviving mice in the high-dose treatment group were rechallenged with an additional dose of 5×10^4 Ramos-FLUC cells intravenously, including new untreated control mice with no cell treatment. Graphs show the bioluminescent signals for 1ug36, m971, or untreated mice. (E) Proportion of surviving mice at various time points throughout the experiment is shown. p values show intertreatment group comparison at similar dose levels via the log-rank test.

CAR-T-treated mice, with 1ug36 CAR-T showing a lower proportion of CD8 T cells throughout the experiment. To further investigate the source of CAR-T variability, we repeated the *in vivo* experiment using the same CAR-T cell product generated from donor 1, in which we observed the greatest divergence between 1ug36 and m971 therapeutic effect. Contrary to the first study, the repeated *in vivo* experiment using an identical batch of donor 1 CAR-T cells showed an equal survival benefit and a reduction in tumor load with both 1ug36 and m971 CAR-T cells (Figures 5C and 5D). These results indicate that there can be a high degree of interexperimental variability even using the same CAR-T product. Surviving mice for all of the experiments were rechallenged intravenously with 5×10^4 Ramos-FLUC cells at day 85 after initial tumor challenge to test long-term protection. Pooling all of the experimental assessments (n = 40 per treatment group, n = 10 for untreated) we find no significant difference in survival benefit of 1ug36 and m971 treatment (Figure 5E).

Higher-dose 1ug36-sdCAR-T results in complete tumor eradication and protects against rechallenge

Although our blinded lead selection experiments showed a similar survival benefit for mice treated with 1ug36 or m971-scFv CAR-T cells, decreased tumor burden was transient. Thus, we next tested whether a 5-fold increase in CAR-T dose could lead to tumor eradication *in vivo*. Ramos-FLUC-implanted mice were treated with a marginal dose (2.5×10^6) or high dose (12.5×10^6) of either 1ug36-sdCAR-T, benchmark m971-scFv-CAR-T cells, or unmodified T cells at a similar high total cell dose (mock). Whereas marginal dose treatment of m971-scFv CAR-T again led to lower tumor signal than 1ug36 CD22sdCAR-T-treated mice, lymphoma growth was blocked completely in mice treated with a high dose of either CD22 CAR-T product (Figures 6A, 6B, and S12A). At day 85 posttumor challenge, surviving mice were rechallenged with 5×10^4 additional Ramos lymphoma cells; no tumor growth was detectable in rechallenged mice treated with m971 or 1ug36 CAR-T cells (Figures 6D and S12B). Overall, we conclude that 1ug36-sdAb and m971-scFv CAR show similar effects on mouse survival, and at higher doses, they are able to achieve similar complete responses.

Lead CD22sdAb shows lymphoid-specific tissue reactivity

Having selected 1ug36 as the lead candidate CD22sdAb molecule with *in vivo* therapeutic activity in the CAR format, we wished to perform an unbiased assessment of cell- and tissue-binding specificity

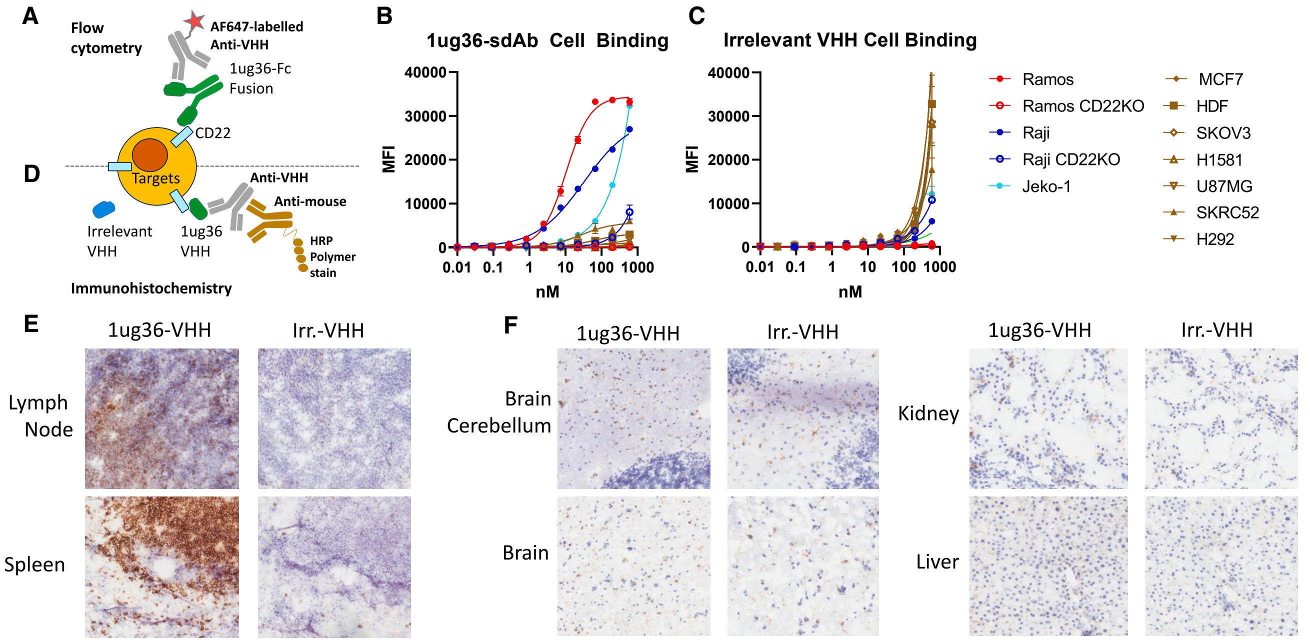

Figure 7. Absence of unexpected cell or tissue binding for 1ug36-sdAb

(A) Assay setup for flow cytometry staining approach. Graphs show the median fluorescence intensity across the primary antibody titration range for 3 CD22$^+$ cell lines (Raji, Ramos, Jeko-1), 2 CD22KO lines (Raji-CD22ko and Ramos-CD22ko), and 7 irrelevant target lines (MCF7, SKOV3, H1581, U87MG, SKRC52, H292, and HDFs) as measure via flow cytometry. Graphs show the results from a single experiment performed in duplicate using (B) 1ug36-Fc primary antibody or (C) a control irrelevant VHH-Fc antibody. (D) Assay setup for IHC staining approach of human frozen tissue array. (E) IHC images for lymphatic tissues in human tissue array. (F) Representative images for staining in nonlymphoid tissues. The full image set can be found in Table S3.

of this antibody. Purified 1ug36 or an irrelevant (bacterial protein specific) sdAb fused to a human Fc domain was produced and used to assess cell binding against a range of CD22$^+$ and CD22$^-$ target cell lines selected to reflect a diversity of tissue origin (MCF7, breast; HDF, primary dermal fibroblast; SKOV3, ovarian; H1581, lung; U87MG, brain; SKRC52, kidney; and H292, lung). Using an in-house fluorescently labeled mouse anti-sdAb for detection (Figure 7A), we detected strong binding to CD22-expressing Raji and Ramos cells, but no binding to various CD22KO or CD22$^-$ target cell lines (Figure 7B). As expected, nonspecific binding was observed with the irrelevant sdAb at high antibody concentrations (Figure 7C).

To examine specificity of 1ug36 binding over a wider range of human tissues, a frozen human tissue microarray (TMA) was directly stained with purified 1ug36 or irrelevant sdAb protein and probed with anti-sdAb secondary antibody, followed by anti-mouse tertiary staining. This revealed strong binding of 1ug36 CD22-sdAb in tissues with high concentrations of B cells such as lymph node and spleen (Figure 7D), whereas only weak nonspecific staining similar to that of the irrelevant sdAb was observed in key off-target tissues such as the brain, kidney, and liver (Figure 7E). An examination of slides via two blinded observers highlighted strong binding in lymphoid tissue, with weaker signal in nonlymphoid tissues (See Table S3). Given the known risk of immune effector cell-associated neurotoxicity syndrome with CD22-CAR therapies[25] and data showing that CD22 expression can be detected in aging human and mouse microglia,[26,27] brain assessments were of particular importance. Donor brain tissue samples in this array had a median age of 70 years (range 26–87 years), yet trained observers noted no specific staining with 1ug36 in brain samples (Figure 7F; Table S3). Although this approach may not detect very low levels or rare reactivity, these results indicate that 1ug36-sdAb is specific for human CD22-expressing cells and tissues, further validating it as a candidate for clinical translation.

DISCUSSION

We report here our efforts to identify a functional nanobody-based CD22-targeting CAR molecule. Although initial llama immunization yielded a large number of CD22-sdAb binders with varying binding properties, we found that only a subset of those molecules showed reactivity to the CD22 antigen when incorporated into CAR molecules. Furthermore, even within this subset of CD22-reactive CAR-sdAb molecules, we found that only relatively rare CAR-sdAbs targeting accessible membrane-proximal epitopes activated direct cytotoxicity and had therapeutic efficacy *in vivo* (see Figure 8).

It is well known that many complex factors, including antibody affinity, epitope specificity, and variable chain interaction properties, contribute to the antigen-dependent and antigen-independent signaling properties of scFv-based CARs,[28,29] but relatively few studies have comprehensively reported on the selection of sdAb

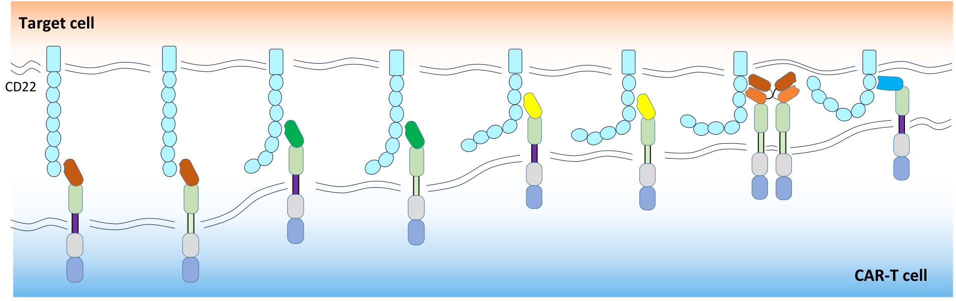

Antigen binding domain	1ug74 or Pas33	1ug74 or Pas33	1ug13	1ug13	1ug36	1ug36	m971	Several
Affinity (nM)	7.0/2.6	7.0/2.6	2.9	2.9	9.6	9.6	25*	0.5-7.7
CD22-domain targeting	1	1	4	4	6	6	7*	7
Cell binding	++	++	++	++	+	+	nd	low/-
CD22-dependent proliferation	++	++	++	++	++	++	++	low/-
Cytotoxicity	-	-	marginal	+	+	+	+	-
Transmembrane	CD28	CD8	CD28	CD8	CD28	CD8	CD8	CD28
CAR Persistence	-	+	-	+	-	+	+	nd
In vivo Activity	-	-	-	+	+	++	++	nd

*As per Ereño-Orbea et al. 2021

Figure 8. Model of interactions of various CD22sdCAR constructs with summary of observations

Data presented in this study indicate that membrane-distal CARs can induce activation and expansion of CAR cells, but do not have strong cytolytic activity or extend survival in an *in vivo* model. More membrane-proximal 1ug13 and 1ug36 with adequate cell-binding capacity show better cytolysis and can extend survival in a xenograft lymphoma model, similar to a benchmark CD22-targeting m971-scFv-CAR, which has been engineered for tonic signaling. We also tested several highly membrane-proximal CD22sdCARs that show low overall response to CD22-expressing targets.

moieties for CAR applications. Despite their relative simplicity, we find that identifying a highly functional CD22-specific CAR-sdAb required empirical screening efforts similar to those of scFv-CARs. Rapid functional assessments of CD22sdCARs in Jurkat cells revealed no correlation of CAR activity with sdAb-binding properties such as on-rate, off-rate, or monovalent affinity (ranging between 0.03 and 250 nM), a finding that is consistent with the current view that within the range of affinities tested, antibody affinity is a relatively poor predictor of binding domain activity in the CAR format.[30,31]

In contrast to antibody affinity, we found epitope location to be a key determinant in CD22-sdAb-CAR activity. Previous work has shown that membrane-proximal targeting is favorable for CD22 CAR responses,[32] although membrane-distal CD22-targeting elements can also activate antigen-specific T cell responses.[33] Consistent with these apparently contradictory observations, we find that CD22sdCARs targeting very-membrane-distal elements can indeed drive potent CAR-T activation and expansion but fail to activate cellular cytotoxicity against CD22-expressing targets. Recent mechanistic data using truncated CD22 or carcinoembryonic antigens provides convincing evidence that membrane-proximal targeting is specifically important for the exclusion of CD45 at the CAR cell-target cell synapse.[34] Because high-affinity T cell receptor ligands have been reported to induce T cell activation without CD45-exclusion,[35] our data may indicate that the cytolytic synapse is particularly sensitive to CD45 exclusion. Interestingly, we also find that CD22 reactivity is attenuated with hinge truncation for membrane-proximal but not membrane-distal epitope targeting CD22sdCARs, similar to what we have recently reported for epidermal growth factor receptor (EGFR)-targeted sdAb-CARs.[21] Whether hinge truncation may improve the cytolytic activity of membrane-distal CD22sdCARs will require additional experimental testing. Regardless of the specific mechanism, we find that for the CD22sdCAR constructs tested here, short-term cytolytic killing appears to be the best predictor of *in vivo* CD22-targeted CAR activity, and appears to be a more useful measure than assessment of activation marker upregulation or CAR-T expansion in the longer-term coculture assays we also report here.

Considering our translational intent, we focused on *in vivo* performance for the selection of a lead CD22sdCAR molecule, where we found that CARs targeting membrane-distal epitopes did not improve survival in a lymphoma xenograft model, whereas membrane-proximal targeting CD22sdCARs with confirmed cytotoxic activity showed significant therapeutic benefit *in vivo*. We note that this is not the first report of a camelid nanobody-cased CAR targeting CD22,[36] but given the data presented here showing that only relatively rare CD22-sdAbs can drive cytotoxicity and potent *in vivo* therapeutic activity, we believe that our lead CD22sdCAR is a stronger candidate for clinical translation than other molecules already reported.

During the development work reported here, we also tested CD22sdCAR candidate molecules in 2 different CAR plasmid backbones. We found that the CD8TM constructs maintained a higher level of activation and more sustained CAR expression. Recent data have shown that CD28TM CARs can dimerize with the endogenous CD28 receptor and drive the downregulation of surface CD28

expression on CAR-T cells.[37,38] Consistent with this, we also see lower surface expression of CD28 receptor in CD28TM than CD8TM containing CAR-expressing Jurkat cells. There is a wealth of data demonstrating that dysfunction or blockade in CD28 signaling can inhibit T cell responses,[39] potentially explaining the poor *in vitro* persistence of CD28TM CD22sdCAR constructs seen here. Interestingly, among widely deployed CD19-targeted CAR-T products, those containing CD28-transmembrane and signaling domains are generally associated with shorter persistence.[31]

This work adds to a large and growing body of preclinical and clinical data for CD22-targeted CAR therapies. A recent meta-analysis of CD22 CAR-T clinical trials for relapsed B cell acute lymphoblastic leukemia (B-ALL) found a mean complete response rate of 75%, and this was raised to 87% when combined with CD19-targeted CAR.[40] Because the majority of CD22-CAR patients treated across many clinical studies were previously treated with CD19-CAR, a direct comparison of CD19- and CD22-CAR response rates are not useful at this time. Nonetheless, clinical studies to date clearly demonstrate that potent antitumor responses with CD22 CAR-T are possible with safety/toxicity profiles similar to those of CD19 CAR-T.

Although our findings do not demonstrate the superiority of CD22sdCAR relative to the highly active short linker m971 CD22 scFv-CAR, we note a similar survival effect for both CARs over a large number of mice treated in blinded assessments. Furthermore, at a higher dose, 1ug36-CD22sdCAR was able to achieve potent long-term responses against human lymphoma in a xenograft model. Given the potential downstream advantages of sdAbs for CAR-T development (smaller size, structural simplicity, and easier multiantigen development), we feel that the clinical evaluation of such a therapeutically active single-target CD22sdAb-CAR is scientifically justified. We have initiated production of good manufacturing practices (GMP)-compliant lentiviral particles for a CD22sdCAR clinical trial, which will open in mid-2024, and to our knowledge would represent the first clinical trial of a CD22-targeted nanobody CAR. A clinical trial of this single-target CD22sdCAR therapeutic will serve as an important milestone toward future development of multiantigen targeted sdAb-CAR-T therapies for B cell malignancies.

MATERIALS AND METHODS

Immunogen preparation

Synthetic DNA encoding the ECD of the human CD22 receptor (aa 1–668; CD22-ECD) was gene synthesized via a commercial DNA supplier and cloned into the pTT5 mammalian expression vector via restriction cloning.[16] Protein production was performed using Chinese hamster ovary cells, and the expressed proteins in the culture supernatant were purified by immobilized metal affinity chromatography (IMAC) as previously reported.[16] The quantity of the protein was measured at 280 nm by a spectrophotometer, confirmed for size and purity via SDS-PAGE analysis, characterized by SEC-UPLC (data not shown), and preserved at −80C until further use.

Llama immunization and serum response analysis

A young male llama was immunized subcutaneously at 5 time points over 2 months with 100 μg of purified recombinant human CD22-ECD according to the protocol reported in Baral et al.[17] Briefly, CD22-ECD was mixed with complete Freund's adjuvant (prime injection) followed by 4 antigen boosts using incomplete Freund's adjuvant. The llama was bled at regular intervals following immunization, and sera from preimmune and terminal bleed (day 49) were used to confirm the serological response to CD22-ECD via ELISA. In brief, wells of an ELISA Nunc (Thermo Fisher) plate were coated with recombinant CD22-ECD at 5 μg/mL in PBS, blocked, and 2-fold serial dilution of llama serum (starting from 1/50 dilution) were added to wells; after washing, the binding was assessed using mouse anti-heavy-chain 5E4 monoclonal antibody.[41] All of the animal work was monitored and approved by the National Research Council's Human Health Therapeutics Research Centre (NRC-HHT) Institutional Animal Care Committee and was conducted in accordance with the Canadian Council of Animal Care (CCAC) guidelines.

sdAb amplification and phage library preparation

Blood from the seropositive llama was collected at day 49 and peripheral blood lymphocytes isolated using Lymphoprep tubes (Progen), as described previously.[17] Briefly, RNA extraction was performed using the Trizol lysis method (Thermo Fisher) before reverse transcription to generate a stable cDNA pool using Invitrogen SuperScript VILO cDNA Synthesis Kit (Thermo Fisher). The heavy-chain immunoglobulin repertoire was then amplified from the cDNA pool using a mixture of primers homologous to the llama antibody heavy-chain variable domain with broad sequence coverage within camelid heavy-chain variable genes in the framework 1 (FR1) region and 2 primers in the constant heavy-chain 2 region (CH2 and CH2b3), as described previously.[17] The heavy-chain bands (∼600–650 bp in length) were gel purified (Qiagene) and used as templates to amplify the sdAb fragments using MJ7 (FR1) and M78 (FR4). Amplified sdAb DNA was then used to construct an sdAb phagemid library in the pMED1 plasmid vector. sdAb antibody domains specific to the CD22 immunogen were then isolated by 2 phage panning approaches using sequential exposure to immobilized CD22-ECD or biotinylated CD22-ECD. Four rounds of selection were performed on an ELISA Nunn plate or on the captured biotinylated CD22 antigen on a streptavidin plate. After 4 rounds of panning using either strategy, sequencing data on 100 clones picked up randomly confirmed significant diversity among the anti-CD22-sdAbs. The binding of unique sdAb clonal phages to the recombinant CD22 was confirmed in phage ELISA.

Expression of anti-CD22 sdAbs

Unique sdAb sequences obtained after 4 panning rounds as described above were gene synthesized and cloned in an expression vector by a commercial DNA synthesis provider (Twist Bioscience), as previously reported.[18] The sdAb clones were expressed in *E. coli* and the periplasmic proteins were purified by IMAC followed by SEC using a Superdex 75 column (Cytiva) connected to an AKTA FPLC protein purification system (Cytiva), as described previously.[42] Monomeric

sdAb fractions in HBS-EP^+ buffer (10 mM HEPES, pH 7.4, containing 150 mM NaCl, 3 mM EDTA, and 0.05% [v/v] surfactant P20) were selected and used for SPR and epitope binning.

SPR and epitope binning

SPR was conducted on a Biacore T200 instrument (Cytiva) at 25°C in HBS-EP running buffer, as previously described.[42] In brief, ~1,900 resonance units of CD22-ECD and control EGFR protein (at concentrations of 250 μg/mL in 10 mM acetate buffer, pH 4.5) were immobilized on a Series S CM5 sensor chip using an amine coupling kit supplied by the manufacturer (Cytiva). An ethanolamine-blocked flow cell served as the reference. Monomeric sdAbs, SEC purified before SPR analysis, were injected over all of the surfaces at various concentration ranges (6.25–100 nM, 2.5–40 nM, 1.25–20 nM, or 0.625–10 nM, depending on the sdAb) in HBS-EP^+ buffer at a flow rate of 40 μL/min, with a 180-s contact time and 600–1,200 s of dissociation time, depending on the sdAb. For regeneration, the surfaces were washed with 10 mM glycine-HCl, pH 1.5) for 120 s at 30 μL/min. Binding to human CD22-ECD was studied using single-cycle kinetics analysis, with binding responses from an ethanolamine-blocked reference flow cell subtracted and fit to a 1:1 interaction model using BIAevaluation version 3.2. For epitope binning, the first sdAb (at ~50 × K_D concentration at a flow rate of 40 μL/min for 180 s) was injected over the CD22-ECD surface, followed immediately by a second injection containing a mixture of the first sdAb and a second sdAb (both at ~50 × K_D concentrations) at the same flow rate and contact time.

Epitope mapping of sdAbs to CD22 using yeast surface display (YSD)

To map the epitopes of the anti-CD22 sdAbs, fragments of hCD22-ECD corresponding to various hCD22 protein domains and sections were PCR amplified from the hCD22 DNA sequence and cloned into the YSD vector (catalog no. pPNL6, Pacific Northwest National Laboratory) by *in vivo* recombination in yeast. The hCD22-ECD and its 7 fragments containing aa1–668 (full ECD)—aa1–135, aa111–230, aa211–317, aa303–405, aa391–490, aa476–575, and aa561–668, corresponding to the aa numbering of mature hCD22—were expressed and covalently displayed on the surface of yeast as fusion proteins (Aga2-HA-(CD22)-MYC) under the inducing condition in galactose media. The binding of the sdAbs to yeast cells was performed using a whole yeast cell ELISA. Briefly, induced yeast cells were washed with 1× PBS, and 1 × 10^6 yeast cells were transferred per well to a 96-well filter plate (catalog no. MSDVN6550 MultiScreen DV Filter Plate, Millipore). After adhering cells, PBS was removed by vacuum aspiration and 200 μL of blocking solution (PBS with 2% BSA and 0.05% Tween 20; Sigma-Aldrich) was added, and plates were incubated for 45 min at room temperature with shaking. Blocking solution was removed and 100 μL of biotinylated sdAbs (100–250 nM), or mouse monoclonal anti-c-myc antibody (9E10) at 3 μg/mL (Sigma-Aldrich) was added and incubated for 1 h at room temperature with shaking. Cells were washed 4× with 200 μL/well PBS + 0.05% Tween 20. Detection of biotinylated sdAbs was performed using 100 μL horseradish peroxidase (HRP)-conjugated streptavidin (catalog no. 016-030-084, The Jackson Laboratory) at 1/10,000 dilution in 1% BSA + 1× PBS + 0.025% Tween 200, and MYC-tag was detected with 100 μL/well of HRP-F(ab)2 fragment goat anti-mouse immunoglobulin G (IgG) (H + L) (catalog no. 115-036-062, The Jackson Laboratory) at 1/5,000 in the same binding solution. The mixture was incubated for 1 h at room temperature with shaking and then washed 4× with PBS +0.05% Tween 20, followed by adding 100 μL/well of 3,3′,5,5′-tetramethylbenzidine substrate solution (catalog no. 34021, Thermo Fisher) and incubated for 10 min at room temperature, before stopping with 0.2 M sulfuric acid. Absorbance at 450 nm (optical density 450) was measured using a Tecan Infinite M1000 Pro plate reader.

Cell lines and cell culture

All of the cell lines were monitored regularly for mycoplasma contamination using an in-house PCR assay.[43] In preparation for the cell assays, healthy cultures of Jurkat E6-1 cells (catalog no. TIB-152, American Type Culture Collection [ATCC]#) were maintained in RPMI complete (RPMI 1640 supplemented with 10% fetal bovine serum [FBS], 2 mM L-glutamine, 1 mM sodium pyruvate, and 100 μg/mL penicillin/streptomycin), with cell densities between 0.25 and 1 M cells/mL for several weeks; we have found this to be critical for consistent results in Jurkat activation assays. All of the target cell lines described in this paper were modified using the Nuclight-Red Lentiviral reagent (catalog no. 4625, Sartorius) to generate stable red fluorescent cells, which can be easily differentiated from effector cells in flow cytometry or live microscopy analyses. Specific target lines used were as follows: Raji (catalog no. CCL-86, ATCC), Ramos (catalog no. CRL-1596, ATCC), SKOV3 (catalog no. HTB-77, ATCC), MCF7 (catalog no. HTB-22, ATCC), H1581 (catalog no. CRL-5878, ATCC). Target cell lines were cultured in varying media conditions, as recommended by the ATCC cell repository.

CAR cloning and CAR-Jurkat assay

High-throughput assessments of CAR function were performed by the CAR-Jurkat assay according to our previous report.[19] The CD22-specific sdAb sequences used here can be found in McComb et al.[44] Briefly, sdAb sequences were cloned from phagemid DNA plasmid into a modularized CAR backbone with the design [CD28 signal peptide]-[sdAb]-[(G4S)3linker]-[CD8hinge domain]-[CD28 transmembrane domain]-[41BB-signaling domain]-[CD3z signaling domain]-[P2A]-[NeonGreen Fluorescent Protein]. This modular CAR plasmid was redesigned from the plasmid as reported in Bloemberg et al.[19] (see catalog no. 135992, Addgene) to allow the exchange of only the antigen-binding domain while retaining the CAR structure, including the hinge domain. CD22-sdAb sequences were subcloned using primers, including appropriate type IIs restriction sites and a single-pot restriction ligation reaction, as reported previously. Plasmids were then miniprepped to isolate purified CD22sdCAR plasmids, and the sequence was confirmed via Sanger sequencing. The short linker m971-benchmark CAR plasmid was generated using a commercially synthesized scFv fragment including appropriate type IIs restriction site and overhangs, which was then cloned into the

modular CAR vector as described above, with the following amino acid sequence as per Orentas et al.[20]:

QVQLQQSGPGLVKPSQTLSLTCAISGDSVSSNSAAWNWIRQ
SPSRGLEWLGRTYYRSKWYNDYAVSVKSRITINPDTSKNQF
SLQLNSVTPEDTAVYYCAREVTGDLEDAFDIWGQGTMVT
VSSGGGGSDIQMTQSPSSLSASVGDRVTITCRASQTIWSYLN
WYQQRPGKAPNLLIYAASSLQSGVPSRFSGRGSGTDFTLTISSL
QAEDFATYYCQQSYSIPQTFGQGTKLEIK

Following this, 5×10^5 WT Jurkat cells were suspended in 100 μL Immunocult-XF media (catalog no. 10981, STEMCELL Technologies) and incubated for at least 1 min at room temperature with 2 μg of various simple lentiviral CAR plasmid (pSLCAR)-CAR plasmids as specified in the text or with no plasmid control. Cells and plasmid DNA in solution were transferred into 0.2-cm electroporation cuvettes (Bio-Rad Gene Pulser; Bio-Rad) and immediately electroporated using a Nepagene Super Electroporator with the following settings: 2 poring pulses of 175 V, pulse length 5, interval 50, decay rate 10, and polarity positive, and 5 transfer pulses of 20 V, pulse length 50, interval 50, decay rate 40, polarity +/−. Following electroporation, cells were transferred immediately in prewarmed recovery media (RPMI containing 20% FBS, 1 mM sodium pyruvate, and 2 mM L-glutamine) for 4 h before being cocultured with various target cells, as specified in the text. Electroporated Jurkat cells were mixed with varying numbers of target cells in round-bottom 96-well plates in effector-to-target (E:T) ratios ranging from 1:10 to 100:1 or with no target cells (or an E:T of 1:0) and cultured overnight before being stained with allophycocyanin-conjugated anti-human-CD69 antibody (catalog no. 555533, BD Biosciences). Jurkat-CAR target cell avidity was measured via 30-min coculture followed by flow cytometry, as previously reported.[21] Flow cytometry acquisition was performed using a BD-Fortessa (BD Biosciences), and data were analyzed using FlowJo software and visualized using GraphPad Prism.

Human T cell transduction: Screening studies

Different CAR-T production conditions were used in the work presented here (see Table S2 for details on different conditions tested). For initial screening studies, PBMCs derived from healthy donors were used to generate products, as previously described.[19] In brief, negative pan T cell selection was performed via negative magnetic selection according to the manufacturer's instructions (Miltenyi Biotech), followed by T cell activation and then transduction at day 1 after activation. High-concentration lentiviral particles encoding various sdCAR constructs were first generated as previously described, and aliquots were cryopreserved for later use.[21] Heparinized whole blood was then collected from healthy donors by venipuncture and transported at room temperature from the Ottawa Hospital Research Institute, as per research ethics board (REB)-approved protocol. Blood was diluted 1:1 with Hank's balanced salt solution (HBSS) and PBMCs were isolated by Ficoll-Paque density gradient centrifugation. In brief, PBMCs were then resuspended and counted before activation with Miltenyi MACS GMP T cell TransAct CD3/CD28 beads (catalog no. 130-111-160, Miltenyi Biotec) and seeded 1×10^6 T cells/mL in expansion medium (either serum-free TexMACS T cell expansion medium [catalog no. 130-097-196, Miltenyi Biotec] or serum-free StemCell Immunocult-XF [catalog no. 10981, STEMCELL Technologies]). Media was supplemented with 20 U/mL IL-2 (Novartis, Proleukin), or 3,100 U/mL human IL-7 (catalog no. 130-095-363, Miltenyi Biotec) and 160 U/mL IL-15 (catalog no. 130-095-765, Miltenyi Biotec). After 24 h of T cell stimulation with beads, T cells were transduced with sdCAR lentiviral vectors (MOI = 1 or 10) by spinfection at 850 × *g* for 2 h at 32°C in conical bottom tubes. After centrifugation, cells were incubated at 37°C for another 2 h before washing to remove lentivirus. After incubation, cells were plated in a 24-well plate (100,000 cells/mL/well in a total of 1.5 mL) in TexMACS medium supplemented with IL-7 and IL-15. Media was added at 48 and 72 h posttransduction to return cells to 150,000 cells/mL. CAR-T cells were propagated until harvest on days 9–14 to assess the efficiency of transduction and to characterize T cell subpopulations by flow cytometry.

CAR-T production for lead selection and multi-donor studies

For lead selection studies, CARs with designs as described in the text were synthesized via commercial gene synthesis provider and cloned into a modular lentiviral backbone. Following this, a manufacturing process similar to clinical CAR-T production was used. CD22sdCAR lentiviral particles were generated using HEK293T (clone 17; ATCC) as previously described.[45] Briefly, envelope, packaging, and transfer plasmids were cotransfected into the packaging cell line HEK293T (clone 17; ATCC) using TransIT-LT1 (catalog no. MIR2305, Mirus). Medium containing the lentivirus was collected after transfection, filtered, and then ultracentrifuged at 25,000 rpm for 90 min at 4°C (Optima XE-90 [Beckman-Coulter], with SW 32 Ti swinging bucket rotor) to pellet the virus. Viral pellets were resuspended in 1× Dulbecco's PBS and aliquoted for long-term storage at −80°C. The lentiviral particle concentration was determined by transducing HeLa cells with serial dilutions. Cells were analyzed for CAR expression either by GFP coexpression or by staining using an in-house broadly reactive mouse anti-sdAb antibody reagent by flow cytometry 3 days posttransduction.

Lentiviral particles were then used for human T cell transduction as follows: cryopreserved cells that had been previously positively selected via mixed CD4 and CD8 selection from a healthy donor apheresis using the CliniMACS Prodigy (Miltenyi Biotec) were thawed, activated with TransAct (Miltenyi Biotec), and cultured at 1×10^6 cells/mL in 24-well plates (1 mL/well × 4–6 wells/condition). Cells were cultured at 37°C, 5% CO_2, in 0.2 μM-filtered TexMACS media (Miltenyi Biotec) supplemented with IL-7 and IL-15 (12.5 ng/mL each, Miltenyi Biotec), gentamicin (5 μg/mL), and 3% human AB serum (catalog no. 100-612, Gemini Cell Plus, human AB). One day later (day 1), cells were transduced with lentiviral vectors or left untransduced (mock). On day 5, all of the wells from each condition were pooled, resuspended in fresh media, and transferred to G-Rex 10M flasks (Wilson Wolf) in a final volume of 25 mL. Cells were fed with partial media changes and gradual addition of media up to a maximum of 100 mL/flask. Cultures were split into 2 flasks if they

reached 7 × 10^8 cells. On days 12–14, cells were harvested, resuspended in Plasmalyte supplemented with 0.5% human serum albumin, combined with an equal volume of CryoStor CS10 (BioLife Solutions), and frozen in cyrovials and CS50/CS250 bags using a controlled rate freezer (Planar). CAR-T manufacturing was performed using nonfluorescently labeled CAR constructs for multidonor study, as described here, with lentiviral titration and T cell transduction rate being confirmed via flow cytometry using fluorescein isothiocyanate-labeled CD22 protein (catalog no. CD2-HF254, Acro Biosystems).

Continuous live-cell imaging coculture assay

Long-term response of the CAR-T cells was assayed using a Sartorius IncuCyte S3 (Essen Bioscience), as recently reported.[21] In brief, tumor target cells were resuspended in StemCell ImmunoCult-XF with 3,100 U/mL human IL-7 and 160 U/mL human IL-15 and plated in a flat-bottom 96-well plate (2,000 cells/well). CAR-T cells or control T cells were added into each well in a final volume of 200 μL/well in StemCell ImmunoCult-XF with 20 U/mL IL-2 at varying E:T ratios as specified in the text and cocultured for 7 days at 37°C. Images were taken at regular intervals in phase-contrast and examining red (excitation [ex]. 565–605 nm; emission [em]. 625–705 nm) or green fluorescence (ex. 440–480 nm; em. 504–544 nm). Automated cell counting of red (target) or green (CAR-T) cells was performed using IncuCyte analysis software, and data were graphed using GraphPad Prism. An 80% cell dilution passage and media feed with the addition of 2,000 fresh targets was performed weekly for cocultures that were maintained beyond 7 days.

Direct ^{51}Cr-release cytotoxicity assay

^{51}Cr-loaded Raji (CD22$^+$) and MCF7 (CD22$^-$) tumor targets (10,000/well) were mixed in triplicate with graded amounts of CAR-T cells in a final 100-μL volume per 96-U-bottom well. Following a 4.5-h incubation (37°C), PBS (50 μL) was added to all of the wells and supernatant (50 μL) from centrifuged plates (100 × g) was removed and mixed with scintillation cocktail (150 μL, Optiphase, PerkinElmer) in 96-well Flex plates (PerkinElmer). Activity was enumerated with a Microbeta Trilux scintillation counter (PerkinElmer). The percentage of specific lysis was calculated according to:

$$\%\ Specific\ lysis = \left(\frac{experimental\ release - spontaneous\ release}{maximum\ release - spontaneous\ release}\right) * 100,$$

where spontaneous release is the activity from tumor cells alone and maximum release is obtained from wells treated with 1% (w/v) cetrimide (Sigma-Aldrich). Data were processed using GraphPad Prism and displayed graphically as the mean ± SD with curve fitting.

Animal studies

NOD/SCID/IL-2R$\gamma^{-/-}$ (NSG; strain no. 005557, The Jackson Laboratory) mice were purchased from The Jackson Laboratory and maintained by the Animal Resource Group at the NRC of Canada. Ramos cells stably expressing FLUC were generated using transduction with a commercial lentiviral vector (GlowCell-15p, BiOSETTIA FLUC-T2A-RFP-IRES-Puro Lentivirus). Eight-week-old NSG mice were injected with 5 × 10^5 Ramos-FLUC in 100 μL of HBSS subcutaneously. For the initial study, Ramos-implanted mice were treated at 3 days posttumor injection retro-orbitally with a total of 10 million total T cells. For lead selection study and multidonor functional assessment studies, mice were randomized for cage assignment after tumor injection and cages were then randomly assigned for CAR-T treatment injections, both using randomization tables generated using Microsoft Excel RAND() function. CAR-T samples or control T cells were injected at a normalized CAR-expressing cell dose based on flow cytometric assessment at the CAR-T manufacturing site; control (untransduced) T cells were injected at the same total T cell dose as the CAR-T product with the highest total cell number. For lead selection and multidonor studies, all of the assessments of animals were done with full blinding of those performing the experiments and analysis. Data were collected and analyzed concurrent with the experiment, and unblinding was predetermined to occur no earlier than when 50% of vehicle control mice (untreated) had reached humane endpoint. Tumor growth was assessed at various time points via the *in vivo* imaging system (IVIS) for FLUC bioluminescence. The primary endpoint was a decrease in mouse condition, typically manifested as hindlimb paralysis occurring between days 20 and 40 posttumor injection. Weekly bleeding was also performed to monitor circulating CAR-T and lymphoma cells via flow cytometry. (See Table S4 for the flow cytometry antibodies used.) Mice were euthanized when they met prespecified humane endpoints based on weight loss and animal condition. The study design was approved by the NRC-HHT Institutional Animal Care Committee and was conducted in accordance with CCAC guidelines. Tumor growth and survival (humane endpoint) curves were generated using and analyzed for statistical significance using the log-rank test (Mantel-Cox) using GraphPad Prism 9.

Human tissue immunohistochemistry of CD22-sdAb

The binding specificity of the 1ug36 sdCD22 binder was assessed via immunohistochemistry (IHC) using a commercial normal human TMA obtained from Biochain Institute. The tissue array (catalog no. T6234701) consisted of 30 tissue types (including lymphoid and nonlymphoid tissues), each from 3 individual donors (total of 90 sections). Tissue sections (5–10 μm thick) were subjected to IHC using a Leica Bond III Automatic IHC Stainer (Leica Microsystems). Frozen human TMA slides were first air dried for 1 h at room temperature, followed by fixation for 10 min in a cold methanol:acetone (1:1) solution. Slides were then dried again for 1 h at room temperature and reconstituted in 1× Tris-buffered saline with 0.1% Tween 20 detergent before loading the slides in the Leica Bond III automated stainer.

The BOND Polymer Refine Detection Kit (catalog no. DS9800, Leica Biosystems) contained all of the reagents needed to perform automated IHC staining. Endogenous peroxidase activity was first inactivated using a peroxide block step. Sections were then treated with Sniper (catalog no. BS966M, Biocare Medical) for 30 min, then a Human ChromoPure Human IgG Fc Fragment (catalog no. 009-000-008,

The Jackson Laboratory, 100 μg/mL) for another 30 min to minimize endogenous background staining. This was then followed by the addition of the primary antibody, the 1ug36 anti-sdCD22 sdAb or an irrelevant sdAb B131 targeting *Clostridium difficile* toxin B, both used at a concentration of 0.5 μg/mL for 30 min. This was followed by the addition of the secondary antibody, an in-house anti-sdAb mouse monoclonal (mouse IgG F233-3H12 anti-VHH) that is known to bind to the variable heavy-chain domain of llama sdAb. Both the primary and secondary antibodies were produced at NRC. Binding of the secondary antibody was then detected using a tertiary probe (rabbit anti-mouse, included in the Polymer Refine Detection kit). Finally, a polymer detection reagent (HRP polymer anti-rabbit IgG; also included in the Polymer Refine Detection kit) that consists of HRP enzymes and anti-rabbit IgG antibodies attached to a dextran polymer was used as the detection agent, and positive staining was visualized following the addition of 3,3′-diaminobenzidine chromogen and hematoxylin counterstain.

The stained TMAs were examined independently by two trained pathologists (The Ottawa Hospital). The examiners were blinded to the identity of the treatment of the tissue samples. The individual tissues within the TMA were scored based on a staining intensity scale of 0, 1+, and 2+. The TMA scoring template was established before slide examination.

DATA AND CODE AVAILABILITY

The authors declare that all relevant data and critical experimental tools related to experiments presented here are included or will be made available upon request under reasonable agreement.

SUPPLEMENTAL INFORMATION

Supplemental information can be found online at https://doi.org/10.1016/j.omton.2024.200775.

ACKNOWLEDGMENTS

The research team acknowledges the important input and expertise from Dr. John Bell, Dr. Piriya Yoganathan, and Dr. Jennifer Quizi in the GMP process development for the CD22sdCAR lentiviral vector. We also acknowledge the hard work of many scientists and technical officers at the National Research Council Human Health Therapeutics Research Centre, The Ottawa Hospital Research Institute, and the BC Cancer Agency in support of the work presented here. This work was funded by the National Research Council Canada Disruptive Technology Solutions Cell and Gene Therapy challenge program, and BioCanRx.

AUTHOR CONTRIBUTIONS

S. McComb, M.A.-G., K.A.H., T.N., R.A.P., R.A.H., B.H.N., H.A., N.K., J.W., and R.D.W. contributed to conceptualizing this work and experimental design. Project oversight and management was provided by S. McComb, M.A.-G., M.S., and R.D.W. S. McComb, B.A.K., S.F., T.N., C.W., A.M., A.A., G.H., D.M.P., S. Ryan, S. Raphael, H.v.F., A.Z., Q.Z., S. Maclean, A.C., K.G., R.G., C.G., R.A.P., E.Y., and J.S.N. contributed to method development and protocol establishment for this work. The experiments were performed by S.F., M.R., T.N., C.W., A.A., G.H., D.M.P., S. Ryan, S. Raphael, H.v.F., A.Z., Q.Z., S. Maclean, A.C., K.G., C.G., R.A.P., E.Y., J.W., and J.S.N. Data analysis and interpretation were performed by S. McComb, M.A.-G., T.N., C.W., G.H., D.M.P., R.A.P., J.W., and R.D.W. S. McComb prepared the final figures and drafted the manuscript, which was reviewed and edited by M.A.-G., K.A.H., R.A.P., G.H., D.M.P., R.A.H., B.H.N., and R.D.W. All of the authors have read and approved the content of the manuscript.

DECLARATION OF INTERESTS

CD22-nanobody binding elements used in this work were disclosed in provisional patent filing US 2023/0265185 A1 and other provisional filings by the NRC. The authors declare no competing interests.

REFERENCES

1. Gill, S., and Brudno, J.N. (2021). CAR T-Cell Therapy in Hematologic Malignancies: Clinical Role, Toxicity, and Unanswered Questions (American Society of Clinical Oncology Educational Book), pp. e246–e265. https://doi.org/10.1200/EDBK_320085.

2. Xu, X., Sun, Q., Liang, X., Chen, Z., Zhang, X., Zhou, X., Li, M., Tu, H., Liu, Y., Tu, S., and Li, Y. (2019). Mechanisms of Relapse After CD19 CAR T-Cell Therapy for Acute Lymphoblastic Leukemia and Its Prevention and Treatment Strategies. Front. Immunol. *10*, 2664. https://doi.org/10.3389/fimmu.2019.02664.

3. Plaks, V., Rossi, J.M., Chou, J., Wang, L., Poddar, S., Han, G., Wang, Z., Kuang, S.-Q., Chu, F., Davis, R.E., et al. (2021). CD19 target evasion as a mechanism of relapse in large B-cell lymphoma treated with axicabtagene ciloleucel. Blood *138*, 1081–1085. https://doi.org/10.1182/blood.2021010930.

4. Atilla, P.A., and Atilla, E. (2022). Resistance against anti-CD19 and anti-BCMA CAR T cells: Recent advances and coping strategies. Transl. Oncol. *22*, 101459. https://doi.org/10.1016/j.tranon.2022.101459.

5. Pan, J., Niu, Q., Deng, B., Liu, S., Wu, T., Gao, Z., Liu, Z., Zhang, Y., Qu, X., Zhang, Y., et al. (2019). CD22 CAR T-cell therapy in refractory or relapsed B acute lymphoblastic leukemia. Leukemia *33*, 2854–2866. https://doi.org/10.1038/s41375-019-0488-7.

6. Fry, T.J., Shah, N.N., Orentas, R.J., Stetler-Stevenson, M., Yuan, C.M., Ramakrishna, S., Wolters, P., Martin, S., Delbrook, C., Yates, B., et al. (2018). CD22-targeted CAR T cells induce remission in B-ALL that is naive or resistant to CD19-targeted CAR immunotherapy. Nat. Med. *24*, 20–28. https://doi.org/10.1038/nm.4441.

7. Singh, N., Frey, N.V., Engels, B., Barrett, D.M., Shestova, O., Ravikumar, P., Cummins, K.D., Lee, Y.G., Pajarillo, R., Chun, I., et al. (2021). Antigen-independent activation enhances the efficacy of 41BB co-stimulated CD22 CAR T cells. Nat. Med. *27*, 842–850. https://doi.org/10.1038/s41591-021-01326-5.

8. Cheng, Q., Tan, J., Liu, R., Kang, L., Zhang, Y., Wang, E., Li, Y., Zhang, J., Xiao, H., Xu, N., et al. (2022). CD20-specific chimeric antigen receptor-expressing T cells as salvage therapy in rituximab-refractory/relapsed B-cell non-Hodgkin lymphoma. Cytotherapy *24*, 1026–1034. https://doi.org/10.1016/j.jcyt.2022.05.001.

9. Liang, A., Ye, S., Li, P., Huang, J., Zhu, S., Yao, X., Zhou, L., Xu, Y., Zhu, J., Zheng, C., et al. (2021). Safety and efficacy of a novel anti-CD20 chimeric antigen receptor (CAR)-T cell therapy in relapsed/refractory (r/r) B-cell non-Hodgkin lymphoma (B-NHL) patients after failing CD19 CAR-T therapy. J. Clin. Orthod. *39*, 2508. https://doi.org/10.1200/JCO.2021.39.15_suppl.2508.

10. Könning, D., Zielonka, S., Grzeschik, J., Empting, M., Valldorf, B., Krah, S., Schröter, C., Sellmann, C., Hock, B., and Kolmar, H. (2017). Camelid and shark single domain antibodies: structural features and therapeutic potential. Curr. Opin. Struct. Biol. *45*, 10–16. https://doi.org/10.1016/j.sbi.2016.10.019.

11. Arbabi Ghahroudi, M., Desmyter, A., Wyns, L., Hamers, R., and Muyldermans, S. (1997). Selection and identification of single domain antibody fragments from camel heavy-chain antibodies. FEBS Lett. *414*, 521–526. https://doi.org/10.1016/s0014-5793(97)01062-4.

12. Muyldermans, S. (2021). Applications of Nanobodies. Annu. Rev. Anim. Biosci. *9*, 401–421. https://doi.org/10.1146/annurev-animal-021419-083831.

13. Martin, T., Usmani, S.Z., Schecter, J.M., Roccia, T., Jackson, C.C., Deraedt, W., Yeh, T.M., Banerjee, A., Pacaud, L., Garrett, A., et al. (2023). Updated results from a matching-adjusted indirect comparison of efficacy outcomes for ciltacabtagene autoleucel in CARTITUDE-1 versus idecabtagene vicleucel in KarMMa for the treatment of patients with relapsed or refractory multiple myeloma. Curr. Med. Res. Opin. *39*, 81–89. https://doi.org/10.1080/03007995.2022.2139052.

14. Crocker, P.R., Paulson, J.C., and Varki, A. (2007). Siglecs and their roles in the immune system. Nat. Rev. Immunol. *7*, 255–266. https://doi.org/10.1038/nri2056.

15. Shah, N.N., and Sokol, L. (2021). Targeting CD22 for the Treatment of B-Cell Malignancies. ImmunoTargets Ther. *10*, 225–236. https://doi.org/10.2147/ITT.S288546.

16. Zhang, J., MacKenzie, R., and Durocher, Y. (2009). Production of chimeric heavy-chain antibodies. Methods Mol. Biol. *525*, 323–336. https://doi.org/10.1007/978-1-59745-554-1_17.

17. Baral, T.N., MacKenzie, R., and Arbabi Ghahroudi, M. (2013). Single-domain antibodies and their utility. Curr. Protoc. Im. *103*. Unit 2.17. https://doi.org/10.1002/0471142735.im0217s103.

18. Rossotti, M., Tabares, S., Alfaya, L., Leizagoyen, C., Moron, G., and González-Sapienza, G. (2015). Streamlined method for parallel identification of single domain antibodies to membrane receptors on whole cells. Biochim. Biophys. Acta *1850*, 1397–1404. https://doi.org/10.1016/j.bbagen.2015.03.009.

19. Bloemberg, D., Nguyen, T., MacLean, S., Zafer, A., Gadoury, C., Gurnani, K., Chattopadhyay, A., Ash, J., Lippens, J., Harcus, D., et al. (2020). A High-Throughput Method for Characterizing Novel Chimeric Antigen Receptors in Jurkat Cells. Mol. Ther. Methods Clin. Dev. *16*, 238–254. https://doi.org/10.1016/j.omtm.2020.01.012.

20. Orentas, R.J., Pastan, I.H., Dimitrov, D.S., and Mackall, C.L. (2020). M971 chimeric antigen receptors.

21. McComb, S., Nguyen, T., Shepherd, A., Henry, K.A., Bloemberg, D., Marcil, A., Maclean, S., Zafer, A., Gilbert, R., Gadoury, C., et al. (2022). Programmable Attenuation of Antigenic Sensitivity for a Nanobody-Based EGFR Chimeric Antigen Receptor Through Hinge Domain Truncation. Front. Immunol. *13*, 864868.

22. Kekre, N., Hay, K.A., Webb, J.R., Mallick, R., Balasundaram, M., Sigrist, M.K., Clement, A.-M., Nielsen, J.S., Quizi, J., Yung, E., et al. (2022). CLIC-01: Manufacture and distribution of non-cryopreserved CAR-T cells for patients with CD19 positive hematologic malignancies. Front. Immunol. *13*, 1074740.

23. Ottawa Hospital Research Institute (2022). Canadian-led Immunotherapies in Cancer: CLIC-1901 for the Treatment of Patients with Relapsed/Refractory CD19 Positive Hematologic Malignancies. clinicaltrials.gov.

24. Ribecco-Lutkiewicz, M., Sodja, C., Haukenfrers, J., Haqqani, A.S., Ly, D., Zachar, P., Baumann, E., Ball, M., Huang, J., Rukhlova, M., et al. (2018). A novel human induced pluripotent stem cell blood-brain barrier model: Applicability to study antibody-triggered receptor-mediated transcytosis. Sci. Rep. *8*, 1873. https://doi.org/10.1038/s41598-018-19522-8.

25. Morris, E.C., Neelapu, S.S., Giavridis, T., and Sadelain, M. (2022). Cytokine release syndrome and associated neurotoxicity in cancer immunotherapy. Nat. Rev. Immunol. *22*, 85–96. https://doi.org/10.1038/s41577-021-00547-6.

26. Olah, M., Menon, V., Habib, N., Taga, M.F., Ma, Y., Yung, C.J., Cimpean, M., Khairallah, A., Coronas-Samano, G., Sankowski, R., et al. (2020). Single cell RNA sequencing of human microglia uncovers a subset associated with Alzheimer's disease. Nat. Commun. *11*, 6129. https://doi.org/10.1038/s41467-020-19737-2.

27. Pluvinage, J.V., Haney, M.S., Smith, B.A.H., Sun, J., Iram, T., Bonanno, L., Li, L., Lee, D.P., Morgens, D.W., Yang, A.C., et al. (2019). CD22 blockade restores homeostatic microglial phagocytosis in aging brains. Nature *568*, 187–192. https://doi.org/10.1038/s41586-019-1088-4.

28. Jayaraman, J., Mellody, M.P., Hou, A.J., Desai, R.P., Fung, A.W., Pham, A.H.T., Chen, Y.Y., and Zhao, W. (2020). CAR-T design: Elements and their synergistic function. EBioMedicine *58*, 102931. https://doi.org/10.1016/j.ebiom.2020.102931.

29. Stoiber, S., Cadilha, B.L., Benmebarek, M.-R., Lesch, S., Endres, S., and Kobold, S. (2019). Limitations in the Design of Chimeric Antigen Receptors for Cancer Therapy. Cells *8*, 472. https://doi.org/10.3390/cells8050472.

30. Liu, X., Jiang, S., Fang, C., Yang, S., Olalere, D., Pequignot, E.C., Cogdill, A.P., Li, N., Ramones, M., Granda, B., et al. (2015). Affinity-tuned ErbB2 or EGFR chimeric antigen receptor T cells exhibit an increased therapeutic index against tumors in mice. Cancer Res. *75*, 3596–3607. https://doi.org/10.1158/0008-5472.CAN-15-0159.

31. Ghorashian, S., Kramer, A.M., Onuoha, S., Wright, G., Bartram, J., Richardson, R., Albon, S.J., Casanovas-Company, J., Castro, F., Popova, B., et al. (2019). Enhanced CAR T cell expansion and prolonged persistence in pediatric patients with ALL treated with a low-affinity CD19 CAR. Nat. Med. *25*, 1408–1414. https://doi.org/10.1038/s41591-019-0549-5.

32. Haso, W., Lee, D.W., Shah, N.N., Stetler-Stevenson, M., Yuan, C.M., Pastan, I.H., Dimitrov, D.S., Morgan, R.A., FitzGerald, D.J., Barrett, D.M., et al. (2013). Anti-CD22–chimeric antigen receptors targeting B-cell precursor acute lymphoblastic leukemia. Blood *121*, 1165–1174. https://doi.org/10.1182/blood-2012-06-438002.

33. Velasco-Hernandez, T., Zanetti, S.R., Roca-Ho, H., Gutierrez-Aguera, F., Petazzi, P., Sánchez-Martínez, D., Molina, O., Baroni, M.L., Fuster, J.L., Ballerini, P., et al. (2020). Efficient elimination of primary B-ALL cells in vitro and in vivo using a novel 4-1BB-based CAR targeting a membrane-distal CD22 epitope. J. Immunother. Cancer *8*, e000896. https://doi.org/10.1136/jitc-2020-000896.

34. Xiao, Q., Zhang, X., Tu, L., Cao, J., Hinrichs, C.S., and Su, X. (2022). Size-dependent activation of CAR-T cells. Sci. Immunol. *7*, eabl3995. https://doi.org/10.1126/sciimmunol.abl3995.

35. Al-Aghbar, M.A., Chu, Y.-S., Chen, B.-M., and Roffler, S.R. (2018). High-Affinity Ligands Can Trigger T Cell Receptor Signaling Without CD45 Segregation. Front. Immunol. *9*, 713.

36. Zhang, T., Wang, T., You, F., Li, Z., Chen, D., Zhang, K., Tian, S., Sheng, B., Wu, H., Jiang, L., et al. (2022). Nanobody-based anti-CD22-chimeric antigen receptor T cell immunotherapy exhibits improved remission against B-cell acute lymphoblastic leukemia. Transpl. Immunol. *71*, 101538. https://doi.org/10.1016/j.trim.2022.101538.

37. Leddon, S.A., Fettis, M.M., Abramo, K., Kelly, R., Oleksyn, D., and Miller, J. (2020). The CD28 Transmembrane Domain Contains an Essential Dimerization Motif. Front. Immunol. *11*, 1519. https://doi.org/10.3389/fimmu.2020.01519.

38. Muller, Y.D., Nguyen, D.P., Ferreira, L.M.R., Ho, P., Raffin, C., Valencia, R.V.B., Congrave-Wilson, Z., Roth, T.L., Eyquem, J., Van Gool, F., et al. (2021). The CD28-Transmembrane Domain Mediates Chimeric Antigen Receptor Heterodimerization With CD28. Front. Immunol. *12*, 639818.

39. Beyersdorf, N., Kerkau, T., and Hünig, T. (2015). CD28 co-stimulation in T-cell homeostasis: a recent perspective. ImmunoTargets Ther. *4*, 111–122. https://doi.org/10.2147/ITT.S61647.

40. Li, L., Wang, L., Liu, Q., Wu, Z., Zhang, Y., and Xia, R. (2022). Efficacy and safety of CD22-specific and CD19/CD22-bispecific CAR-T cell therapy in patients with hematologic malignancies: A systematic review and meta-analysis. Front. Oncol. *12*, 954345. https://doi.org/10.3389/fonc.2022.954345.

41. Henry, K.A., van Faassen, H., Harcus, D., Marcil, A., Hill, J.J., Muyldermans, S., and MacKenzie, C.R. (2019). Llama peripheral B-cell populations producing conventional and heavy chain-only IgG subtypes are phenotypically indistinguishable but immunogenetically distinct. Immunogenetics *71*, 307–320. https://doi.org/10.1007/s00251-018-01102-9.

42. Hussack, G., Arbabi-Ghahroudi, M., van Faassen, H., Songer, J.G., Ng, K.K.-S., MacKenzie, R., and Tanha, J. (2011). Neutralization of Clostridium difficile Toxin A with Single-domain Antibodies Targeting the Cell Receptor Binding Domain. J. Biol. Chem. *286*, 8961–8976. https://doi.org/10.1074/jbc.M110.198754.

43. Molla Kazemiha, V., Shokrgozar, M.A., Arabestani, M.R., Shojaei Moghadam, M., Azari, S., Maleki, S., Amanzadeh, A., Jeddi Tehrani, M., and Shokri, F. (2009). PCR-based detection and eradication of mycoplasmal infections from various mammalian cell lines: a local experience. Cytotechnology *61*, 117–124. https://doi.org/10.1007/s10616-010-9252-6.

44. McComb, S., Weeratna, R., Arbabi-Ghahroudi, M., Nguyen, T., and WU, C. (2023). Anti-cd22 single domain antibodies and therapeutic constructs.

45. Rive, C.M., Yung, E., Dreolini, L., Brown, S.D., May, C.G., Woodsworth, D.J., and Holt, R.A. (2022). Selective B cell depletion upon intravenous infusion of replication-incompetent anti-CD19 CAR lentivirus. Mol. Ther. Methods Clin. Dev. *26*, 4–14. https://doi.org/10.1016/j.omtm.2022.05.006.

OMTON, Volume *32*

Supplemental information

Discovery and preclinical development of a therapeutically active nanobody-based chimeric antigen receptor targeting human CD22

Scott McComb, Mehdi Arbabi-Ghahroudi, Kevin A. Hay, Brian A. Keller, Sharlene Faulkes, Michael Rutherford, Tina Nguyen, Alex Shepherd, Cunle Wu, Anne Marcil, Annie Aubry, Greg Hussack, Devanand M. Pinto, Shannon Ryan, Shalini Raphael, Henk van Faassen, Ahmed Zafer, Qin Zhu, Susanne Maclean, Anindita Chattopadhyay, Komal Gurnani, Rénald Gilbert, Christine Gadoury, Umar Iqbal, Dorothy Fatehi, Anna Jezierski, Jez Huang, Robert A. Pon, Mhairi Sigrist, Robert A. Holt, Brad H. Nelson, Harold Atkins, Natasha Kekre, Eric Yung, John Webb, Julie S. Nielsen, and Risini D. Weeratna

Supplemental Figures:

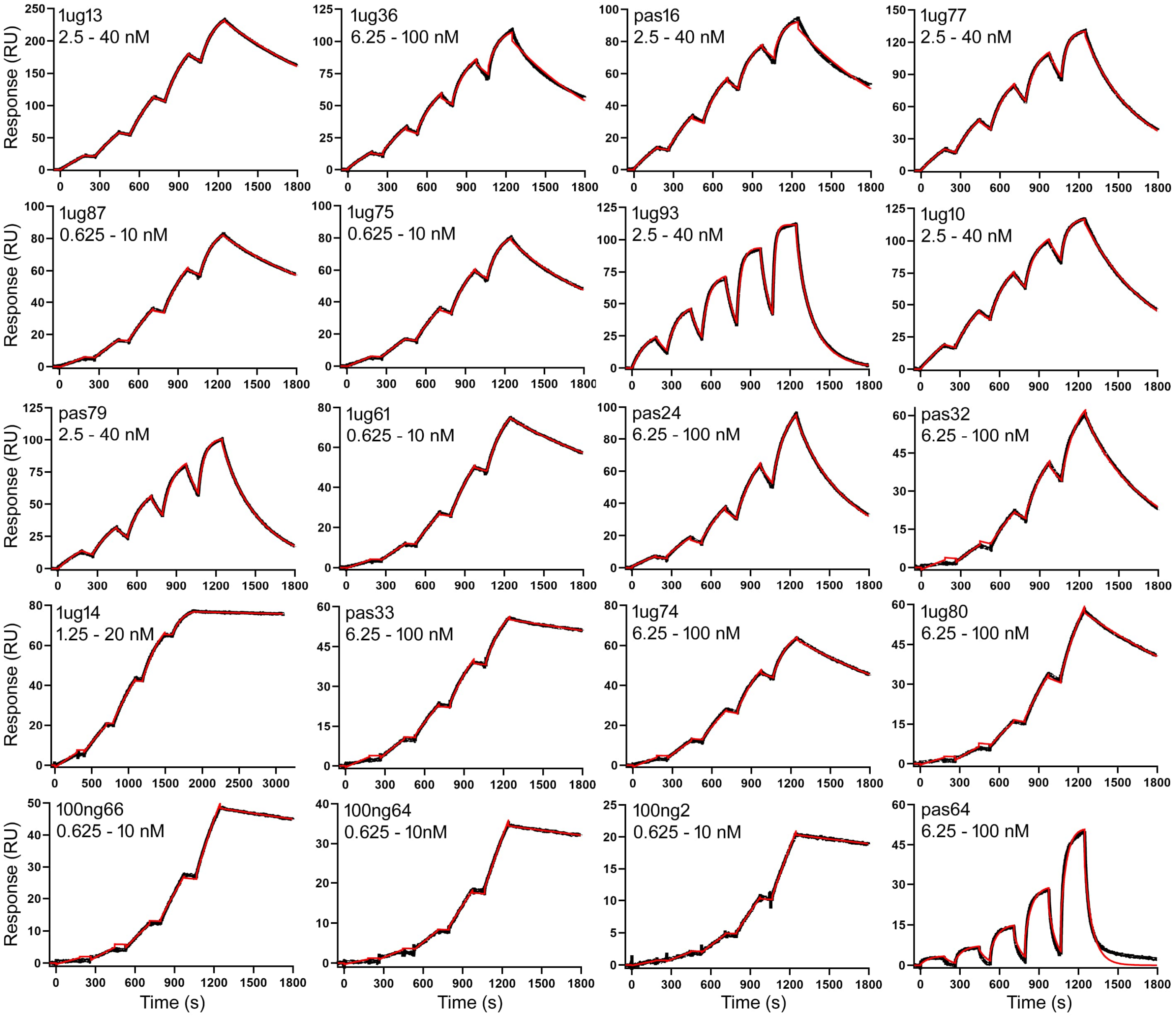

Figure S1: *Surface plasmon resonance sensorgrams for 20 CD22-specific single domain antibodies.* Human CD22 was amine coupled on a Series S CM5 sensor chip and SEC-purified sdAb monomers were injected at the concentration ranges indicated. Reference flow cell subtracted single-cycle kinetics sensorgrams were fit to a 1:1 interaction mode to determine kinetics and affinities (*see* Table S1). Black lines represent raw data, red lines represent 1:1 binding model fitting.

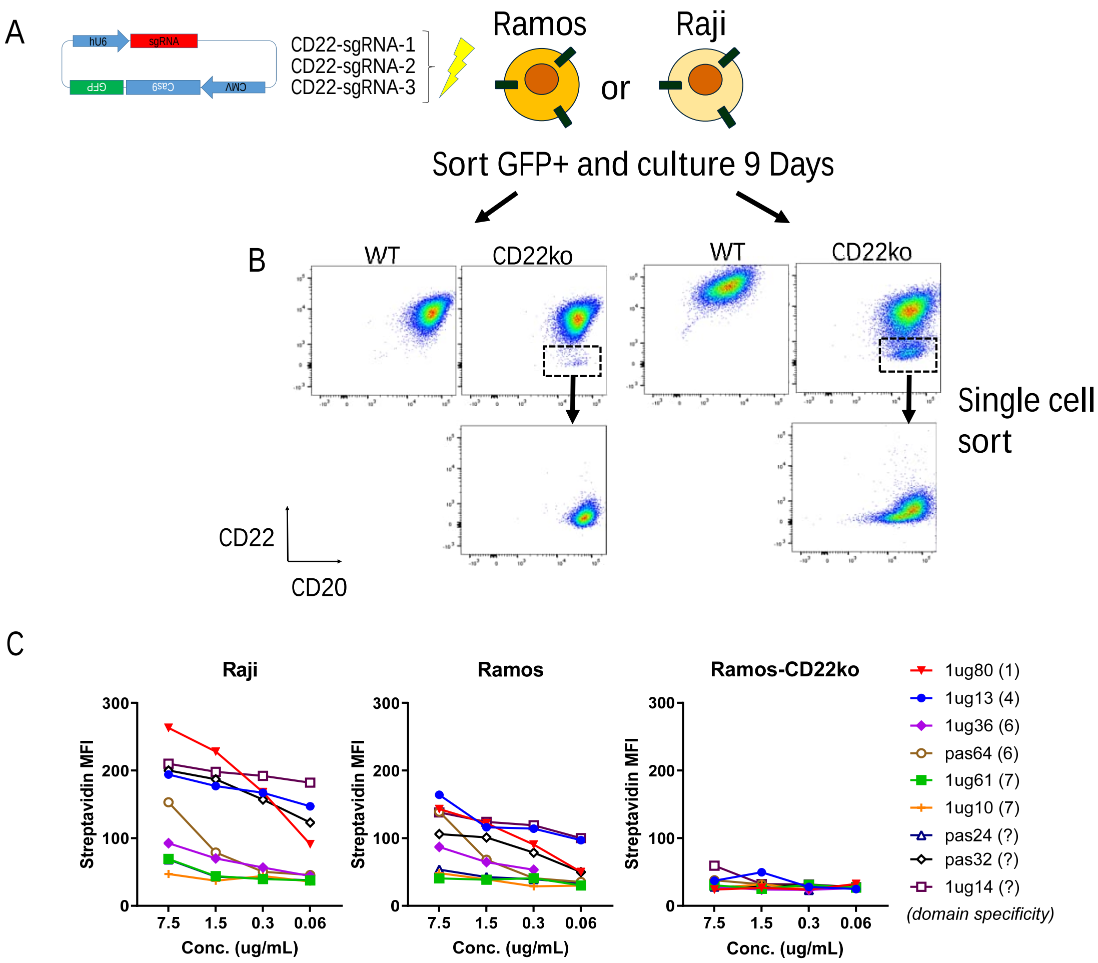

Figure S2: *Generating CD22-Knockout Raji and Ramos cell lines with CRISPR plasmids.* (a) Plasmids encoding 3 sgRNA sequences targeting the human CD22 gene were generated and electroporated into Ramos and Raji cells. After several days in culture, flow cytometry with anti-human CD22 and CD20 was performed, revealing a stable population of CD22 knockout cells. Cells were then single cell sorted to generate clonal knockout lines which were also (b) confirmed by flow cytometry to have no detectable CD22 expression, but maintained CD20 expression, which is characteristic of these lymphoma lines. (c) Cell binding with various biotinylated anti-CD22 sdAbs was performed to confirm staining against Raji and Ramos cells, but no staining against Ramos-CD22ko cells.

A

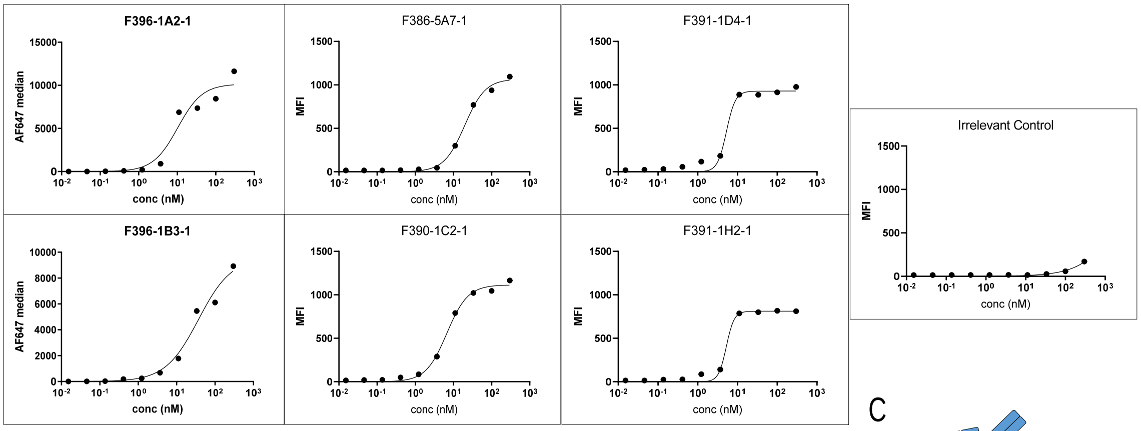

B

Yeast-surface Display Screening

Clone ID	Apparent Kd in Ramos Cell Binding	Bin	Membrane Distal → Membrane Proximal							Full Length CD22
			Domain 1	Domain 2	Domain 3	Domain 4	Domain 5	Domain 6	Domain 7	
F386-5A7-1	20.1	1	1.167							0.498
F391-1D4-1	5.3	2		0.503						0.840
F396-1B3-1	37.0	3			1.086					0.278
F390-1C2-1	6.8	3			1.415					0.706
F391-1H2-1	5.3	4				1.559				1.256
F396-1A2-1	10.3	4				1.922				1.271

C

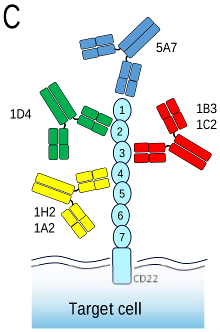

Figure S3: *Confirmation of cell binding and domain specificity for mouse anti-human CD22 monoclonal antibodies.* Mouse monoclonal antibodies targeting human CD22 were generated via immunization and hybridoma screening. (a) 6 anti-CD22 antibodies were purified and screened for cell binding against CD22-expressing Ramos cells, using a fluorescently labelled anti-mouse secondary antibody for detection. Cells were then examined via flow cytometry. (b) Purified antibodies were examined for binding to the CD22-domain yeast-surface display library similarly as described in the methods section. The binding to specific CD22-domains was assessed via yeast ELISA. The table shows the relative signal for the highest binding domain for each antibody tested. (c) Provides an overview of the antibody specificity for those mouse anti-CD22 antibodies tested here.

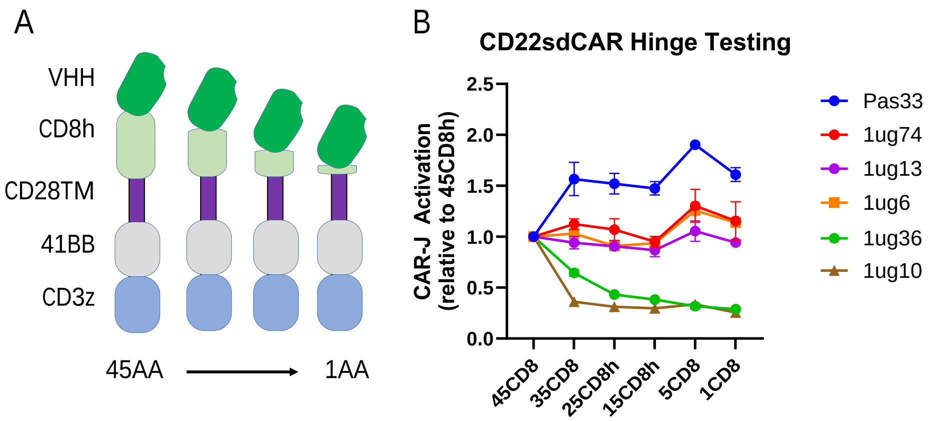

<u>Figure S4</u>: *CAR-T Activity Screening in Primary Human T cells.* T cells were isolated from healthy donor PBMC, polyclonally activated, and transduced with various CD22sdCAR lentivirus. At 9 to 10 days after polyclonal activation, green-fluorescent CAR-T cells or un-transduced T cells (*mock*) were placed in co-culture with red-fluorescent CD22+ target cells, Raji or Ramos, or CD22-deficient Ramos-CD22ko cells, at (a-b) a total T-cell to target ratio of 5:1 with IL-2 cytokine or (c) a 25:1 ratio with IL7/15 cytokines, with a constant 2000 target cells per well in a 96-well plate. Co-cultures were tracked via live microscopy at regular intervals to assess target cell growth (*top graphs*) and CAR-T growth (*bottom graphs*). Each graph presents the mean of two duplicate wells from a single experiment. (d) CD22sdCAR-T cells were also tested for short-term cytolytic response via chromium release assay as described in the methods section. Results show the mean of a single experiment performed in duplicate for two donors as shown, (e) graph shows the mean cytolysis at 40:1 E:T ratio from both donors +/- SEM, P-values shows the result of a student T test comparison of specific Raji killing with unmodified (mock) T cells.

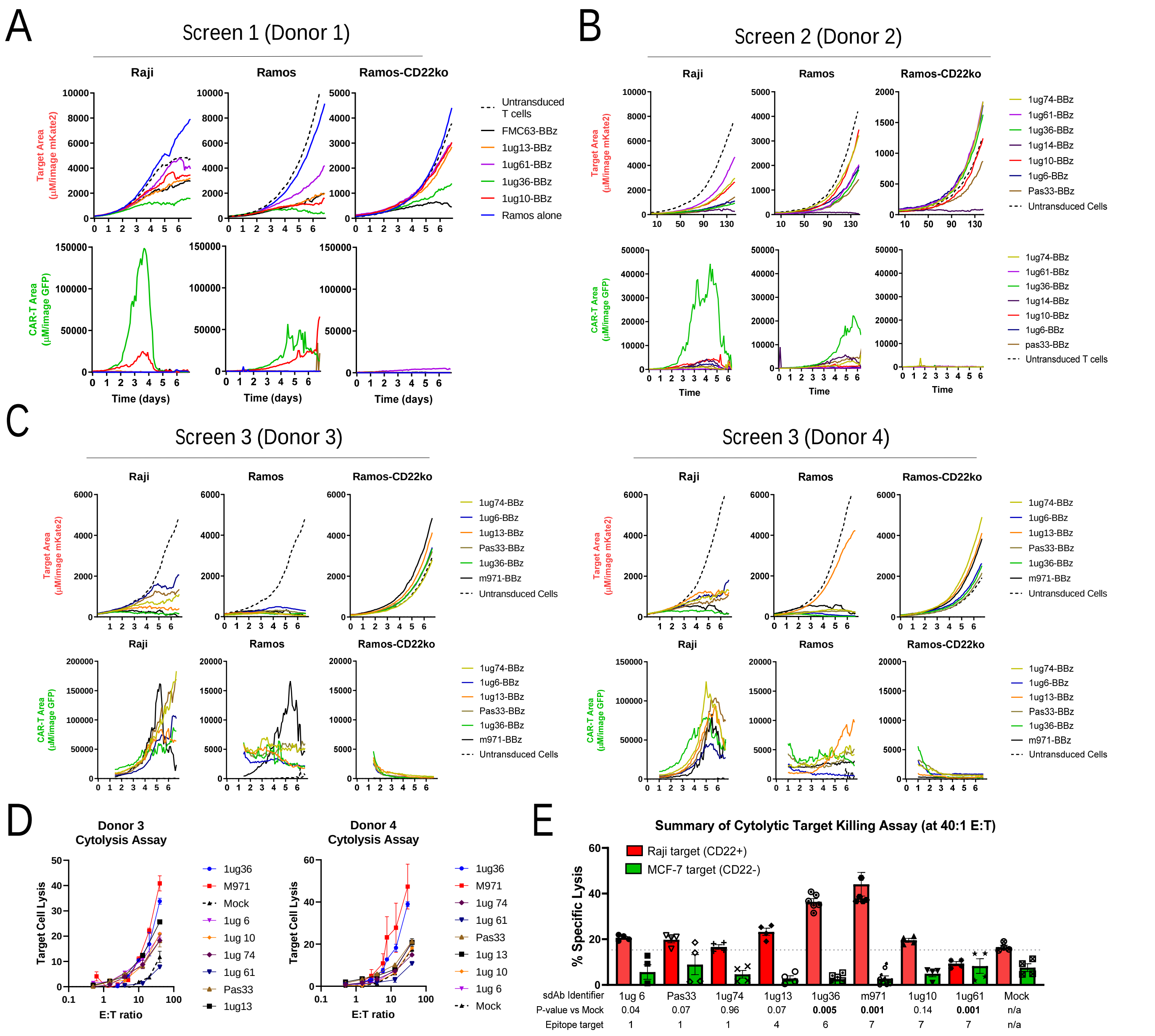

sdAb Identifier	1ug 6	Pas33	1ug74	1ug13	1ug36	m971	1ug10	1ug61	Mock
P-value vs Mock	0.04	0.07	0.96	0.07	**0.005**	**0.001**	0.14	**0.001**	n/a
Epitope target	1	1	1	4	6	7	7	7	n/a

<u>Figure S5</u>: *CAR-T Activity Screening in Primary Human T cells.* T cells were isolated from healthy donor PBMC, polyclonally activated, and transduced with various CD22sdCAR lentivirus. At 9 to 10 days after polyclonal activation, green-fluorescent CAR-T cells or un-transduced T cells (*mock*) were placed in co-culture with red-fluorescent CD22+ target cells, Raji or Ramos, or CD22-deficient Ramos-CD22ko cells, at (a-b) a total T-cell to target ratio of 5:1 with IL-2 cytokine or (c) a 25:1 ratio with IL7/15 cytokines, with a constant 2000 target cells per well in a 96-well plate. Co-cultures were tracked via live microscopy at regular intervals to assess target cell growth (*top graphs*) and CAR-T growth (*bottom graphs*). Each graph presents the mean of two duplicate wells from a single experiment. (d) CD22sdCAR-T cells were also tested for short-term cytolytic response via chromium release assay as described in the methods section. Results show the mean of a single experiment performed in duplicate for two donors as shown, (e) graph shows the mean cytolysis at 40:1 E:T ratio from both donors +/- SEM, P-values shows the result of a student T test comparison of specific Raji killing with unmodified (mock) T cells.

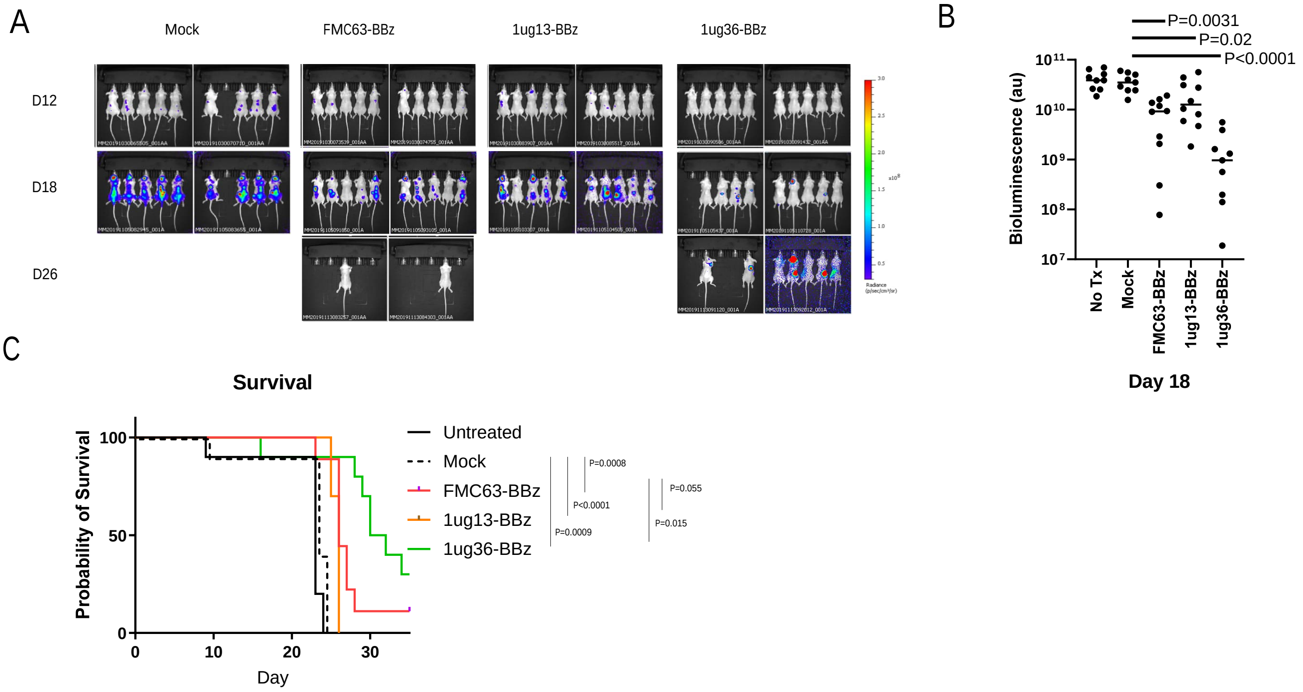

Figure S6: *Preliminary in vivo testing with CD22sdCAR-T cells.* Nod-SCID-IL2Rγ-null (NSG) mice were injected with $5x10^4$ Ramos-FLUC cells and randomly assigned to cages, at day 3 post-tumor mice were injected with 5×10^6 total T cells for various CAR-T products or unmodified (mock) T cells (n=10 mice per group). Mice were monitored for by distress and euthanized at pre-determined humane endpoints. (a) Mice were assessed via IVIS imaging for bioluminescent signal from Ramos-FLUC tumours at various timepoints as shown. (b) Shows the relative bioluminescent signal at the last timepoint where control mice were surviving (day 18 post tumor injection). P values shows a student T test comparison of mock and CAR-T treatment groups. (c) The number of surviving mice at each timepoint after tumour challenge is shown in survival graphs. P values show the comparison of survival for treatment groups via Log-Rank test.

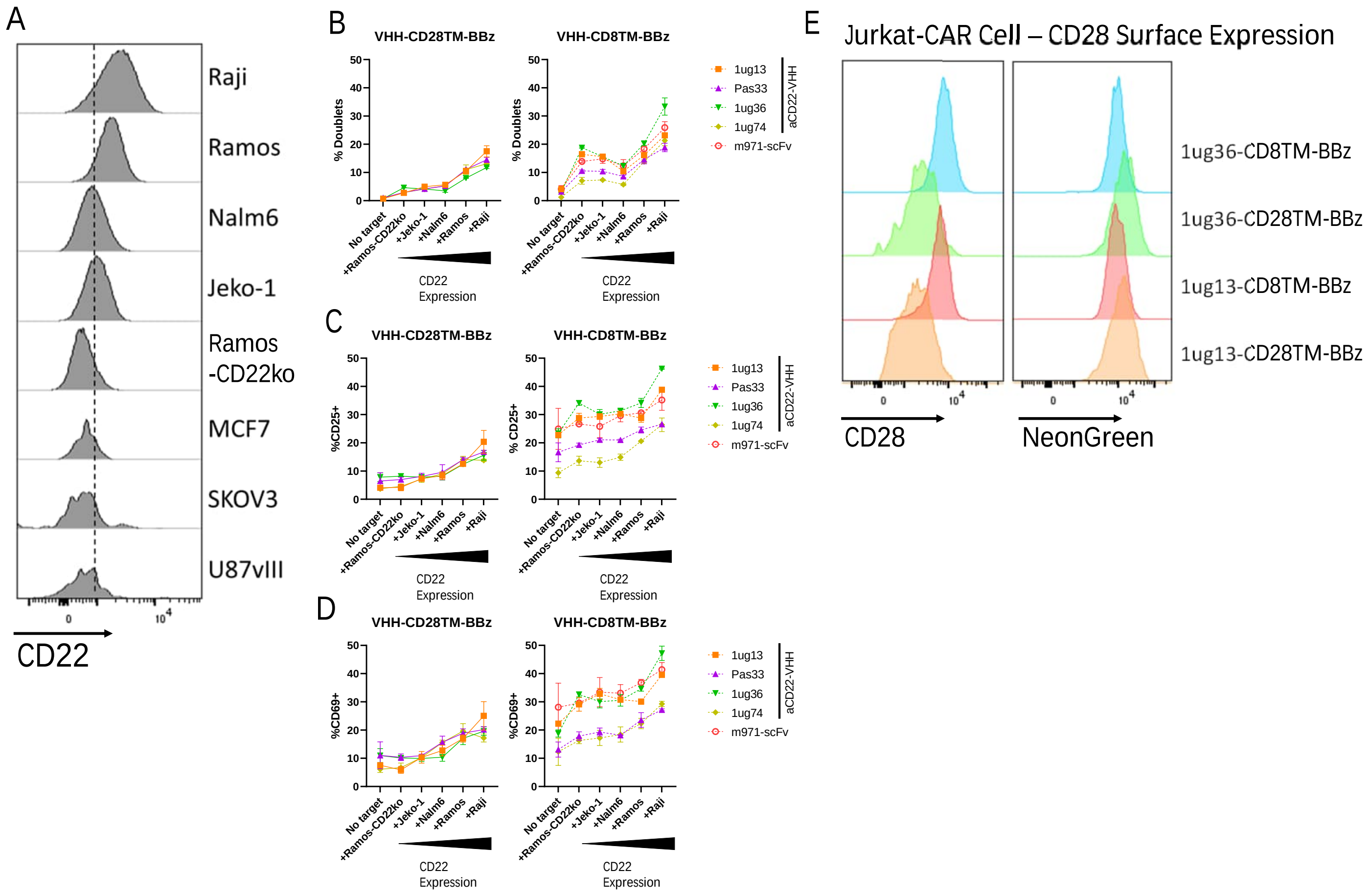

Figure S7: *Response of CD28TM or CD8TM CAR-T cells to various CD22 expressing cells and CD28 surface expression.* (a) Various target cell lines were examined for their relative CD22 expression using flow cytometry. (b-d) CAR-T cells were placed in overnight co-culture at high density with various target cells at an E:T ratio of 1:1. Graphs show the mean result from duplicate wells in a single experiment. (e) Jurkat cells stably expressing CD28TM or CD8TM versions of CD22sdCARs were stained with anti-human CD28 and examined via flow cytometry for CD28 expression (left) and NeonGreen expression as a marker of CAR expression (right).

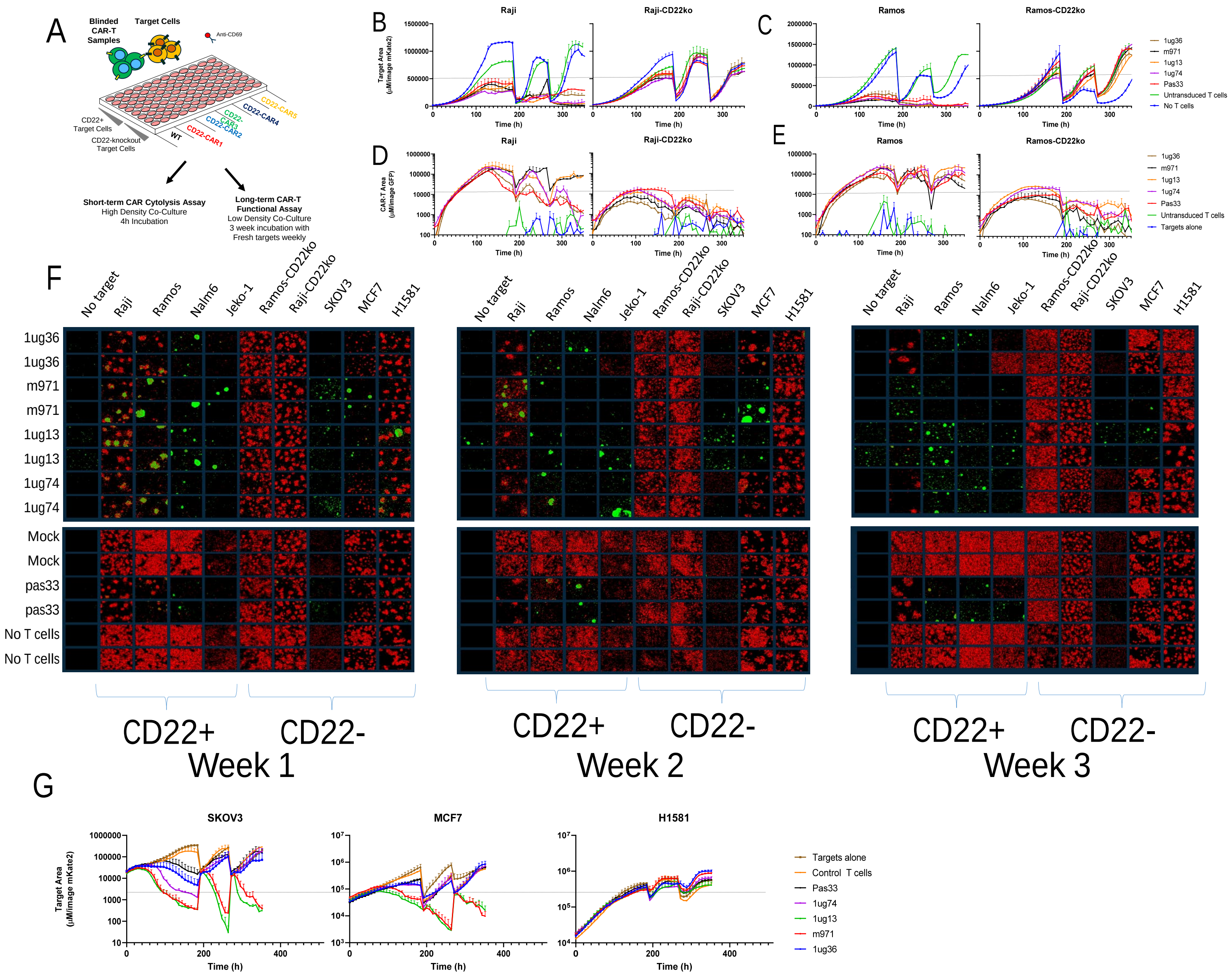

Figure S8: *Supplemental in vitro functional data for CD22-VHH CAR lead selection.* High quality CD22 CAR-T cells or untransduced control T cells were generated as described in the text. (a) CAR-T or control T cells were then assessed in blinded format via co-culture with IL7/IL15 supplementation and various target cells for functional responses using long term co-culture and live microscopy wherein cultures were split weekly with addition of fresh target cells. Co-cultures with Raji, Raji-CD22ko, Ramos, and Ramos-CD22ko cells were examined for (b-c) target cell growth or (d-e) CAR-T expansion. (f) Endpoint images of co-cultures before media exchange and addition of fresh target cells are shown at day 7, 14, and 21 [green = T cells, red = target cells] (g) Target cell growth for irrelevant CD22-negative targets is shown. (h) NSG mice implanted with Ramos-FLUC cells and treated with various CAR-T cells as per description in the text, were examined for bioluminescence at various timepoints after tumour injection as shown. P values show inter-group comparisons using student T test with log transformed bioluminescence values.

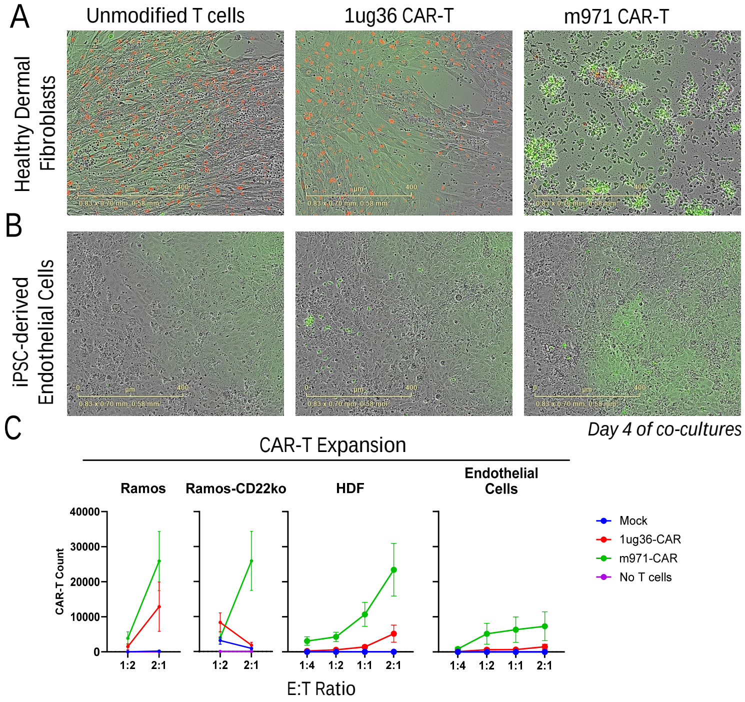

Figure S9: *Assessment of non-specific CAR-T responses against healthy donor human fibroblast and iPSC-derived endothelial cells.* 1ug36-sdAb or m971-scFv CD22 CAR-T cells or untransduced control T cells were generated as described in the text. CAR-T or control T cells were then combined at varying effector to target ratios in co-culture with human lymphoma cells (Ramos), CD22-knockout Ramos cells (Ramos-CD22ko), healthy human dermal fibroblasts (HDF), or human iPSC-derived endothelial cells. After 5 days of co-culture images were acquired to assess number of green fluorescent CAR-T or (a) red fluorescent HDF target cells. (b) Similarly, co-cultures with unmarked iPSC-derived endothelial cells were also examined. (c) After image acquisition, co-cultures were also examined for total CAR-T cell numbers per well via flow cytometry. Results show the total count of cells at endpoint averaged from 3 experiments performed in duplicate +/- SEM.

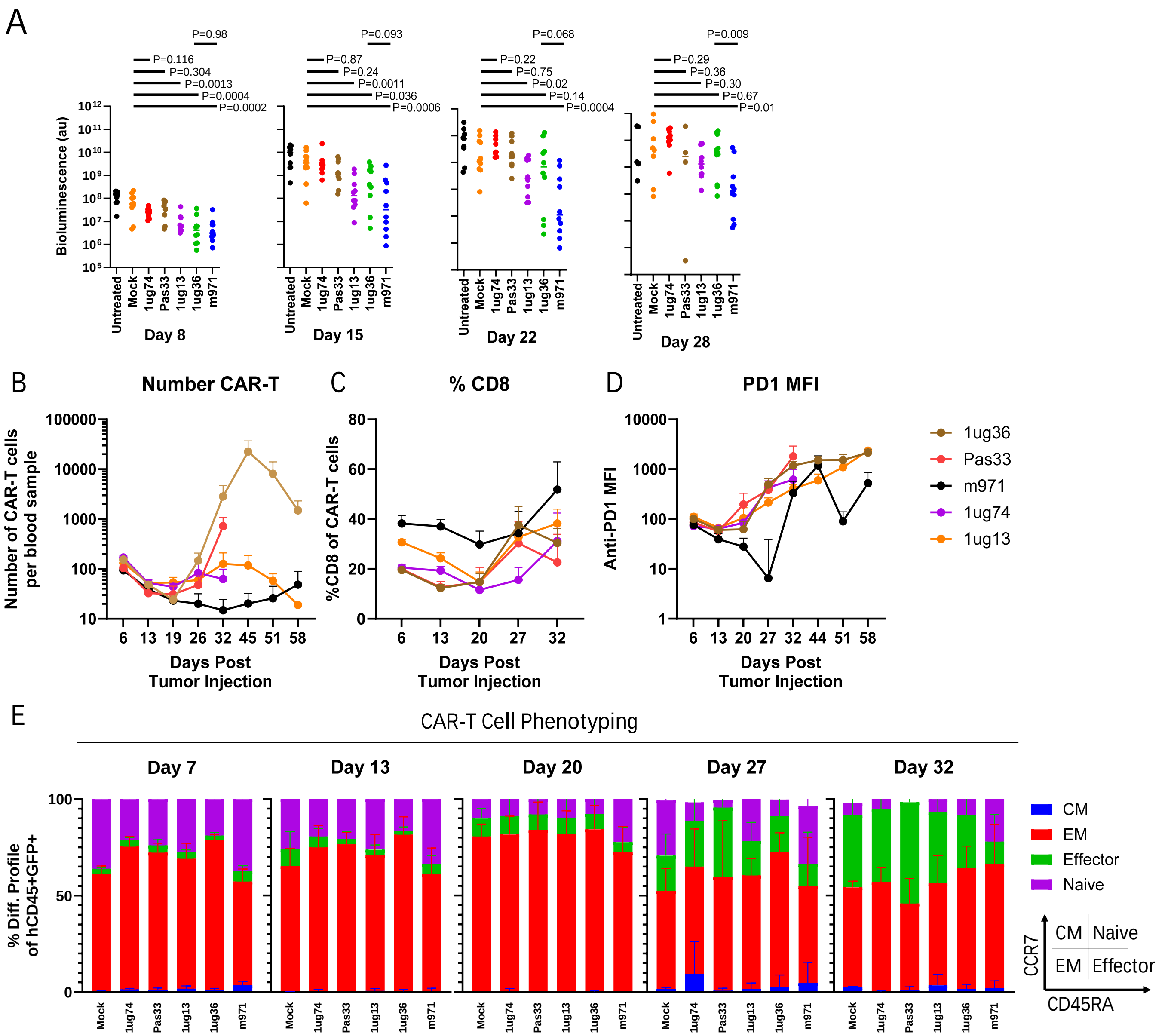

Figure S10: *Additional analysis of T cell phenotype for in vivo lead selection study.* The *in vivo* therapeutic activity of various CD22sdCAR-T cells were tested using Ramos xenograft model as described in the text (n=10 mice per group). Animals were bled at weekly intervals to examine circulating T cell phenotype via flow cytometry. (a) The mean number of circulating CAR-T cells (hCD45+/hCD19-/GFP+) is shown for CAR-T treatment groups. (b) The mean proportion of CD8+ cells within gated CAR-T cells is shown for each treatment group. (c) The average expression of PD1 within gated CAR-T population (median fluorescence intensity) is shown. (d) The differentiation phenotype of CAR-T cells, or total T cells for mic treated with unmodified T cells, was examined. Gating scheme is shown in inset (Central memory, CM = CCR7+/CD45RA+; Effector Memory, EM = CCR7/CD45Ra-, Effector = CCR7-/CD45RA+; Naïve = CCR7+/CD45RA+). Graphs show the mean values for all surviving mice at the timepoints as shown +/- SEM.

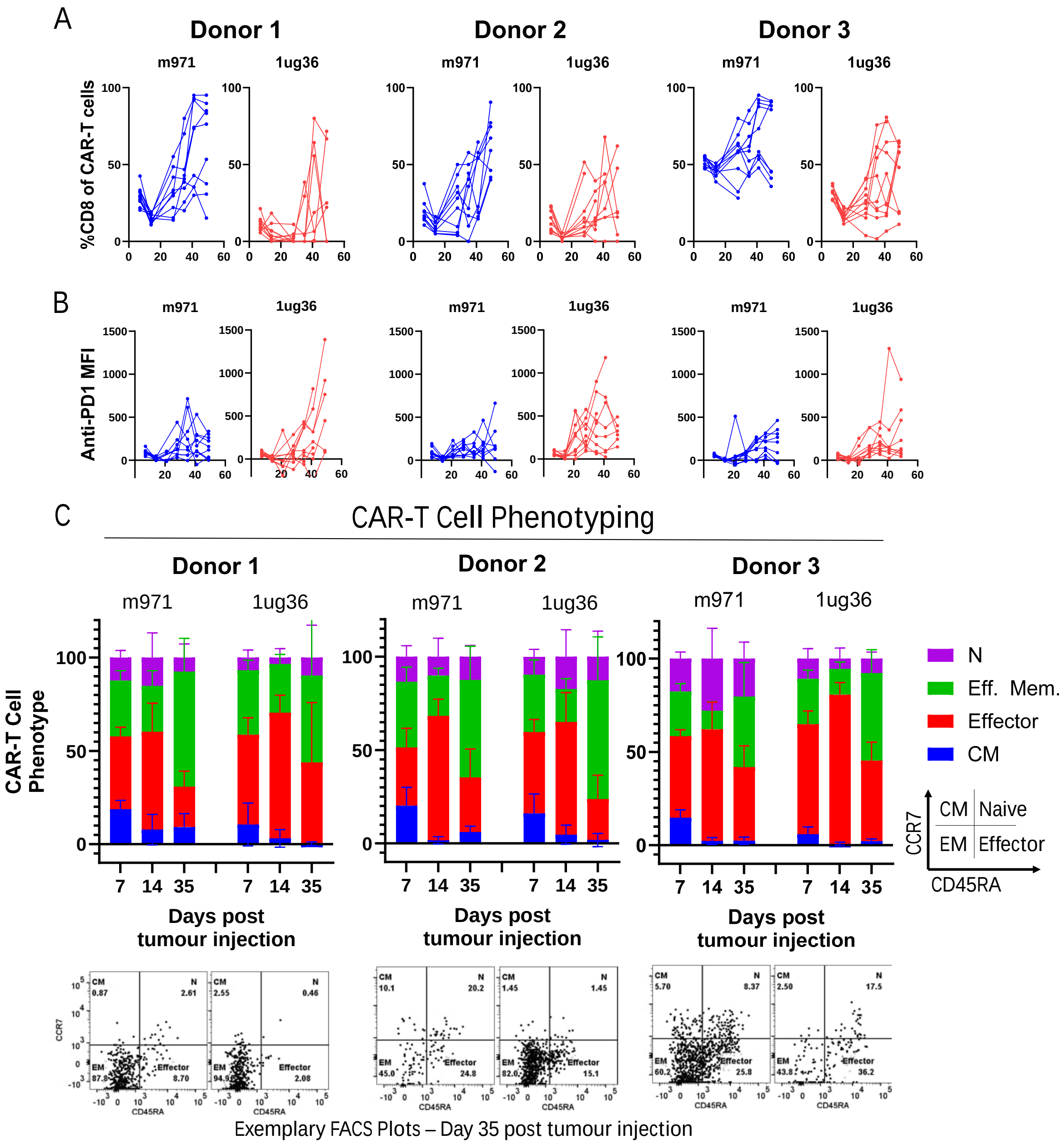

Figure S11: *Additional analysis of T cell phenotype for multi-donor in vivo lead confirmation study.* The *in vivo* therapeutic activity of 1ug36-CD22sdCAR-T or benchmark m971 CD22-scFv-CAR-T cells were tested using Ramos xenograft model as described in the text (n=10 mice per group). Animals were bled at various timepoints as shown to examine circulating T cell phenotype via flow cytometry. (a) The mean proportion of CD8+ cells within gated CAR-T cells (hCD45+/hCD19-/CD22-binding+) is shown for each treatment group. (b) The average expression of PD1 within gated CAR-T population (median fluorescence intensity) is shown. (c) The differentiation phenotype of CAR-T cells was examined. Gating scheme is shown in inset (Central memory, CM = CCR7+/CD45RA+; Effector Memory, EM = CCR7/CD45Ra-, Effector = CCR7-/CD45RA+; Naïve = CCR7+/CD45RA+). Exemplary scatterplots from day 35 post-tumour injections is shown below the bar graphs. Graphs show the mean values for all surviving mice at the timepoints as shown +/- SEM.

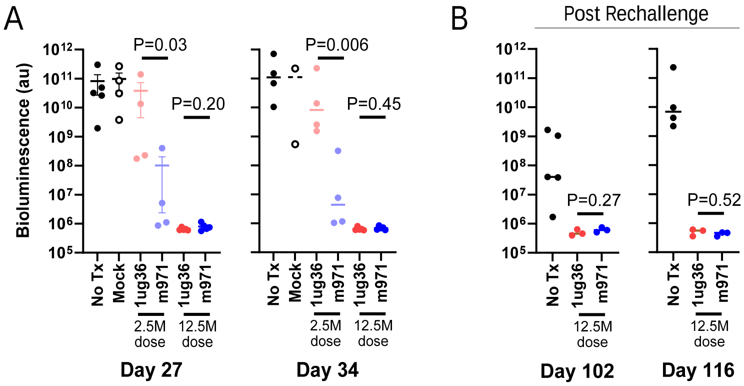

Figure S12: *Comparison of tumour load measurement via bioluminescent in vivo imaging for CD22 CAR-T dose response study*. NSG mice engrafted with $5x10^4$ Ramos-FLUC cells and treated with marginal or high dose of 1ug36 or m971 CD22 CAR-T cells as per description in the main text (N=5 mice per group). (a) Mice were examined for tumour load at day 27 and 34 post tumour injection via bioluminescent imaging and the signal was quantitated per mouse. (b) Surviving high dose CAR-T mice were rechallenged with $5x10^4$ Ramos-FLUC cells at day 85 post tumour injection and examined for tumour load at day 102 and 116 after initial tumour injection. P values show inter-group comparisons using student T test with log transformed bioluminescence values.

Table S1: *Summary of immunochemical and competitive binding characteristics for CD22-sdAbs* is provided based on the observations in surface plasmon resonance experiments performed as described in the text.

Seq. Family	sdAb Name	k_a (1/Ms)	k_d (1/s)	K_D (nM)	sdAb Used for Binning	Epitope Bin
A	1ug13	2.32E+05	6.75E-04	2.91	1ug13	Bin I
B	1ug36	1.19E+05	1.14E-03	9.59	1ug36	Bin II
C	Pas16	3.65E+05	1.00E-03	2.75	Pas16	Bin II
D	1ug77	5.50E+05	2.62E-03	4.77		Bin III
D	1ug87	1.23E+06	7.97E-04	0.65		Bin III
D	1ug75	1.40E+06	1.21E-03	0.87	1ug75	Bin III
D	1ug93	1.36E+06	1.05E-02	7.7		Bin III
E	1ug10	4.77E+05	1.76E-03	3.68	1ug10	Bin III
F	Pas79	5.14E+05	3.97E-03	7.72	Pas79	Bin III
G	1ug61	1.25E+06	6.81E-04	0.55	1ug61	Bin III
H	Pas24	1.48E+05	3.86E-03	26	Pas24	Bin IV
I	Pas32	1.58E+05	3.69E-03	23.4		Bin V
I	1ug14	3.99E+05	1.17E-05	0.03	1ug14	Bin V
J	Pas33	5.40E+04	1.40E-04	2.6		Bin VI
J	1ug74	9.25E+04	6.55E-04	7.08		Bin VI
J	1ug80	2.09E+04	6.33E-04	30.3		Bin VI
J	100ng-64	5.36E+04	1.29E-04	2.41		Bin VI
J	100ng-66	1.45E+05	1.34E-04	0.92	100ng-66	Bin VI
J	100ng-2	4.68E+04	1.30E-04	2.78		Bin VI
K	Pas64	1.08E+05	2.71E-02	250	Pas64	Bin VII

Table S2: *Summary of in vitro killing observations for CD22-CAR-T screening studies.* Target killing ranking is based on the number of red fluorescent cells observed at the end of the assay (target cells; lower is better). CAR-T expansion ranking is based on the number of green-fluorescent cells (CAR-T cells; higher is better) observed at the peak of the assay.

				Product Characteristics					*In vitro* observations									*In vivo* observations				
Screen #	CAR Design	Cell Donor	CAR-T Production Conditions	CAR ABDs Tested	Fold expansion	% CAR+	% CD4 (of CAR+)	% CD8 (of CAR+)	Figure Reference	In vitro Assay	Response Ranking - Raji		Response Ranking -		Response Ranking –		Additional	Figure References	Groups Tested	Number of mice per	Median Survival	Additional Notes
											Target Killing	CAR-T	Target Killing	CAR-T	Target Killing	CAR-T						
Screen 1	VHH-(G4S)3-CD8h-CD28TM-41BB-CD3z	Blood Donor 1<br>Female - 36	LV MOI: 10<br>Medium: StemCell ImmunoCult-XF<br>Cytokine: IL-2<br>Fresh or Frozen: Fresh<br>Harvest day: 10	Untransduced<br>FMC63-scFv<br>1ug13<br>1ug61<br>1ug36<br>1ug10	291.8<br>223<br>361.5<br>351<br>318.5<br>142.8	0<br>3.78<br>27.8<br>27.8<br>28.5<br>32.6	33.8<br>27.7<br>18.8<br>19.4<br>22.7<br>19.2	33.1<br>62<br>75.8<br>75.1<br>70.7<br>74.2	Figure 3A<br>Supp. Figure 4	5:1 E:T with IL2	FMC63*<br>1ug13*<br>1ug10*<br>1ug61*	1ug10<br>FMC63*<br>1ug13*<br>1ug61*	1ug10<br>1ug13<br>FMC63<br>1ug61	1ug10<br>1ug36<br>1ug13<br>FMC63	1ug36<br>1ug10*<br>1ug61*<br>1ug13	Minimal for all		Supp. Figure 5	No treatment<br>Untransduced<br>FMC63-scFv<br>1ug13<br>1ug36	10<br>10<br>10<br>10<br>10	23<br>23<br>26<br>26<br>31	
Screen 2	VHH-(G4S)3-CD8h-CD28TM-41BB-CD3z	Blood Donor 2<br>Male - 25	LV MOI: 10<br>Medium: StemCell ImmunoCult-XF<br>Cytokine: IL-2<br>Fresh or Frozen: Fresh<br>Harvest day: 9	Untransduced<br>1ug61<br>1ug36<br>1ug10<br>1ug74<br>1ug14<br>1ug6<br>Pas33	614<br>448<br>646<br>470<br>485<br>390<br>363<br>597	0<br>19.1<br>31.3<br>23.3<br>26.7<br>14.6<br>22.6<br>26.8	48.6<br>44.2<br>40.8<br>39.4<br>43.2<br>39.4<br>37.7<br>37.7	45.2<br>45.6<br>50.2<br>54.4<br>49.1<br>52<br>52.8<br>56.5	Figure 3B	5:1 E:T with IL2	1ug36<br>1ug14<br>1ug74<br>1ug10<br>1ug6<br>1ug61<br>Pas33	1ug36<br>1ug10<br>1ug14<br>1ug6<br>1ug74<br>Pas33*<br>1ug61*	Pas33<br>1ug36*<br>1ug74*<br>1ug14<br>1ug10*<br>1ug6*<br>1ug61	1ug36<br>Pas33<br>1ug6<br>1ug74<br>1ug6<br>1ug10<br>1ug61	1ug36<br>Pas33<br>1ug14<br>1ug61<br>1ug10<br>1ug6<br>1ug74	Minimal for		Not done				
Screen 3	VHH-(G4S)3-CD8h-CD28TM-41BB-CD3z	Blood Donor 3<br>Female- 41	LV MOI: 10<br>Medium: StemCell ImmunoCult-XF<br>Cytokine: IL-7/IL-15<br>Fresh or Frozen: Fresh<br>Harvest day: 9	Untransduced<br>m971-scFv<br>1ug13<br>1ug36<br>1ug74<br>1ug6<br>Pas33	13<br>86.7<br>86<br>66<br>92<br>74<br>100	0<br>60.3<br>87.6<br>90.6<br>90.8<br>89<br>89.9	30.4<br>58.5<br>28.4<br>35.6<br>43.2<br>39.9<br>41.3	61.4<br>36.8<br>57.7<br>59.2<br>52.5<br>52.6<br>54.6	Figure 3C-E	25:1 E:T with IL7/15	1ug36<br>m971<br>1ug74<br>1ug13*<br>Pas33*<br>1ug6*	1ug74<br>m971<br>Pas33<br>1ug36<br>1ug13*<br>1ug6*	1ug36<br>m971<br>Pas33*<br>1ug74*<br>1ug6*<br>1ug13*	m971<br>1ug74<br>1ug36<br>Pas33<br>1ug13<br>1ug6	Minimal for all	Minimal for all		Not done				
Screen 3	VHH-(G4S)3-CD8h-CD28TM-41BB-CD3z	Blood Donor 4<br>Male - 33	LV MOI: 10<br>Medium: StemCell ImmunoCult-XF<br>Cytokine: IL-7/IL-15<br>Fresh or Frozen: Fresh<br>Harvest day: 9	Untransduced<br>m971-scFv<br>1ug13<br>1ug36<br>1ug74<br>1ug6<br>Pas33	50<br>60<br>114<br>100<br>130<br>20<br>158	0<br>39<br>81.8<br>82.5<br>85.1<br>83.1<br>85.5	19.1<br>39.9<br>21.4<br>23.2<br>21.4<br>21.1<br>22.8	73<br>55.8<br>73.1<br>72.7<br>72.2<br>72.4<br>68.9	Figure 3C-E	25:1 E:T with IL7/15	1ug74*<br>Pas33*<br>1ug13*<br>1ug36*<br>m971*<br>1ug6	1ug74<br>m971<br>Pas33<br>1ug36<br>1ug13*<br>1ug6*	1ug36<br>m971<br>Pas33*<br>1ug74*<br>1ug6*<br>1ug13	1ug13<br>M971*<br>1ug74*<br>1ug36*<br>Pas33*<br>1ug6*	Minimal for all	Minimal for all		Not done				
Screen 4	VHH-(G4S)3-CD8h-CD28TM-41BB-CD3z	Blood Donor 5<br>Male - 29	LV MOI: 1<br>Medium: Miltenyi TexMACs<br>Cytokine: IL-7/IL-15<br>Fresh or Frozen: Fresh<br>Harvest day: 14	1ug13<br>1ug36<br>1ug74<br>Pas33	245<br>224<br>194<br>213	52.5<br>62.7<br>58.8<br>62.4	40.1<br>41.1<br>26.8<br>33.5	27.8<br>25<br>40.1<br>37.3	Figure 4	25:1 E:T with IL7/15	Pas33<br>1ug74<br>1ug13*<br>1ug36*	Pas33<br>1ug74*<br>1ug13*<br>1ug36	1ug13<br>1ug74<br>1ug36<br>Pas33	1ug13<br>1ug74<br>Pas33<br>1ug36	Minimal for all	Minimal for all		Not done				
	VHH-CD8h-CD8TM-41BB-CD3z			Untransduced<br>m971-scFv<br>1ug13<br>1ug36<br>1ug74<br>Pas33	168<br>73.8<br>156<br>125.6<br>184<br>176	0<br>32<br>40.4<br>28.4<br>39.3<br>49.7	29.2<br>28<br>25.6<br>28.4<br>23.5<br>27	51.2<br>34.2<br>49.4<br>49.9<br>50.2<br>52		25:1 E:T with IL7/15	Pas33<br>1ug74<br>1ug13<br>1ug36<br>m971	Pas33<br>1ug74<br>1ug13<br>m971<br>1ug36	m971<br>1ug36<br>1ug74*<br>1ug13*<br>Pas33	Pas33<br>1ug13<br>1ug74<br>m971<br>1ug36	1ug13<br>Pas33<br>Minimal	1ug13<br>Pas33<br>m971<br>1ug74<br>1ug36	Much higher tonic expansion than CD28TM constructs	Figure 5	No treatment<br>Untransduced<br>m971 - 2.5M<br>m971 - 12.5M<br>1ug36 - 2.5M<br>1ug36 - 12.5M	5<br>5<br>5<br>5<br>5<br>5	34<br>27<br>58<br>>116<br>47<br>>116	Variable Dose Testing<br>Mice rechallenegd at day 85
Screen 5<br>Lead Selection Study	VHH-CD8h-CD8TM-41BB-CD3z	Leukopak donor 0<br>Male-42 years old	LV MOI: 10<br>Medium: Miltenyi TexMACs<br>Cytokine: IL-7/IL-15<br>Fresh or Frozen: Frozen<br>Harvest day: 14	Untransduced<br>m971-scFv<br>1ug13<br>1ug36<br>1ug74<br>Pas33	217<br>144<br>121<br>193<br>161<br>166	0.2<br>49.14<br>55.7<br>32.1<br>56.3<br>61.7	75.5<br>18.1<br>50.3<br>53.7<br>23.2<br>32.9	16.3<br>73.5<br>41.1<br>38.5<br>61.4<br>39.1	Supp. Figure 6<br>Figure 5	25:1 E:T with IL7/15	m971<br>1ug13<br>1ug74<br>1ug36<br>Pas33	m971<br>1ug13<br>1ug74<br>1ug36<br>Pas33	All similar	1ug13<br>Pas33<br>1ug74<br>1ug36<br>m971	Minimal for all	1ug13<br>1ug74<br>Pas33<br>m971<br>1ug36		Figure 4	No treatment<br>Untransduced<br>m971-scFv<br>1ug74<br>Pas33<br>1ug13<br>1ug36	10<br>10<br>10<br>10<br>10<br>10<br>10	29<br>29<br>>60<br>33.5<br>28<br>42<br>>60	
Screen 6<br>Multi-donor activity confirmation study	VHH-CD8h-CD8TM-41BB-CD3z	Leukopak donor 1<br>Female-22 years old	LV MOI: 10<br>Medium: Miltenyi TexMACs<br>Cytokine: IL-7/IL-15<br>Fresh or Frozen: Frozen<br>Harvest day: 14	Untransduced<br>m971-scFv<br>1ug36	704<br>592<br>560	0<br>28<br>30	59.1<br>25<br>90.3	40.9<br>75<br>9.7	Not done									Supp. Figure 8	Untreated<br>Untransduced<br>m971-scFv<br>1ug36	10<br>10<br>10<br>10	28<br>29<br>114<br>46	Mice rechallenegd at day 85
																			Untreated<br>Untransduced<br>m971-scFv<br>1ug36	10<br>10<br>10<br>10	21<br>24<br>>90<br>>90	Donor 1 Repeat<br>Mice rechallenegd at day 85
		Leukopak donor 2<br>Male-24 years old		Untransduced<br>m971-scFv<br>1ug36	848<br>624<br>896	0<br>22<br>33	77.8<br>89.8<br>93.7	22.2<br>10.2<br>6.3											Untransduced<br>m971-scFv<br>1ug36	10<br>10<br>10	32<br>92<br>52	Mice rechallenegd at day 85
		Leukopak donor 3<br>Male-28 years old		Untransduced<br>m971-scFv<br>1ug36	664<br>440<br>944	0<br>25<br>38	37.5<br>61.9<br>75	62.5<br>38.1<br>25											Untransduced<br>m971-scFv<br>1ug36	10<br>10<br>10	32<br>88<br>105	Mice rechallenegd at day 85

nd: Not done; ABD: antigen binding domain;

Table S3: *Summary of observations in the human tissue array stained with CD22 1ug36-VHH or irrelevant specificity B131-VHH*. Staining was performed with anti-VHH secondary and anti-mouse tertiary as described in the methods section. Observers 1 and 2 were blinded to sample identity in stained slides and recorded their observations independently. Representative images are provided for anti-CD22 sdAb 1ug36 stained slide (slide 1) and irrelevant specificity sdAb B131 stained slide (slide 2).

See supplemental excel document available online.

Table S4: *Flow Cytometry Antibody Staining Panel used for mouse blood analysis.* A complete list of the staining panel used for analysis of circulating T cells in the in vivo lead selection and multi-donor lead confirmation studies is provide, including supplier information.

Lasers	Filters	Marker	Clone	Fluorochrome	Catalogue #	Lot #	Supplier	Storage conditions
Violet Laser (405 nm)	780/60	CD69	FN50	BV786	563834	276145	BD Biosciences	4°C
	710/50	mCD45	30F11	BV711	563709	1076257	BD Biosciences	4°C
	660/20	CD45RA	HI100	BV650	563963	1036332	BD Biosciences	4°C
	610/20	CD45RO	UCHL1	BV605	562791	1039402	BD Biosciences	4°C
	525/50	N/A	N/A	N/A	N/A	N/A	N/A	N/A
	450/40	CD279 (PD1)	EH12.1	BV421	329920	B314841	BioLegend	4°C
Blue laser (488nm)	710/50	CD8	SK1	PerCP-Cy5.5	565310	135209	BD Biosciences	4°C
	530/30	hSiglec-2 CD22-Protein-His-Tag	N/A	FITC	SI2-HF2H6-25ug	IAF2028b	Acro Biosystems (Distributor: Cedarlane, Canada)	4°C
Yellow-Green Laser (561nm)	780/60	CD25	M-A251	PE-Cy7	557741	1068706	BD Biosciences	4°C
	710/50	N/A	N/A	N/A	N/A	N/A	N/A	N/A
	610/20	N/A	N/A	N/A	N/A	N/A	N/A	N/A
	586/15	CD197 (CCR7)	3D12	PE	552176	184671	BD Biosciences	4°C
Red Laser (640nm)	780/60	hCD45	2D1	APC-H7	560178	274948	BD Biosciences	4°C
	730/45	N/A	N/A	N/A	N/A	N/A	N/A	N/A
	670/14	CD22	S-HCL-1	APC	363506	B290368	BioLegend	4°C
UV Laser (355nm)	740/35	CD27	L128	BUV737	564301	1112895	BD Biosciences	4°C
	515/30	CD19	SJ25C1	BUV496	612938	233664	BD Biosciences	4°C
	379/28	CD4	SK3	BUV395	563550	9273692	BD Biosciences	4°C
